# Densely-functionalized bicyclic cyclopentanones by combined photoinduced 6-*endo-trig* Giese additions and mild aldol cyclizations

**DOI:** 10.1039/d5qo01635e

**Published:** 2026-01-27

**Authors:** Júlia Viñas-Lóbez, Nicolas Sellet, Bibiana Fabri, Guillaume Levitre, Adiran de Aguirre, Amalia I. Poblador-Bahamonde, Céline Besnard, Jérôme Lacour

**Affiliations:** a Department of Organic Chemistry, University of Geneva Quai Ernest Ansermet 30 CH-1211 Geneva 4 Switzerland Jerome.Lacour@unige.ch; b Laboratory of crystallography, University of Geneva Quai Ernest Ansermet 24 CH-1211 Geneva 4 Switzerland

## Abstract

Polycyclic scaffolds are central to numerous natural products and pharmaceuticals, motivating concise, stereocontrolled routes to their construction. We report a photoredox-enabled synthesis of *trans*-fused [*n*.3.0] bicyclic ketones (*n* = 4, 5, 10) from malonate-derived enol ethers. α-Brominated intermediates, formed by acylation with 2-bromo-2-methylpropanoyl bromide, undergo radical cyclization under two complementary conditions: (i) acridinium orange (AOH^+^) with Hantzsch ester (HE) at 455 nm, or (ii) photoexcited HE alone at 365 nm. Both modes trigger 6-*endo-trig* Giese addition, C–O bond fragmentation, and hydrogen-atom transfer to α-branched cyclic ketones that ring-close under mild Brønsted or Lewis acid activation to *trans*-fused products with exclusive junction control. Mechanistic studies (Stern–Volmer, UV–Vis, electrochemistry) support two activation pathways—AOH^+^* quenching by HE or direct HE excitation—each generating the same radical intermediates *in fine*. DFT calculations validate mechanistic pathways and regioselectivity in favor of philicity matching of the electrophilic radical and the polar electron-rich enol ether. The method accommodates ring-size diversity, accesses *trans*-hydrindanone architectures, and outcompetes 5-*exo-trig* spirocyclization.

## Abstract 2

A photoredox-enabled synthesis of *trans*-fused [*n*.3.0] bicyclic ketones *via* 6-*endo-trig* radical additions and mild aldol cyclizations, offering stereocontrolled access to densely functionalized bicyclic scaffolds.

## Graphical abstract

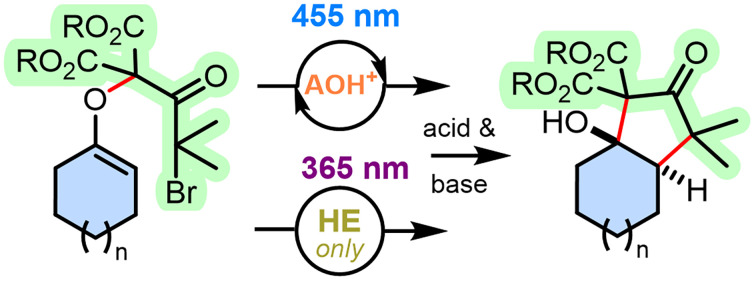

## Introduction

**Scheme 1:**
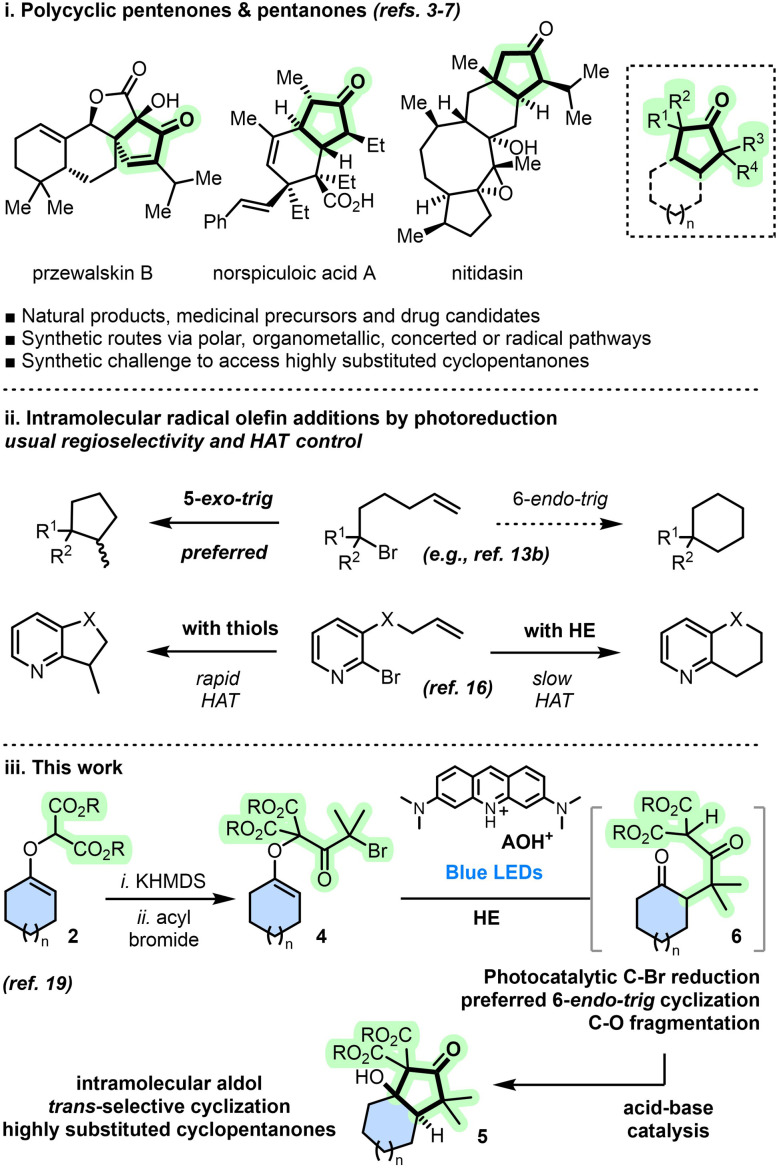
Polycyclic pentenones and pentanones. Photoreductive radical cyclizations. Synthetic strategy toward highly substituted [*n*.3.0] bicyclic derivatives. AOH^+^ = acridinium orange photocatalyst.

Polycyclic natural products and medicinal drugs are ubiquitous generating hence a continuous demand for novel cyclization strategies in academic and industrial laboratories alike.^1^ Methodologies for ring closures are thus crucial and very diverse approaches are available, from polar bond formations to concerted, organometallic or radical (*vide infra*) pathways.^2^ Cyclopentenones,^3^ and related saturated cyclopentanones,^4^ are key structural subunits often included in medicinal precursors of drug candidates,^5^ or as part of polycyclic natural products, such as przewalskin B,^6^ norspiculoic acid A or nitidasin^7^ (Scheme 1i). In the cyclopentanone series, generation of highly-substituted (hindered) skeletons remains a general challenge requiring, in addition, the stereocontrol of *trans*- or *cis*-fused junctions.^8^

As mentioned earlier, radical-mediated synthesis of polycyclic molecules is a recognized strategy using, most often, intramolecular Giese reactions of carbon-centered radicals onto neighboring olefin(s).^9^ Conditions of radical formation and the size of the resulting ring(s) are key parameters that control addition regio- and stereoselectivity.^10^ Cyclizations leading to the formation of 5- or 6-membered rings have been particularly studied.^11^ Reliable prognostics on such cyclizations are possible thanks to many seminal contributions and Beckwith's radical rules in particular.^12^ Also, visible light-induced and photoredox catalyzed reactions have transformed the field of radical-mediated processes leading to a large array of novel synthetic methodologies.^13^ In this regard, formations of cyclized products by intramolecular additions after photoreduction of C–X bonds (X = halogen, chalcogen, *etc*.) are key.^13*b*,14^ Most often, *n-exo-trig* regioselectivity is observed primarily, over *n* + 1-*endo-trig* pathways (Scheme 1ii).^15^ Of note, Blakey *et al.* reported a switchable regioselective 6-*endo*/5-*exo* photoredox catalyzed cyclization.^16^ The selectivity arose from different HAT rates; the 5-*exo* product being obtained with a polarity-matched thiol HAT process, while using Hantszch ester (HE) gave the 6-*endo* product.

Previously, using cyclic ketones 1 and α-diazomalonates^17^ as reactants (Scheme S1), a direct synthesis of malonate enol ethers 2 was reported under [CpRu(N≡CCH_3_)_3_][BAr_F_] catalysis;^18^ the resulting adducts being readily functionalized under basic conditions to afford malonate alkylation products.^19^ Herein, to harness regioselective radical reactivity and later achieve densely-functionally cyclopentanones, the acylation of enol ethers 2 with 2-bromo-2-methylpropanoyl bromide 3 was pursued to produce substrates of type 4. Subsequent photoredox conditions afford α-branched ketones that cyclize under mild acidic conditions to form densely-functionalized [*n*.3.0] bicyclic products 5 (*n* = 4, 5, 10) (Scheme 1iii). This novel cyclization sequence, involving two independent C–C bond forming reactions and one original C–O cleavage, generates *trans*-fused bicyclo[4.3.0]nonanes exclusively. Mechanistic studies reveal a dichotomic nature of the photoredox initiation and a possible competition between 5-*exo-trig* and 6-*endo-trig* pathways, generally in favor of the second by virtue of radical philicity matching.^20^ An unusual C–O fragmentation and hydrogen atom transfer (HAT) reactions liberate α-branched cyclic ketones of type 6 that are ideally suited for intramolecular aldol/ring closure reactions happening under mild acidic conditions with *trans*-selectivity only.

## Results and discussion

**Scheme 2:**
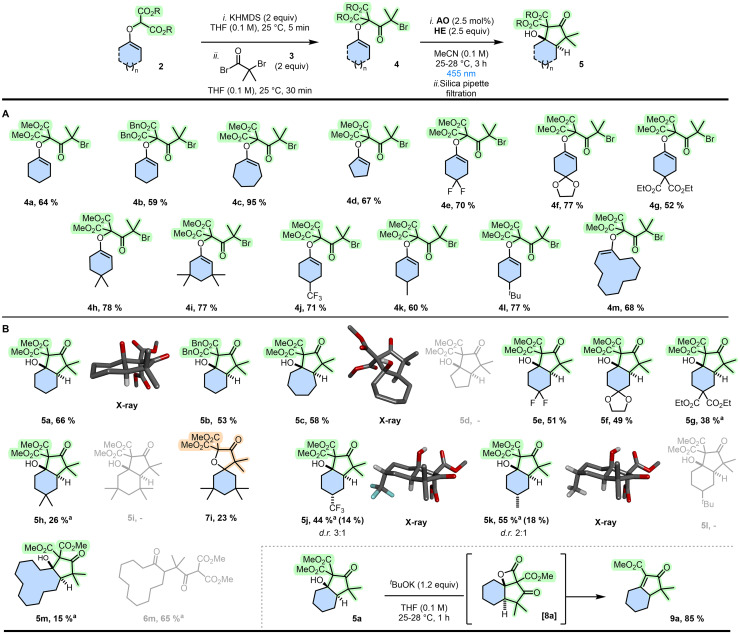
(A) Acylated malonate enol ethers 4a to 4m. (B) Hydroxylated *trans*-bicycles 5a to 5m. ^*a*^ Yields determined by ^1^H-NMR spectroscopy using 1,3,5-trimethoxybenzene as internal standard. Isolated yield of the major diastereomer in parenthesis. In grey, lack of product formation or open-armed product obtained.

In the context of cyclization strategies, it was then enticing to use compounds 2 and their malonate subunits to introduce halogenated chains under basic conditions (substrates 4, Table 1 and Scheme 2) and study subsequent reductions under photoredox conditions. Upon generation of electrophilic α-keto radicals, ring formation ought to occur onto the nucleophilic end of the enol ether moiety providing an effective discrimination and a preferred 6-*endo* regioselectivity. Preliminary studies were performed using enol ether 4a made from 2a by treatment with KHMDS (2.0 equiv.) at 25 °C. After rapid and quantitative deprotonation of the malonate group, addition of 2-bromo-2-methylpropanoyl bromide 3 (1.5 equiv.) afforded adduct 4a (64%). Then, under blue LED irradiation (*λ*_max_ 455 nm) using Ir(ppy)_3_ (2.5 mol%) as photocatalyst (PCat) and diisopropylethylamine (DIPEA) as reductant and hydrogen donor (10 equiv.), the targeted reactivity was immediately observed in acetonitrile at 25–28 °C (Table 1, entry 1). ^1^H NMR analyses of crude reaction mixtures were initially ambiguous as large differences were observed between spectra measured before and after a short filtration over a SiO_2_ plug.^21^ In the latter case, spectra revealed an excellent conversion of 4a to major product 5a obtained as a single stereoisomer. This product was isolated upon silica gel chromatography (43%). Yet, NMR investigations of 5a were not agreeing with structures derived from simple 5-*exo-trig* or 6-*endo-trig* cyclization pathways. The connectivity indicated the occurrence of a rearrangement that could only be untangled upon X-ray analysis (CCDC 2058766). Single crystals of 5a were obtained by diffusion in a mixture of methylene chloride and pentane. The structural determination revealed a [4.3.0] bicycle with two *trans*-fused carbocycles; a cyclohexane and a densely-functionalized cyclopentanone. Care was then taken to unravel the reactivity prior to the silica gel treatment, *i.e.* before the cyclization to 5a. All crude NMR data pointed toward α-branched cyclohexanone 6a, which cannot be isolated. Further analysis of the crude mixture indicated, in retrospect, the presence of a minor component corresponding to spiro bicycle 7a (4–18%, Table 1). The origin of this moiety will be later discussed during the mechanistic analysis but such an occurrence was expected in view of the usual predominance of 5-*exo-trig* cyclization pathways and also results previously-obtained with malonate enol ethers.^19^ Given the unusual skeletal rearrangement of 4a to 6a and then *trans*-5a, the reactivity was investigated further. First, a screen of experimental conditions (Table S1) was performed to improve conversions and yields, an excerpt is detailed in Table 1. Substitution with [Ir(dFCF_3_ppy)_2_(bpy)]PF_6_ as photocatalyst increased the yield up to 50% (entry 2). Additional amounts of HE (5 equiv.) were beneficial (5a, 60%, entry 3). In effect, HE (10 equiv.) could substitute DIPEA entirely (5a, 72%, entry 4). Classical Ir(ppy)_3_ could be used with only 2.5 equivalents of HE to form 5a in 70% (entry 5). Various organic dyes absorbing in blue-green domains were also tested as photocatalysts (entries 6–8, and Table S1), including rose Bengal or eosin Y giving yields of 60% and 68%, respectively (entries 6 and 7). With mildly-reducing acridine orange (AO), to a bit of our surprise, bicycle 5a was still obtained in an effective yield (72%, entry 8); a rationale will be provided later (*vide infra*). Further variations of different Hantzsch esters, solvents, catalyst loading and substrate concentration, did not lead to any additional improvements (Table S1). As expected, reactions do not proceed in the absence of HE or blue light (entries 9 and 10). Without AO, 5a is nevertheless obtained in a surprising 19% yield, suggesting that HE can also play a photoreducing role (entry 11); this reactivity will be later exploited. Nonetheless, entry 8 combining (i) visible light photoredox catalysis and (ii) a silica gel filtration represented the most effective conditions to 5a, which were selected for the remainder of the study.

**Table 1:** Optimization of the reaction conditions^a^

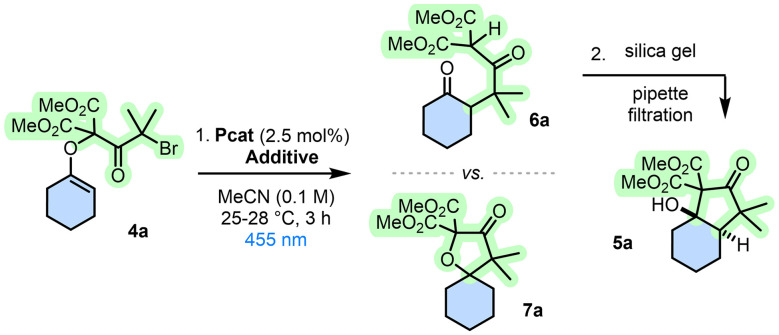				
Entry	Photocatalyst (PCat)	Additive	Yield 5a (%)	Ratio (6a/7a)^b^
1	Ir(ppy)_3_	DIPEA (10 equiv.)	48 (43)^c^	85/15
2	[Ir(dFCF_3_ppy)_2_(bpy)]PF_6_	DIPEA (10 equiv.)	50	90/10
3	[Ir(dFCF_3_ppy)_2_(bpy)]PF_6_	DIPEA (10 equiv.), HE (5 equiv.)	60	82/18
4	[Ir(dFCF_3_ppy)_2_(bpy)]PF_6_	HE (10 equiv.)	72	91/9
5	Ir(ppy)_3_	HE (2.5 equiv.)	70	92/8
6	Rose bengal	HE (2.5 equiv.)	60	96/4
7	Eosin Y	HE (2.5 equiv.)	68	91/9
**8**	**Acridine orange**	HE**(2.5 equiv.)**	**72 (66)** c	**92/8**
9	Acridine orange	None	Traces	—
10	Acridine orange	HE (2.5 equiv.)	n.r.^d^	—
11	None	HE (2.5 equiv.)	19	92/8

a Reaction conditions: 4a (0.05 mmol), PCat (2.5 mol%), additive, MeCN (0.1 M), 25–28 °C, blue LEDs, 3 h. Yields determined by ^1^H-NMR spectroscopy using 1,3,5-trimethoxybenzene as internal standard. DIPEA: *N*,*N*-Diisopropylethylamine.

b Ratios between 6a and 7a were determined by ^1^H NMR analysis.

c Isolated yields in parenthesis.

d Without light. n.r.: no reaction.

In effect, a series of α-bromo *gem*-dimethyl ketones 4a to 4m were prepared from the corresponding malonate enol ethers (Scheme 2A). Overall, the acylation was effective (52–95% yields) under irreversible deprotonation conditions (KHMDS). Then, using conditions from Table 1 (entry 8), *trans*-fused bicyclic compounds 5 were obtained in low to good yields (15–66%, Scheme 2B).^22^ First, with 4a, the reaction was conducted in 1 mmol scale and proceeded without loss of conversion to afford 5a with a comparable isolated yield (66%). However, performed on a gram scale of starting bromide, the isolated yield of 5a decreased to 50%. Benzyl malonate enol ether 4b reacted similarly to give 5b in slightly lower 53% yield, a possible consequence of the increased steric hindrance. 7-Membered ring 5c formed efficiently (58%). The *trans* ring fusion was ascertained by X-ray diffraction analysis again (CCDC 2481640, Scheme 2). In the case of cyclopentanone-derived 5d, which was unproductive, the elevated strain associated with a *trans*-fused [3.3.0] structure^23^ is probably responsible for the lack of ring closure observed.^24^ 4,4′-Difluorinated enol ether 4e and dioxolane 4f reacted to yield 5e and 5f in 51% and 49% yields, respectively. However, *gem*-disubstituted 4g and 4h afforded the corresponding dimethylated and dicarboxyethylated products 5g and 5h in lower 38 and 26% (NMR yields), respectively. Overall, despite a lower productivity for diester 5g, the presence of (inductive) electron-withdrawing groups at 4,4′-positions seems more favorable. However, strong steric effects preclude cyclization as hexamethylated 5i could not be formed. Instead of *trans*-fused rings, 5-*exo-trig* cyclization is preferred forming the spirocycle adduct 7i (30% NMR and 23% isolated yields). The diastereoselectivity of the cyclization was briefly studied with 4-trifluoromethyl and 4-methyl-substituted enol ethers 5j and 5k. *trans*-Bicycles 5j (44% NMR yield) and 5k (55% NMR yield) were obtained as mixtures, with respective 3 : 1 and 2 : 1 diastereomeric ratios (dr). Only the major isomers could be isolated upon chromatography (14% and 18%, respectively); their structures (CCDC 2481641 and 2481639) presenting the 4-CH_3_ or 4-CF_3_ groups in axial positions. A rationale for the observed stereochemistry is provided in the SI (Scheme S4).^25^ However, when the substrate is again strongly hindered or biased sterically like 5l with the *tert*-butyl substituent, a lack of desired reactivity is obtained. Finally, of importance for the mechanism, [10.3.0] bicycle 5m was obtained from 12-membered 4m in a low ^1^H-NMR yield (15%) but alongside 6m (63%) as major product. This derivative 6m is not prone to enolization and cyclization. Yet, this observation of 6m led us to check how general was the formation of open-arm molecules. In the six-membered ring series, crude mixtures from reactions of 4a and 4f were verified immediately after irradiation, only to confirm the presence of α-functionalized cyclohexanones in all instances. The photoredox cyclization therefore stops at the C–O bond fragmentation and the formation of adducts of type 6. With these mixtures in hand, a small amount of silica gel (pipette filtration) was sufficient to provoke the intramolecular aldol to 5a and 5f.^26^ Nevertheless, condensations were more reliable under soft-enolization Lewis acid–base conditions.^27^ In fact, full conversion was obtained with either MgCl_2_/Et_3_N^28^ or LiCl/DBU^27*a*^ to yield 5a (66%) in 15 and 30 minutes from 6a, respectively. Finally, care was taken to evaluate the reactivity of the *trans*-fused products of type 5 only to find that the tertiary alcohol group is unreactive under dehydration (*e.g.*, Burgess reagent^29^) or alkylative conditions (*e.g.*, benzyl 2,2,2-trichloroacetimidate^30^). However, upon addition of ^*t*^BuOK at 25 °C, hydroxydecarboxylative elimination occurs. Mechanistically, it is proposed that β-lactone [8a] is formed and leads to 9a (85%) upon strain release and CO_2_ cleavage.

**Fig. 1:**
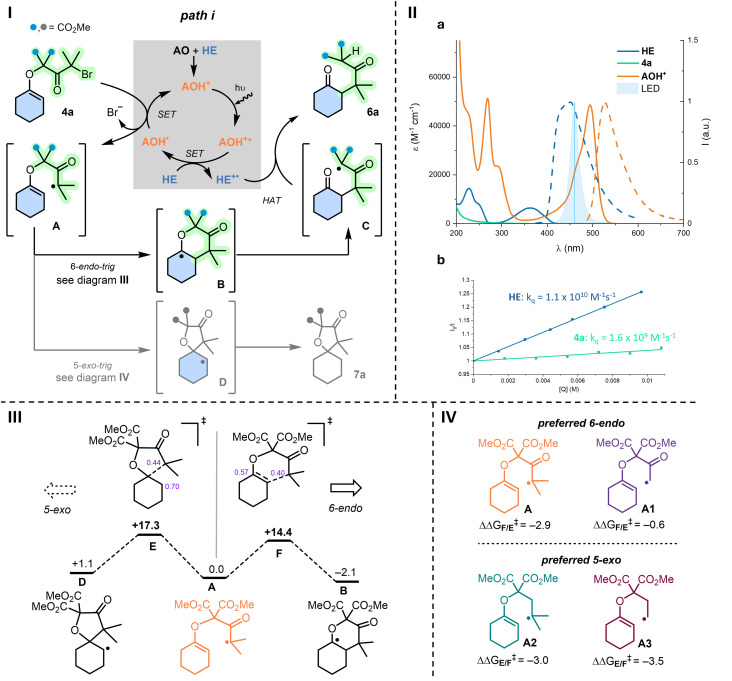
(I) Synthesis of open-chained ketone 6a and spiro derivative 7a*via* 6-*endo-trig* (major, black) and 5-*exo-trig* (minor, grey) closures under photocatalysis (path *i*, AOH^+^, *λ*_exc_ 455 nm). (II) (a) Absorption and normalized emission spectra of reaction components in air-equilibrated acetonitrile 20–23 °C with concentrations 1 × 10^−5^ to 5 × 10^−6^ M and (b) Stern–Volmer plot of the luminescence quenching of AOH^+^ upon increasing concentration of quencher. Quenching experiments were conducted in dry acetonitrile under N_2_ atmosphere with AOH^+^ (1 × 10^−5^ M), the emission intensity was followed at *λ* = 529 nm; *I*_0_ is the intensity before and *I* after adding the quencher, [Q] stands for concentration of the quencher, in which Q is either HE (path *i*, this figure) or 4a (path *ii*, Scheme S2). (III) Gibbs free energy analysis of 5-*exo vs.* 6-*endo* cyclizations from first radical A to bicyclic B or spiro D radicals, and respective transition states F and E. (IV) Comparison of A with theoretical A1, A2 and A3 radicals, and subsequent regioselectivity (ΔΔ*G*^‡^, kcal mol^−1^).

In parallel, insight into the photocatalytic mechanism (4a 1.0 equiv., AO 2.5 mol% and HE 2.5 equiv.) was looked for (Fig. 1). Optical and electrochemical data of the reactants provided possible energy pathways upon light excitation (see SI).^31^ Absorption and emission spectra of AO (Fig. S1) afforded an optical energy gap (E_00_) of 2.62 eV, with a reported reduction potential in acetonitrile of −2.0 V *vs.* SCE.^32^ As a consequence, the excited state AO* has *E*^red^ = +0.62 V (*vs.* SCE) and cannot perform the necessary oxidation of HE (*E*^ox^ = +0.93 V *vs.* SCE) by single electron transfer (SET). The overall process is unproductive (Δ*G*_0_ > 0, see SI).^33^ After this observation, the UV-Vis absorption spectrum of the crude reaction was analyzed further and it revealed a lower energy signal upon reaction of AO with HE in excess, which was matched with protonated acridinium AOH^+^ (see Fig. 1IIa). This cationic species possesses all the necessary properties in its excited state to perform photocatalysis (E_00_ = 2.44 eV, *E*^red^ (AOH^+^*/AOH˙) = +1.72 V and *E*^ox^ (AOH^++^˙/AOH^+^*) = −1.24 V *vs.* SCE) (Tables S2 and S3). In effect, by irradiating at 455 nm, AOH^+^ is efficiently excited and two quenching pathways are then energetically possible, either (*i*) a SET involving the oxidation of HE or (*ii*) the direct reduction of enol ether 4a (*E*^red^ = −1.0 V *vs.* SCE). In support, with Stern–Volmer experiments, quenching of AOH^+^ luminescence (*λ*_em_ = 525 nm, *Φ* = 37% and *τ* = 2.5 ns in acetonitrile) is detected for both HE and enol ether 4a with kinetic quenching constants (*k*_q_) of 1.1 × 10^10^ M^−1^ s^−1^ and 1.6 × 10^9^ M^−1^ s^−1^, respectively (Fig. 1IIb). While quenching of HE is 10-fold faster than that of the enol ether 4a, quenching efficiencies (*η*) are similar with values of 28% and 20%, taking into account the available concentrations of HE and 4a in solution during the photocatalytic reaction (Fig. S10–S13).^34^ Overall, path *i* (Fig. 1) remains more favorable than path *ii* (Scheme S2) and the difference in efficiencies increases over time as 4a is progressively consumed (Fig. S12). In any case, satisfactorily, both paths *i* and *ii* lead to the same product. With path *i*, shown in Fig. 1I, after formation of AOH˙ by quenching of AOH^+^* with HE, reduction of substrate 4a by SET leads to α-keto radical A.^44^ With path *ii*, displayed in Scheme S2, intermediate A is formed directly by reaction of 4a with AOH^+^*. Then, independent of its origin, A undergoes a preferred 6-*endo-trig* cyclization to form B. Subsequent C–O cleavage occurs and affords stabilized tertiary malonyl C. Such homolytic C–O cleavage seems to be original to this transformation. Then, the highly electrophilic radical cannot readily cyclize to the intramolecular δ-ketone; preferred HAT of C with either HE or protonated HE^+^ (ref. 35) must occur and yield open-chain products 6 observed in crude mixtures, for 5m in particular. In terms of regioselectivity, confirmation was brought by computational analysis of the preferred 6-*endo-trig* (A → B) over the 5-*exo-trig* (A → D) cyclization pathways, both kinetically (Δ*G*^‡^ = −2.9 kcal mol^−1^) and thermodynamically (Δ*G*° = −3.2 kcal mol^−1^) (Fig. 1III).

**Scheme 3:**
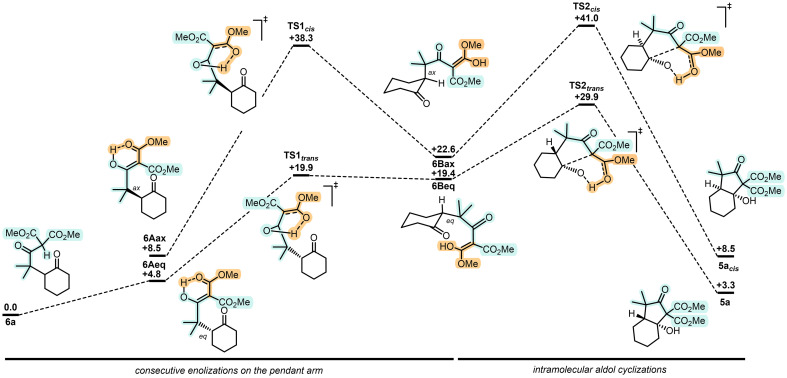
Computed reaction paths from synthetic intermediate 6a to *trans*-fused 5a and putative 5a_*cis*_. Gibbs free energies in kcal mol^−1^.

As the regioselectivity depended most probably on a philicity matching of first radical A with the enol ether moiety, care was taken to evaluate theoretically the influence of the substituents adjacent to the radical carbon center of A. DFT calculations using hypothetical intermediates A1 (*gem*-Me omitted), A2 (α-carbonyl omitted) and A3 (both elements missing) were executed and results compared to that of original A (Fig. 1IV). Energy barriers were calculated for 6-*endo* (As → Bs) or 5-*exo* (As → Ds) pathways *via* transition states Fs and Es, respectively (Fig. S15). As expected, in view of the polarization of the electron-rich alkene and its more nucleophilic terminal end, electrophilic α-carbonyl radicals A and A1 favor the formation of B and B1*via* transition states F and F1 (ΔΔ*G*^‡^_F/E_ = −2.9 and −0.6 kcal mol^−1^), respectively.^36^ Then, changing the nature of radicals from electrophilic to nucleophilic, A2 and A3 formed preferentially spiro 5-membered rings through transitions states E2 and E3 (ΔΔ*G*^‡^_E/F_ = −3.0 and −3.5 kcal mol^−1^), respectively. Then, with a clear explanation for the formation of open-chained derivatives 6, ring closures to bicyclic *trans*-fused 5 were also investigated computationally using mild Brønsted acid conditions as model (Scheme 3).^37^ Direct cyclization of ketone 6a, used as reactant, through a concerted transition state with proton-transfer and C–C bond formation occurring at the same time was unsuccessful.^38^ Then, we proceeded *via* all possible enols derived from 6a. Intermediates 6Aax and 6Aeq model most-stable enols with either axial or equatorial orientation of the pendant arm, and intermediates 6Bax and 6Beq, necessary for the reactivity, are the enols formed on the side of the malonate chains. The lower reaction pathway requires the formation of enol 6Beq prior to the intramolecular aldol. Its geometry favors a pre-organization of the arm leading to a late 6-membered transition state (TS2-*trans*) that achieves the *trans*-fused bicycle 5a by the lower activation barrier computed so far (Δ*G*^‡^ = +29.9 kcal mol^−1^).^39^ In fact, without the presence of enol 6Beq, the cyclization from 6Aeq was located at +50.6 kcal mol^−1^ as it models a less favored 7-membered ring transition state (Scheme S3). For good measure, analogous transition state TS2-*cis*, which would be necessary to reach a *cis*-fused bicycle, was located at 41.0 kcal mol^−1^, more than 10 kcal mol^−1^ higher than TS2-*trans*, in agreement with experimental observation. Additionally, formation of precursor 6Bax is strongly unfavored due to the required enol geometrical isomerization upon proton transfer from 6Aax. This motion breaks the π-delocalization and the H⋯O interaction, *via* TS1-*cis* (Δ*G*^‡^ = +38.3 kcal mol^−1^) which is also far too high. Preferred formation of *trans*-fused bicycle 5a is hence justified computationally.

**Fig. 2:**
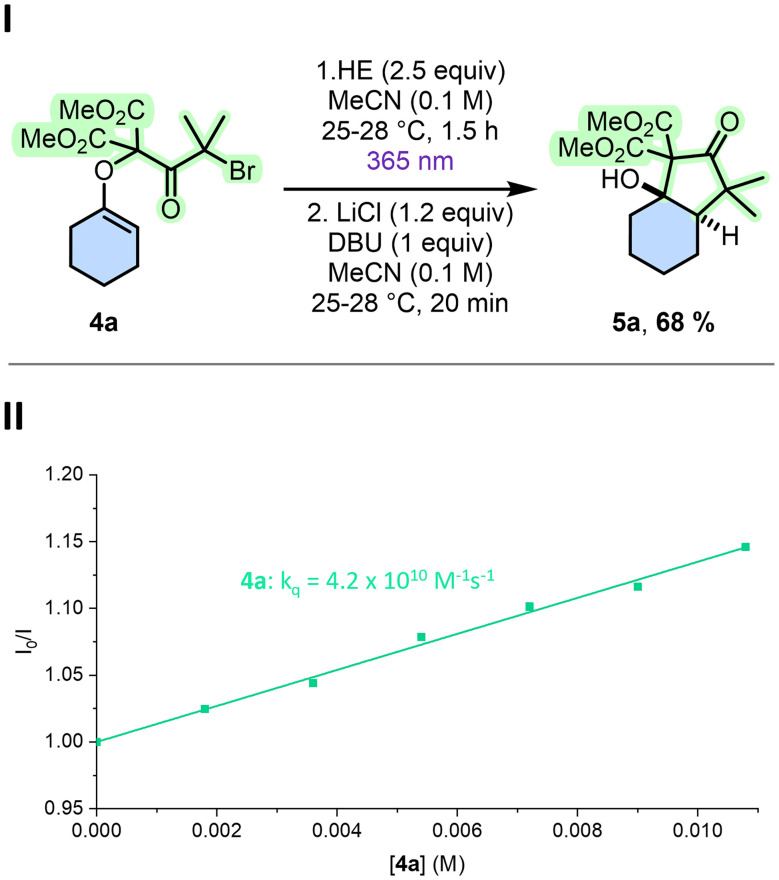
(I) Cyclization with HE under UV light (365 nm) followed by soft enolization/cyclization. Yield determined by ^1^H-NMR spectroscopy using 1,3,5-trimethoxybenzene as internal standard. (II) Stern–Volmer plot of the luminescence quenching of HE upon increasing concentration of 4a. Quenching experiments were conducted in dry acetonitrile under N_2_ atmosphere with HE (5 × 10^−5^ M), the emission intensity was followed at *λ* = 455 nm; *I*_0_ is the intensity before and *I* after adding the quencher 4a.

Finally, literature analysis indicated that HE can directly promote photoreductive processes, including selective debromination of α-bromo ketones.^40^ In this context, reactions were tested without photocatalyst under strict anaerobic conditions but employing a LED of higher energy. Satisfactorily, productive results were obtained while irradiating at 365 nm as product 5a was isolated in 68% yield (Fig. 2I).^41^ In fact, with enol ether 4a as substrate, HE (E_00_ = 3.06 eV and *E*^ox^ = +0.93 V *vs.* SCE) acts as photoreductant (HE* having E*^ox^ = −2.13 V). As it could be expected, Stern–Volmer analysis with gradual additions of 4a revealed an emission quenching of HE (*Φ* = 1.7% and *τ* = 0.32 ns in DMSO, of comparable polarity to acetonitrile). The SET process is characterized by a quenching constant of *k*_q_ = 4.2 × 10^10^ M^−1^ s^−1^ (Fig. 2II), corresponding to an efficiency *η* of 57% indicating a faster and more effective activation than the reactions derived from AOH^+^ as photocatalyst. In the present case, full reduction of 4a is enacted with 2.5 equivalents of HE in 1.5 hours only. Further investigation demonstrated a lack of photodecarbonylation after prolonged UV irradiation.^42^

## Conclusions

Highly functionalized *trans*-fused [*n*.3.0] bicycles (5) were synthesized (*n* = 4, 5, 10). The synthetic method combines photoinduced 6-*endo-trig* Giese additions with mild intramolecular aldol cyclizations. In practice, two distinct photoredox methods were considered. On one hand, photocatalytic conditions were developed using acridinium orange as a mild photocatalyst in the blue range (*λ*_exc_ = 455 nm), in combination with Hantzsch ester HE, which serves as both the reducing agent and hydrogen donor. On the other hand, HE was used for its own photoreductive properties at higher energy (*λ*_exc_ = 365 nm). Overall, these cyclization methods allow for the formation of unusually-dense *trans*-fused hydrindanones derivatives. While the process was mostly demonstrated for enol ethers made from cyclohexanone backbone, extension to other ring size remains a possibility. Finally, DFT calculations validate all the mechanistic pathways and regioselectivity in favor of a philicity matching of the electrophilic radical and the polar electron-rich nature of the malonate enol ether.

## Conflicts of interest

There are no conflicts to declare.

## Supplementary Material

QO-013-D5QO01635E-s001

QO-013-D5QO01635E-s002

## Data Availability

The data that support the findings of this study are openly available in yareta.unige.ch at https://doi.org/10.26037/yareta:ehkvr2be4za3bchnlyjv6pgeie. It will be preserved for 10 years. Supplementary information (SI) is available. See DOI: https://doi.org/10.1039/d5qo01635e. CCDC 2058766 and 2481639–2481641 contain the supplementary crystallographic data for this paper. These data can be be obtained free of charge from The Cambridge Crystallographic Data Centre *via*https://www.ccdc.cam.ac.uk/structures.^43*a*–*d*^
