# Supplementary material for: Densely-functionalized bicyclic cyclopentanones by combined photoinduced 6-endo-trig Giese additions and mild aldol cyclizations
Source: Org Chem Front. 2026 Jan 27;13(10):3021–9. doi: 10.1039/d5qo01635e (PMC12870345; doi:10.1039/d5qo01635e)
Supplement: QO-013-D5QO01635E-s001 [file QO-013-D5QO01635E-s001.pdf]

Supplementary Information (SI) for Organic Chemistry Frontiers.
This journal is © the Partner Organisations 2026

Densely-Functionalized Bicyclic Cyclopentanones by Combined Photoinduced 6-*endo-trig* Giese Additions and Mild Aldol Cyclizations

Júlia Viñas-Lóbez,[a] Nicolas Sellet,[a] Bibiana Fabri,[a] Guillaume Levitre,[a] Adiran de Aguirre,[a] Amalia I. Poblador-Bahamonde,[a] Céline Besnard,[b] and Jérôme Lacour [a]*

Supporting information

Table of Contents

1. General Remarks S2
2. Optimization of reaction conditions S6
3. Synthesis and data analysis of malonate enol ethers **2** S9
4. Synthesis and data analysis of acylated malonate enol ethers **4** S12
5. Synthesis and data analysis of *trans*-fused bicycles **5**, **7i** and **9a** S17
6. NMR spectra S24
7. Photophysical and Electrochemical data S57
8. Quenching experiments S62
9. Mechanistic studies S67
   9.1. Path *ii* S67
   9.2 Fused vs spiro cycle formation – Comparison **A**, **A1**, **A2** and **A3** S68
   9.3 Temptative concerted formation of **5a** S68
   9.4 Computational benchmarking S69
   9.5 Diastereoselective formation of **5j** and **5k** S70
10. Crystallographic data S71
11. References S79

1. General Remarks

Unless otherwise stated, reagents were purchased from commercial sources and used without further purification. Dry CH_3CN, CH_2Cl_2 and THF were used without any purification (stored under N_2 for photocatalyzed process). NMR spectra were recorded on 400 or 500 MHz spectrometer at room temperature. ^{1}H-NMR: chemical shifts are given in ppm relative to Me_4Si with solvent resonances used as internal standards ($CDCl_3$ δ = 7.26 ppm or acetone-d_6 δ = 2.05 ppm). Data were reported as follows: chemical shift (δ) in ppm on the δ scale, multiplicity (s = singlet, d = doublet, t = triplet, dd = doublet of doublets, dt = doublet of triplets and m = multiplet), coupling constant (Hz) and integration. ^{13}C-NMR chemicals shifts were given in ppm relative to Me_4Si with solvent resonances used as internal standards ($CDCl_3$ δ = 77.16 ppm or acetone-d_6 δ = 29.84 and 206.26 ppm). IR spectra were recorded using an ATR sampler and are reported in wave numbers (cm^{-1}). Electrospray mass spectra (ESI) were obtained by the department of Mass Spectrometry of the University of Geneva. Flash column chromatography was performed with silica gel 40-63 μm.

Important note: Diazo compounds are high energy materials and should be handled with caution. Although α-diazomalonates are stable at room temperature, it is advisable to carry out reactions behind a blast shield.[1]

Irradiation sources used during the study:

ThorLabs mounted LED 455 nm, light-emitting diode (LED, nominal wavelength: 455 nm, output power: 900 mW, irradiance: 31.2 μW/mm^2).

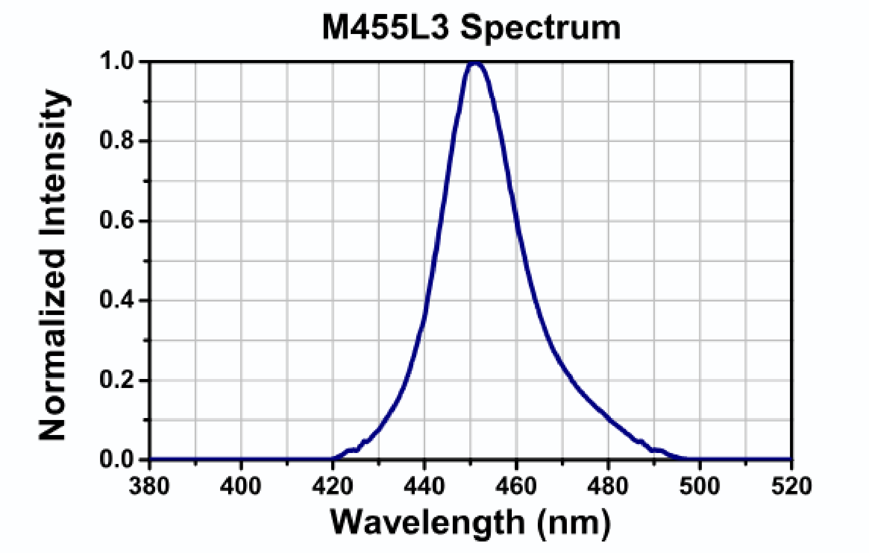

Emission spectra of **M455L3** (Thorlabs LED)

A commercially available blue LED strip (λ_{max} = 460 nm) was wrapped around a crystallizer and covered with aluminum foil to create the photoreactor. To cool the reaction setup, an airflow was introduced through an opening.

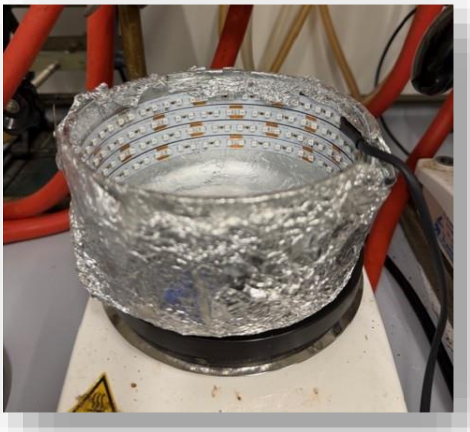
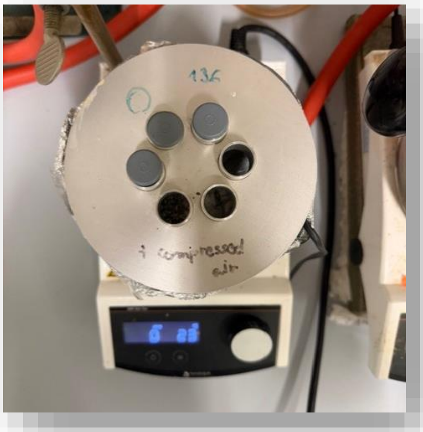

Peschl Ultraviolet advanced UV-products: power supply **TQ150** with radiation source **TQ150 Z0**. [2]

TQ 150, undoped
Radiation flux Φ 200 - 600nm: 47W

λ nm	TQ 150 lamp		operated in DURAN 50 sleeve	
	Radiation flux Φ (W)	Mole quanta/h x 10^{-3}	Radiation flux Φ (W)	Mole quanta/h x 10^{-3}
238/40	1	8	-	-
248	0,7	5	-	-
254	4	30	-	-
265	1,4	11	-	-
270	0,6	5	-	-
275	0,3	2	-	-
280	0,7	6	-	-
289	0,5	4	-	-
297	1	9	0,1	1
302	1,8	17	0,5	4
313	4,3	41	2,5	23
334	0,5	5	0,4	4
366	6,4	71	5,8	64
390	0,1	1	0,1	1
405/08	3,2	39	2,9	35
436	4,2	55	3,6	50
492	0,1	1	0,1	1
546	5,1	84	4,6	76
577/79	4,7	82	4,2	74

Energy distribution of **TQ150 Z0** radiation source

Optical properties: All optical measurements were performed at room temperature in 1 cm optical path quartz cells. Air-equilibrated analytical grade acetonitrile solutions were used, with the exception of Stern-Volmer quenching experiments that were carried out in analytical grade extra dry acetonitrile (AcroSeal®) under inert atmosphere (N_2), using UHV stopcock quartz cuvettes.

UV-Vis absorption spectra were recorded on a JASCO V-650 spectrophotometer.

Steady-state fluorescence spectra were measured using a FluoroMax+ spectrofluorometer from Horiba Scientific. All fluorescence spectra were corrected for the wavelength-dependent sensitivity of the detection. Fluorescence quantum yields Φ were determined by comparison with a standard of known quantum yield using the following equation:

$$\Phi=\Phi_r \frac{I\ A_r\ n^2}{I_r\ A\ n_r^2}$$

where A is the absorbance at the excitation wavelength (λ), n the refractive index and I the integrated emission intensity; "r" stands for reference. Diluted solutions with absorption lower than 0.1 were employed. Excitations of reference and sample compounds were performed at the same wavelength.

Fluorescence lifetimes on the nanosecond timescale were measured by a time-correlated single photon counting (TCSPC) setup. Excitation was performed at 400 nm using ~60 ps pulse at 20 MHz produced by a laser diode (PicoQuant, LDH-P-C-400). The fluorescence decay of **AOH$^+$** was followed at wavelength 516 nm using band-pass filters. The full width at half-maximum (fwhm) of the instrument response function (IRF) was around 200 ps. The fluorescence time profiles were analyzed with the deconvolution of the experimental IRF and an exponential function.

Cyclic voltammetry (CV) experiments were performed with a CH instrument potentiostat. Measurements were conducted at room temperature under nitrogen in a one-compartment, three-electrode cell that was pre-dried. The Schlenk-type construction of the cell maintained anhydrous and anaerobic conditions. Measurements were performed in analytical grade extra dry acetonitrile (AcroSeal®) with [nBu$_4$N][PF$_6$] as supporting electrolyte (10^{-1} M). A Pt disk (Φ = 3 mm) was employed as working electrode (WE), a Pt wire as counter electrode (CE) and an Ag/AgNO$_3$ (10^{-2} M + [nBu$_4$N][PF$_6$] 10^{-1} M) electrode was used as a pseudo-reference (RE) during the measurements. The solution of supporting electrolyte was introduced into the cell and the CV of the solvent was recorded. The analyte was then added and voltammograms were performed (scan rate, ν = 0.1 V/s); then a small amount of ferrocene (Cp_2Fe, Fc) was

added and the voltammograms repeated. Potentials were corrected by placing $E_{1/2}$ for the Cp_2Fe^+/Cp_2Fe couple equal to +0.0 V and then converted to SCE using $E_{1/2}$ = +0.4 V vs SCE.[3]

Computational details

All calculations were carried out using Gaussian16 package.[4] The B3LYP functional including Grimme dispersion D3 term was chosen as level of theory.[5] All stationary points were assigned to a minima (zero imaginary frequencies) or transition state (one imaginary frequency) by frequency analysis. Optimizations and frequency calculations for radicals species were ran using the basis set 6-31+G* [6] for all atoms while neutral species were ran using 6-311++G** as basis set for all atoms. In both cases, calculations were carried out in dichloromethane solution (ε = 8.93), using the SMD model.[7] All the reported energies are Gibbs free energies in solution calculated at 298 K and 1 atm in kcal/mol. A benchmark for the computed reaction selectivity was carried out, and it is detailed in Section 9 of this supporting information.

2. Optimization of reaction conditions

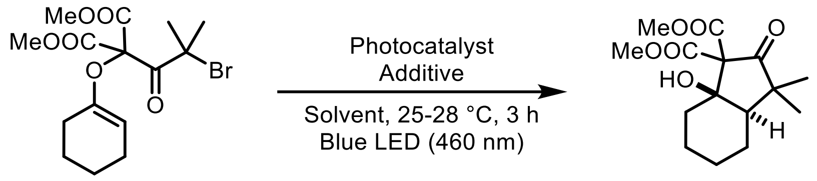

4a 0.05 mmol → **5a**

Entry	Catalyst	Cat. Loading	Additive	Solvent	[M]	Yield
1	PC-1	2.5 mol%	DIPEA (10 equiv)	MeCN	0.1	48
2	PC-1	2.5 mol%	Pyridine (10 equiv)	MeCN	0.1	25
3	PC-1	2.5 mol%	K_2CO_3 (10 equiv)	MeCN	0.1	n.r.
4	PC-1	2.5 mol%	DBU (10 equiv)	MeCN	0.1	n.d.
5	PC-1	2.5 mol%	Lutidine (10 equiv)	MeCN	0.1	28
6	PC-1	2.5 mol%	Et_3N (10 equiv)	MeCN	0.1	37
7	PC-1	2.5 mol%	DIPEA (10 equiv)	THF	0.1	32
8	PC-1	2.5 mol%	DIPEA (10 equiv)	DMF	0.1	32
9	PC-1	2.5 mol%	DIPEA (10 equiv)	Toluene	0.1	30
10	PC-1	2.5 mol%	DIPEA (10 equiv)	Dioxane	0.1	35
11	PC-1	1.0 mol%	DIPEA (10 equiv)	MeCN	0.1	43
12	PC-1	5.0 mol%	DIPEA (10 equiv)	MeCN	0.1	41
13	PC-2	2.5 mol%	DIPEA (10 equiv)	MeCN	0.1	35
14	PC-3	2.5 mol%	DIPEA (10 equiv)	MeCN	0.1	50
15	PC-4	2.5 mol%	DIPEA (10 equiv)	MeCN	0.1	45
16	PC-3	2.5 mol%	DIPEA (10 equiv), ^{t}BuOH (5 equiv)	MeCN	0.1	54
17	PC-3	2.5 mol%	DIPEA (10 equiv), **HE**-1 (5 equiv)	MeCN	0.1	60
18	PC-3	2.5 mol%	DIPEA (10 equiv), **HE**-1 (10 equiv)	MeCN	0.1	52
19	PC-3	2.5 mol%	DIPEA (10 equiv), **HE**-1 (2.5 equiv)	MeCN	0.1	56
20	PC-3	2.5 mol%	**HE**-1 (10 equiv)	MeCN	0.1	72
21	PC-3	2.5 mol%	**HE**-1 (5 equiv)	MeCN	0.1	66
22	PC-3	2.5 mol%	**HE**-1 (2.5 equiv)	MeCN	0.1	63

23	PC-1	2.5 mol%	**HE**-1 (10 equiv)	MeCN	0.1	63
24	PC-1	2.5 mol%	**HE**-1 (5 equiv)	MeCN	0.1	63
25	PC-1	2.5 mol%	**HE**-1 (2.5 equiv)	MeCN	0.1	70
26	PC-1	2.5 mol%	**HE**-1 (1.5 equiv)	MeCN	0.1	59
27	PC-5	2.5 mol%	**HE**-1 (2.5 equiv)	MeCN	0.1	60
28	PC-6	2.5 mol%	**HE**-1 (2.5 equiv)	MeCN	0.1	68
29	PC-7	2.5 mol%	**HE**-1 (2.5 equiv)	MeCN	0.1	62
30	PC-8	2.5 mol%	**HE**-1 (2.5 equiv)	MeCN	0.1	24
31	PC-9	2.5 mol%	**HE**-1 (2.5 equiv)	MeCN	0.1	74
32	PC-10	2.5 mol%	**HE**-1 (2.5 equiv)	MeCN	0.1	66
33	PC-11	2.5 mol%	**HE**-1 (2.5 equiv)	MeCN	0.1	66
34	PC-12	2.5 mol%	**HE**-1 (2.5 equiv)	MeCN	0.1	47
35	PC-13	2.5 mol%	**HE**-1 (2.5 equiv)	MeCN	0.1	31
36	PC-14	2.5 mol%	**HE**-1 (2.5 equiv)	MeCN	0.1	51
37	PC-15	2.5 mol%	**HE**-1 (2.5 equiv)	MeCN	0.1	24
38	PC-16	2.5 mol%	**HE**-1 (2.5 equiv)	MeCN	0.1	61
39	PC-9	2.5 mol%	**HE**-2 (2.5 equiv)	MeCN	0.1	76
40	PC-9	2.5 mol%	**HE**-3 (2.5 equiv)	MeCN	0.1	49
41	PC-9	2.5 mol%	**HE**-4 (2.5 equiv)	MeCN	0.1	64
42	PC-9	2.5 mol%	**HE**-1 (1.5 equiv)	MeCN	0.1	65
43	PC-9	2.5 mol%	**HE**-1 (3 equiv)	MeCN	0.1	70
44	PC-9	2.5 mol%	**HE**-1 (2.5 equiv)	^{t}BuCN	0.1	64
45	PC-9	2.5 mol%	**HE**-1 (2.5 equiv)	THF	0.1	68
46	PC-9	2.5 mol%	**HE**-1 (2.5 equiv)	Toluene	0.1	48
47	PC-9	2.5 mol%	**HE**-1 (2.5 equiv)	Dioxane	0.1	70
48	PC-9	2.5 mol%	**HE**-1 (2.5 equiv)	Et_2O	0.1	56
49	PC-9	2.5 mol%	**HE**-1 (2.5 equiv)	EtOAc	0.1	68
50	PC-9	2.5 mol%	**HE**-1 (2.5 equiv)	DMF	0.1	63
51	PC-9	2.5 mol%	**HE**-1 (2.5 equiv)	DMA	0.1	72
52	PC-9	2.5 mol%	**HE**-1 (2.5 equiv)	MeOH	0.1	51
53	PC-9	2.5 mol%	**HE**-1 (2.5 equiv)	EtOH	0.1	45
54	PC-9	2.5 mol%	**HE**-1 (2.5 equiv)	iPrOH	0.1	54
55	PC-9	1 mol%	**HE**-1 (2.5 equiv)	MeCN	0.1	73
56	PC-9	5 mol%	**HE**-1 (2.5 equiv)	MeCN	0.1	72

57	PC-9	2.5 mol%	**HE**-1 (2.5 equiv)	MeCN	0.01	68
58	PC-9	2.5 mol%	**HE**-1 (2.5 equiv)	MeCN	0.05	71
59	PC-9	2.5 mol%	**HE**-1 (2.5 equiv)	MeCN	0.2	64

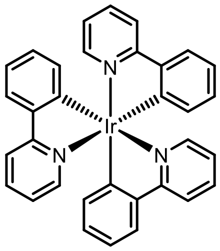

***fac*-Ir(ppy)$_3$ (PC-1)**

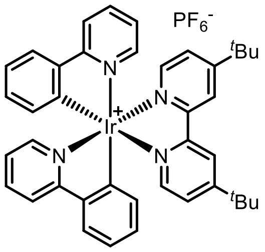

[Ir(dtbbpy)(ppy)$_2$]PF$_6$ (PC-2)

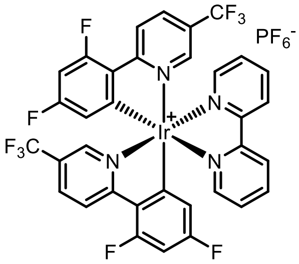

[Ir{dFCF$_3$ppy}$_2$(bpy)]PF$_6$ (PC-3)

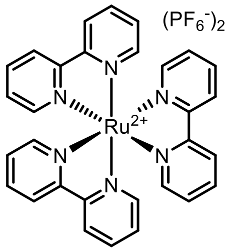

Ru(bpy)$_3$(PF$_6$)$_2$ (PC-4)

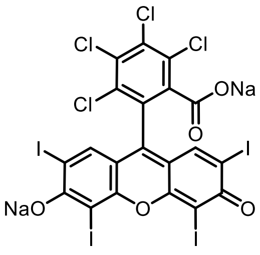

Rose Bengal (PC-5)

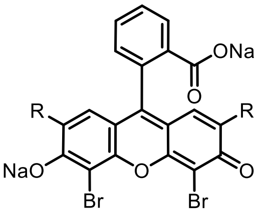

Eosin Y (PC-6): R= Br

Eosin B (PC-7): R= NO_2

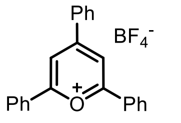

TPT$^+$ (PC-8)

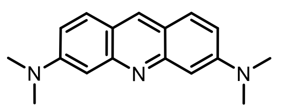

Acridine Orange (PC-9)

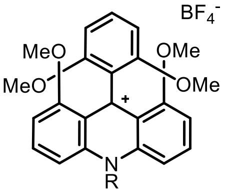

R= Me, Pr, Und

TEMA$^+$ (PC-10, PC-11, PC-12)

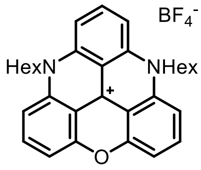

DAOTA$^+$ (PC-13)

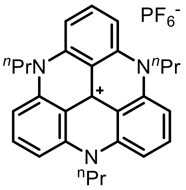

TATA $^+$ (PC-14)

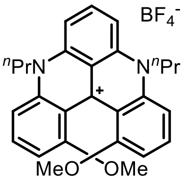

DMQA$^+$ (PC-15)

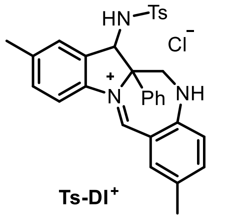

Ts-DI$^+$ (PC-16)

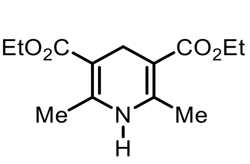

HE-1

72%^{a}

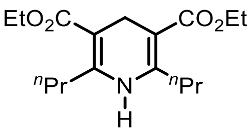

HE-2

76%

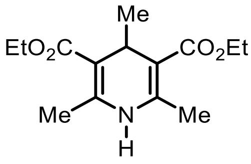

HE-3

49%

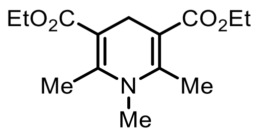

HE-4

64%

Table S1. *Optimization of reaction conditions.*

3. Synthesis and data analysis of malonate enol ethers 2

Compounds **2** which are not reported below, were synthesized following previous reported procedure.[8]

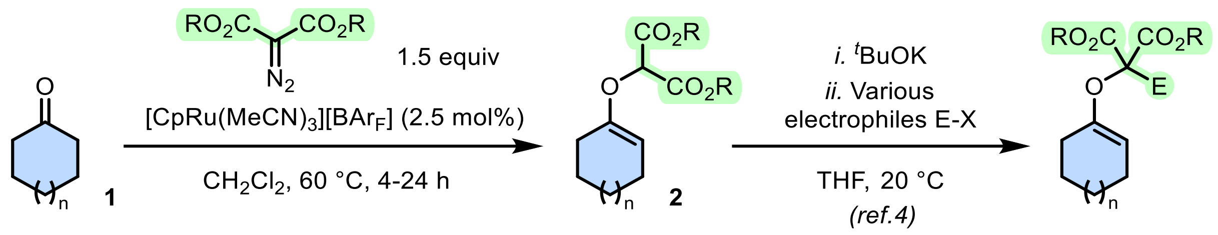

Scheme S1. CpRu-catalyzed formation of malonate enol ethers **2** and subsequent functionalization by malonate activation. See reference [8], end of document.

General procedure A

In a 2 mL screw-cap vial equipped with a magnetic stirring bar, $[CpRu(CH_3CN)_3][BAr_F]$ (7.5 µmol, 2.5 mol%) was dissolved in 0.60 mL of dry dichloromethane. Cyclic ketone **1** (0.3 mmol) was added followed by diazo malonate (0.45 mmol, 1.5 equiv). The vial was capped, and the solution was stirred at 60 °C until full conversion (^{1}H NMR and TLC monitoring). The solution was concentrated under reduced pressure, and the residue was purified by column chromatography (pentane/EtOAc, SiO_2) to afford malonate enol ethers of type **2**.

Dimethyl 2-((4,4-difluorocyclohex-1-en-1-yl)oxy)malonate (**2e**)

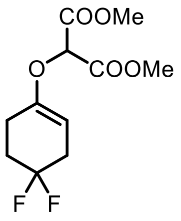

Following general procedure A and after 16 h, **2e** was obtained from methyl diazomalonate (0.36 mL, 3 mmol) and corresponding ketone (268 mg, 2 mmol) as a colorless oil (486 mg, 92% yield). Purification: Column chromatography (pentane/EtOAc 9:1).

Rf = 0.58 (SiO_2, pentane/EtOAc, 8:2); **^{1}H NMR (400 MHz, acetone-*d6*):** δ 5.13 (s, 1H), 4.58 (s, 1H), 3.77 (s, 6H), 2.53 (t, J = 14.3 Hz, 2H), 2.39 (t, J = 6.5 Hz, 2H), 2.13 (tt, J = 13.9, 6.7 Hz, 2H); **^{13}C NMR (100 MHz, acetone-*d6*):** δ 166.5, 152.7, 123.5 (t, J = 238.9 Hz), 92.8 (t, J = 6.3 Hz), 76.1, 53.2, 33.1 (t, J = 27.3 Hz), 30.5 (t, J = 25.3 Hz), 26.0 (t , J = 5.9 Hz); **^{19}F NMR (282 MHz, acetone-*d6*):** δ -98.64; **IR (neat):** 2960, 1746, 1676, 1437, 1178, 1068, 882 cm^{-1}; **HRMS (ESI):** Calculated for $C_{11}H_{14}F_2O_5$ $[M+Na]^+$: 287.0702; Found: 287.0687 m/z.

Dimethyl 2-((1,4-dioxaspiro[4.5]dec-7-en-8-yl)oxy)malonate (**2f**)

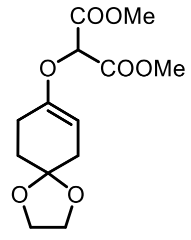

Following general procedure A and after 16 h, **2f** was obtained from methyl diazomalonate (0.36 mL, 3 mmol) and corresponding ketone (312 mg, 2 mmol) as a colorless oil (509 mg, 89% yield). Purification: Column chromatography (pentane/EtOAc 8:2).

Rf = 0.15 (SiO_2, pentane/EtOAc, 7:3); **^{1}H NMR (400 MHz, acetone-*d₆*):** δ 5.05 (s, 1H), 4.52 (t, J = 4.3 Hz, 1H), 3.91 (s, 4H), 3.77 (s, 6H), 2.34 – 2.24 (m, 2H), 2.22 – 2.15 (m, 2H), 1.76 (t, J = 6.6 Hz, 2H); **^{13}C NMR (101 MHz, acetone-d_6):** δ 166.8, 153.4, 107.9, 94.8, 76.0, 64.9, 53.1, 34.2, 31.5, 27.0; **IR (neat):** 2957, 1771, 1744, 1675, 1436, 1171, 1120, 1058, 1014, 856, 730 cm^{-1}; **HRMS (ESI):** Calculated for $C_{13}H_{18}O_7$ $[M+Na]^+$: 309.0945; Found: 309.0943 m/z.

Diethyl 4-((1,3-dimethoxy-1,3-dioxopropan-2-yl)oxy)cyclohex-3-ene-1,1-dicarboxylate (**2g**)

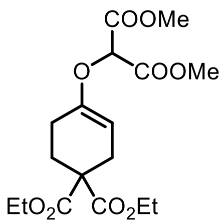

Following general procedure A and after 16 h, **2g** was obtained from methyl diazomalonate (0.36 mL, 3 mmol) and corresponding ketone (485 mg, 2 mmol) as a colorless oil (616 mg, 83% yield). Purification: Column chromatography (pentane/EtOAc 8:2).

Rf = 0.26 (SiO_2, pentane/EtOAc, 8:2); **^{1}H NMR (400 MHz, acetone-d_6):** δ 5.09 (s, 1H), 4.65 (t, J = 4.4 Hz, 1H), 4.15 (qd, J = 7.1, 1.5 Hz, 4H), 3.76 (s, 6H), 2.60 – 2.48 (m, 2H), 2.25 – 2.19 (m, 2H), 2.18 – 2.13 (m, 2H), 1.21 (t, J = 7.1 Hz, 6H).; **^{13}C NMR (101 MHz, acetone-d_6):** δ 171.4, 166.7, 152.9, 95.0, 75.8, 61.9, 53.6, 53.1, 29.6, 28.3, 25.2, 14.3; **IR (neat):** 2958, 1728, 1679, 1437, 1249, 1166, 1020, 860, 789 cm^{-1}; **HRMS (ESI):** Calculated for $C_{17}H_{24}O_9$ $[M+Na]^+$: 395.1313; Found: 395.1304 m/z.

dimethyl 2-((4,4-dimethylcyclohex-1-en-1-yl)oxy)malonate (**2h**)

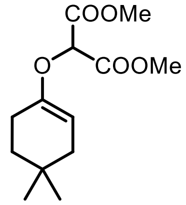

Following general procedure A and after 4 h, **2h** was obtained from methyl diazomalonate (0.36 mL, 3 mmol) and corresponding ketone (0.25 mL, 2 mmol) as a colorless oil (355 mg, 69 % yield). Purification: Column chromatography (pentane/EtOAc 95:5).

^{1}H NMR (400 MHz, acetone-d_6): δ 5.09 (s, 1H), 4.55 (t, *J* = 4.0 Hz, 1H), 3.76 (s, 6H), 2.12 (ttd, *J* = 6.6, 2.1, 1.2 Hz, 2H), 1.81 (dt, *J* = 4, 2.1 Hz, 2H), 1.43 (t, *J* = 6.6 Hz, 2H), 0.91 (s, 6H); **^{13}C NMR (101 MHz, acetone-d_6):** δ 166.1, 152.12, 95.2, 74.8, 52.2, 37.0, 35.1, 28.5, 27.1, 24.8; **IR (neat):** 2956, 1744, 1674, 1437, 1192, 1156, 1020, 799 cm^{-1}; **HRMS (ESI):** Calculated for $C_{13}H_{20}O_5$ $[M+Na]^+$:279.1203; Found: 279.1210 m/z.

dimethyl 2-((3,3,5,5-tetramethylcyclohex-1-en-1-yl)oxy)malonate (**2i**)

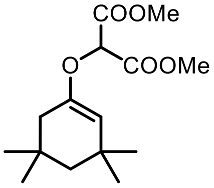

Following general procedure A and after 4 h, **2i** was obtained from methyl diazomalonate (0.36 mL, 3 mmol) and corresponding ketone (0.25 mL, 2 mmol) as a colorless oil (310 mg, 64% yield). Purification: Column chromatography (pentane/EtOAc 95:5)

^{1}H NMR (400 MHz, acetone-*d$_6$*): δ 5.04 (s, 1H), 4.62 – 4.55 (m, 1H), 3.75 (2x s, 6H), 2.26 – 2.11 (m, 1H), 2.12 – 2.01 (m, 2H), 1.78 – 1.67 (m, 1H), 1.67 – 1.52 (m, 2H), 1.35 – 1.20 (m, 1H), 0.93 (d, J = 6.3 Hz, 3H); **^{13}C NMR (101 MHz, acetone-*d$_6$*):** δ 167.0, 151.6, 107.0, 75.7, 53.0, 32.1, 30.1; **IR (neat):** 2952, 2903, 1771, 1761, 1739, 1671, 1447, 1225, 1154, 1141, 822 cm^{-1}; **HRMS (ESI):** Calculated for $C_{15}H_{24}O_5$ $[M+Na]^+$: 307.1516 ; Found: 307.1516 m/z.

Dimethyl 2-((4-methylcyclohex-1-en-1-yl)oxy)malonate (**2k**)

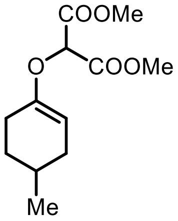

Following general procedure A and after 4 h, **2k** was obtained from methyl diazomalonate (0.36 mL, 3 mmol) and corresponding ketone (0.25 mL, 2 mmol) as a colorless oil (310 mg, 64% yield). Purification: Column chromatography (pentane/EtOAc 95:5).

^{1}H NMR (400 MHz, acetone-*d$_6$*): δ 5.04 (s, 1H), 4.62 – 4.55 (m, 1H), 3.75 (2x s, 6H), 2.26 – 2.11 (m, 1H), 2.12 – 2.01 (m, 2H), 1.78 – 1.67 (m, 1H), 1.67 – 1.52 (m, 2H), 1.35 – 1.20 (m, 1H), 0.93 (d, J = 6.3 Hz, 3H); **^{13}C NMR (101 MHz, acetone-*d$_6$*):** δ 166.1, 166.0, 153.1, 95.6, 74.8, 52.2, 31.5, 30.6, 28.3, 27.0, 20.5; **IR (neat):** 2955, 2927, 1773, 1746, 1672, 1436, 1172, 1154, 1021, 790 cm^{-1}; **HRMS (ESI):** Calculated for $C_{12}H_{18}O_5$ $[M+Na]^+$: 265.1047; Found: 265.1036 m/z.

4. Synthesis and data analysis of acylated malonate enol ethers 4

General procedure B

In a 25 mL flask equipped with a magnetic stirring bar, malonate enol ether **2** (1 equiv) was dissolved in dry THF (0.1 M). KHMDS (2 equiv) was added and the solution was stirred at room temperature for 5 min, while a yellow-orange precipitate was formed. α-bromoisobutyryl bromide was added (2 equiv) and the mixture was stirred at 25 °C until full conversion of **2** (^{1}H NMR and TLC monitoring). Water was then added and the mixture was extracted twice with EtOAc. The organic layer was dried over Na_2SO_4, filtered and concentrated under reduced pressure. The residue was purified by column chromatography (pentane/EtOAc, SiO_2) to afford **4**.

Dimethyl 2-(2-bromo-2-methylpropanoyl)-2-(cyclohex-1-en-1-yloxy)malonate (**4a**)

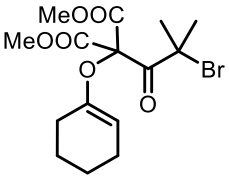

Following general procedure B and after 30 min, **4a** was obtained from enol ether **2a** (114 mg, 0.5 mmol) as a colorless oil (120 mg, 64% yield). Purification: Column chromatography (pentane/EtOAc 95:5).

Rf = 0.52 (SiO_2, pentane/EtOAc, 9:1); **^{1}H NMR (400 MHz, Acetone-*d₆*):** δ 5.07 (tt, J = 4.0, 1.4 Hz, 1H), 3.80 (s, 6H), 2.16 (tq, J = 6.2, 2.3 Hz, 2H), 2.01 (dtt, J = 6.3, 4.4, 2.1 Hz, 2H), 1.96 (s, 6H), 1.73 – 1.62 (m, 2H), 1.56 – 1.45 (m, 2H); **^{13}C NMR (126 MHz, Acetone-*d₆*):** δ 196.0, 165.9, 153.0, 104.3, 92.9, 61.7, 53.6, 31.4, 28.8, 24.2, 23.5, 22.7; **IR (neat):** 2933, 2844, 1817, 1752, 1719, 1677, 1435, 1248, 1154, 1046, 803 cm^{-1}; **HRMS (ESI):** Calculated for $C_{15}H_{21}BrO_6$ $[M+Na]^+$: 399.0414; Found: 399.0399 m/z.

Dibenzyl 2-(2-bromo-2-methylpropanoyl)-2-(cyclohex-1-en-1-yloxy)malonate (**4b**)

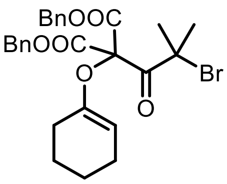

Following general procedure B and after 30 min, **4b** was obtained from enol ether **2b** (325 mg, 0.85 mmol) as a colorless oil (266 mg, 59 % yield). Purification: Column chromatography (pentane/EtOAc 97:3).

^{1}H NMR (400 MHz, Acetone-*d₆*): δ 7.44 – 7.31 (m, 10H), 5.31 (d, J = 12.3 Hz, 2H), 5.24 (d, J = 12.3 Hz, 2H), 4.97 (tt, J = 4.0, 1.3 Hz, 1H), 2.13 – 2.07 (m, 2H), 1.88 (s, 6H), 1.89 – 1.78 (m, 2H), 1.61 – 1.53 (m, 2H), 1.40 – 1.32 (m, 2H); **^{13}C NMR (126 MHz, Acetone-*d₆*):** δ 196.0, 165.3, 152.6, 136.1, 129.5, 129.5, 129.4, 104.1, 68.9, 61.9, 31.4, 28.8, 24.1, 23.5, 22.7; **IR (neat):** 3066, 3034, 2929, 1748, 1718, 1677, 1456, 1158, 1040, 752, 696 cm^{-1} **HRMS (ESI):** Calculated for $C_{27}H_{29}BrO_6$ $[M+Na]^+$: 551.1040; Found: 551.1031 m/z.

Dimethyl 2-(2-bromo-2-methylpropanoyl)-2-(cyclohept-1-en-1-yloxy)malonate (**4c**)

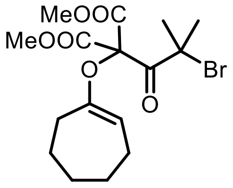

Following general procedure B and after 20 min, **4c** was obtained from enol ether **2c** (121 mg, 0.5 mmol) as a colorless oil (186 mg, 95% yield). Purification: Column chromatography (pentane/EtOAc 95:5). **^{1}H NMR (400 MHz, Acetone-*d$_6$*):** δ 5.13 (t, J = 6.7 Hz, 1H), 3.80 (s, 6H), 2.48 – 2.36 (m, 2H), 2.05 – 1.99 (m, 2H), 1.96 (s, 6H), 1.77 – 1.65 (m, 2H), 1.64 – 1.55 (m, 2H), 1.55 – 1.44 (m, 2H); **^{13}C NMR (101 MHz, Acetone-*d$_6$*):** δ 195.7, 165.8, 158.6, 107.0, 92.8, 61.5, 53.6, 34.2, 32.1, 31.3, 28.1, 25.6, 25.4; **HRMS (ESI):** Calculated for $C_{16}H_{23}BrO_6$ [M+Na]$^+$: 413.0571; Found: 413.0557 m/z.

Dimethyl 2-(2-bromo-2-methylpropanoyl)-2-(cyclopent-1-en-1-yloxy)malonate (**4d**)

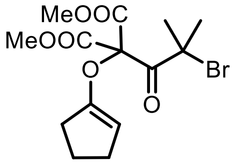

Following general procedure B and after 30 min, **4d** was obtained from enol ether **2d** (107 mg, 0.5 mmol) as a yellowish oil (122 mg, 67 % yield). Purification: Column chromatography (pentane/EtOAc 95:5).

Rf = 0.59 (SiO_2, pentane/EtOAc, 9:1); **^{1}H NMR (400 MHz, Acetone-*d$_6$*):** δ 4.71 – 4.65 (m, 1H), 3.81 (s, 6H), 2.47 – 2.37 (m, 2H), 2.33 – 2.23 (m, 2H), 1.96 (s, 6H), 1.86 (p, J = 7.4 Hz, 2H); **^{13}C NMR (101 MHz, Acetone-*d$_6$*):** δ 195.7, 165.5, 155.7, 103.0, 92.7, 61.8, 53.7, 32.8, 31.3, 29.7, 21.6; **IR (neat):** 2955, 2854, 1750, 1719, 1653, 1435, 1226, 1108, 1050, 971, 736 cm^{-1}; **HRMS (ESI):** Calculated for $C_{14}H_{19}BrO_6$ [M+Na]$^+$: 385.0258; Found: 385.0239 m/z.

Dimethyl 2-(2-bromo-2-methylpropanoyl)-2-((4,4-difluorocyclohex-1-en-1-yl)oxy) malonate (**4e**)

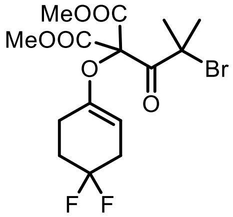

Following general procedure B and after 30 min, **4e** was obtained from enol ether **2e** (264 mg, 1 mmol) as a white solid (291 mg, 70% yield). Purification: Column chromatography (pentane/EtOAc 95:5).

Rf = 0.52 (SiO_2, pentane/EtOAc, 9:1); **^{1}H NMR (400 MHz, Acetone-*d$_6$*):** δ 5.06 – 4.98 (m, 1H), 3.81 (s, 6H), 2.55 (t, J = 14.3 Hz, 2H), 2.51 – 2.42 (m, 2H), 2.15 (tt, J = 13.6, 6.8 Hz, 2H), 1.96 (s, 6H); **^{13}C NMR (101 MHz, Acetone-*d$_6$*):** δ 195.6 , 165.7 , 151.8 , 123.3 (d, J = 238.9 Hz), 100.1 (t, J = 6.4 Hz), 93.2, 61.4, 53.8, 33.3 (t, J = 27.7 Hz), 31.2, 30.7 (t, J = 25.1 Hz), 26.9 (t, J = 5.6 Hz); **^{19}F NMR (282 MHz, acetone-*d$_6$*):** δ -98.83; **IR (neat):** 2957, 1752, 1719, 1681, 1436, 1385, 1249, 972, 886, 803, 685 cm^{-1}; **HRMS (ESI):** Calculated for $C_{15}H_{19}BrF_2O_6$ [M+Na]$^+$: 435.0226; Found: 435.0234 m/z; **M.p.:** 37-40 °C.

Dimethyl 2-((1,4-dioxaspiro[4.5]dec-7-en-8-yl)oxy)-2-(2-bromo-2-methylpropanoyl) malonate (**4f**)

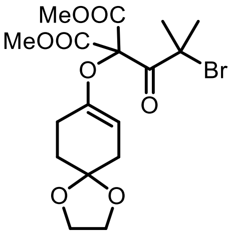

Following general procedure B and after 30 min, **4f** was obtained from enol ether **2f** (286 mg, 1 mmol) as a white solid (334 mg, 77% yield). Purification: Column chromatography (pentane/EtOAc 8:2).

Rf = 0.29 (SiO_2, pentane/EtOAc, 8:2); **^{1}H NMR (400 MHz, Acetone-*d_6*):** δ 5.01 (tt, J = 4.0, 1.2 Hz, 1H), 3.93 (s, 4H), 3.80 (s, 6H), 2.36 (ttd, J = 6.6, 2.1, 1.2 Hz, 2H), 2.23 – 2.16 (m, 2H), 1.96 (s, 6H), 1.78 (t, J = 6.6 Hz, 2H).; **^{13}C NMR (101 MHz, Acetone-*d_6*):** δ 195.9, 166.0, 152.5, 107.7, 102.6, 93.4, 64.9, 61.6, 53.7, 34.5, 31.6, 31.3, 27.9; **IR (neat):** 2956, 2940, 1749, 1717, 1683, 1432, 1257, 1157, 1099, 1048, 949, 869 cm^{-1}; **HRMS (ESI):** Calculated for $C_{17}H_{23}BrO_8$ $[M+Na]^+$: 457.0469; Found: 457.0479 m/z; **M.p.:** 75-79 °C.

Diethyl 4-((4-bromo-1-methoxy-2-(methoxycarbonyl)-4-methyl-1,3-dioxopentan-2-yl)oxy)cyclohex-3-ene-1,1-dicarboxylate (**4g**)

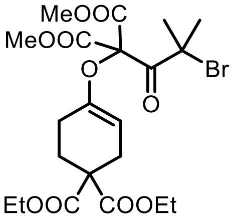

Following general procedure B and after 30 min, **4g** was obtained from enol ether **2g** (372 mg, 1 mmol) as a colorless oil (272 mg, 52% yield). Purification: Column chromatography (pentane/EtOAc 9:1).

Rf = 0.48 (SiO_2, pentane/EtOAc, 8:2); **^{1}H NMR (400 MHz, Acetone-*d_6*):** δ 5.05 (tt, J = 4.0, 1.4 Hz, 1H), 4.18 (qd, J = 7.1, 5.5 Hz, 4H), 3.80 (s, 6H), 2.60 – 2.54 (m, 2H), 2.31 – 2.24 (m, 2H), 2.21 – 2.14 (m, 2H), 1.95 (s, 6H), 1.23 (t, J = 7.1 Hz, 6H).; **^{13}C NMR (101 MHz, Acetone-*d_6*):** δ 194.8, 170.4, 164.9, 150.7, 100.6, 92.0, 61.0, 60.6, 52.9, 30.4, 28.9, 27.5, 25.0, 13.4; **IR (neat):** 2958, 1721, 1685, 1436, 1248, 1161, 1095, 1047, 963, 817 cm^{-1}; **HRMS (ESI):** Calculated for $C_{21}H_{29}BrO_{10}$ $[M+Na]^+$: 543.0837; Found: 543.0846 m/z.

Dimethyl 2-(2-bromo-2-methylpropanoyl)-2-((4,4-dimethylcyclohex-1-en-1-yl)oxy) malonate (**4h**)

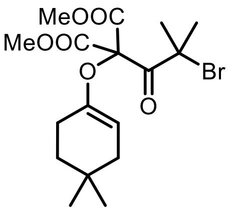

Following general procedure B and after 30 min, **4h** was obtained from enol ether **2h** (128 mg, 0.5 mmol) as a colorless oil (158 mg, 78% yield). Purification: Column chromatography (pentane/EtOAc 95:5).

Rf = 0.65 (SiO_2, pentane/EtOAc, 9:1); **1H NMR (400 MHz, Acetone-*d_6*):** δ 4.94 (tt, J = 4.1, 1.3 Hz, 1H), 3.80 (s, 6H), 2.24 – 2.14 (m, 2H), 1.97 (s, 6H), 1.86 – 1.79 (m, 2H), 1.45 (t, J = 6.6 Hz, 2H), 0.93 (s, 6H); **^{13}C NMR (101 MHz, Acetone-*d_6*):** δ 196.1, 166.1, 151.9, 102.8, 92.8, 61.9, 56.2, 53.8, 38.4, 36.3, 31.5, 28.2, 26.6; **IR (neat):** 2954, 2925, 1751, 1720, 1680, 1435, 1248, 1152, 1130, 1050, 802 cm-1; **HRMS (ESI):** Calculated for C17H25BrO6 [M+Na]+: 427.0727; Found: 427.0720 m/z.

Dimethyl 2-(2-bromo-2-methylpropanoyl)-2-((3,3,5,5-tetramethylcyclohex-1-en-1-yl)oxy)malonate (**4i**)

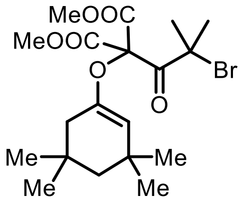

Following general procedure B and after 30 min, **4i** was obtained from enol ether **2i** (284 mg, 1 mmol) as a colorless oil (333 mg, 77% yield). Purification: Column chromatography (pentane/EtOAc 95:5).

^{1}H NMR (400 MHz, Acetone-*d$_6$*): δ 4.77 (s, 1H), 3.80 (s, 6H), 1.97 (s, 8H), 1.31 (s, 2H), 1.02 (s, 6H), 1.00 (s, 6H).; **^{13}C NMR (101 MHz, Acetone-*d$_6$*):** δ 196.0, 166.0, 150.3, 112.4, 61.8, 53.7, 50.3, 42.4, 33.4, 32.7, 32.5, 31.5, 30.6; **IR (neat):** 2953, 1753, 1720, 1676, 1434, 1248, 1221, 1146, 1137, 1097, 1049, 977, 845 cm^{-1}; **HRMS (ESI):** Calculated for $C_{19}H_{29}BrO_6$ [M+Na]$^+$: 455.1040; Found: 455.1038 m/z.

Dimethyl 2-(2-bromo-2-methylpropanoyl)-2-((4-(trifluoromethyl)cyclohex-1-en-1-yl)oxy) malonate (**4j**)

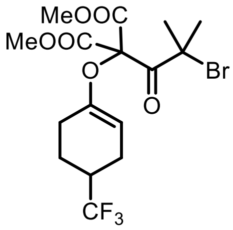

Following general procedure B and after 30 min, **4j** was obtained from enol ether **2j** (148 mg, 0.5 mmol) as a white solid (158 mg, 71% yield). Purification: Column chromatography (pentane/EtOAc 95:5).

Rf = 0.60 (SiO_2, pentane/EtOAc, 9:1); **^{1}H NMR (400 MHz, Acetone-*d$_6$*):** δ 5.19 – 4.87 (m, 1H), 3.83 (s, 3H), 3.82 (s, 3H), 2.47 – 2.23 (m, 4H), 2.19 – 2.11 (m, 1H), 2.11 – 2.07 (m, 1H), 1.96 (s, 6H), 1.64 (qd, J = 12.7, 6.2 Hz, 1H).; **^{13}C NMR (101 MHz, Acetone-*d$_6$*):** δ 195.8, 166.0, 165.6, 152.6, 129.0 (q, J = 277.8 Hz), 101.5, 92.8, 61.5, 53.8, 53.7, 38.5 (q, J = 26.8 Hz), 31.4, 31.2, 27.6, 23.3 (q, J = 3.0 Hz), 22.4 (q, J = 2.9 Hz); **^{19}F NMR (282 MHz, acetone-*d$_6$*):** δ -73.84; **IR (neat):** 2956, 1755, 1720, 1683, 1436, 1379, 1253, 1171, 1146, 1045, 988, 819 cm^{-1}; **HRMS (ESI):** Calculated for $C_{16}H_{20}BrF_3O_6$ [M+Na]$^+$: 467.0288; Found: 467.0300 m/z; **M.p.:** 77-79 °C

Dimethyl 2-(2-bromo-2-methylpropanoyl)-2-((4-methylcyclohex-1-en-1-yl)oxy)malonate (**4k**)

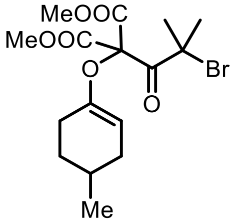

Following general procedure B and after 30 min, **4k** was obtained from enol ether **2k** (242 mg, 1 mmol) as a colorless oil (236 mg, 60% yield). Purification: Column chromatography (pentane/EtOAc 95:5).

Rf = 0.67 (SiO_2, pentane/EtOAc, 9:1); **^{1}H NMR (400 MHz, Acetone-*d$_6$*):** δ 5.04 – 5.00 (m, 1H), 3.81 (s, 3H), 3.79 (s, 3H), 2.30 – 2.18 (m, 2H), 2.17 – 2.08 (m, 1H), 1.96 (s, 6H), 1.77 – 1.55 (m, 3H), 1.40 – 1.24 (m, 1H), 0.95 (d, J = 6.4 Hz, 3H). ; **^{13}C NMR (101 MHz, Acetone-*d$_6$*):** δ 196.0, 166.1, 165.9, 152.8, 103.6, 92.9, 61.7, 53.7, 53.5, 32.6, 31.6, 31.4, 31.3, 28.8, 28.5, 21.3; **IR (neat):** 2953, 2925, 1753, 1719, 1679, 1435, 1247, 1226, 1150, 1047, 804 cm^{-1}; **HRMS (ESI):** Calculated for $C_{16}H_{23}BrO_6$ [M+Na]$^+$: 413.0571; Found: 413.0557 m/z.

Dimethyl 2-(2-bromo-2-methylpropanoyl)-2-((4-(*tert*-butyl)cyclohex-1-en-1-yl)oxy) malonate (**4l**)

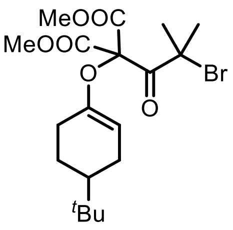

Following general procedure B and after 30 min, **4l** was obtained from enol ether **2l** (142 mg, 0.5 mmol) as a colorless oil (164 mg, 76% yield). Purification: Column chromatography (pentane/EtOAc 95:5).

Rf = 0.49 (SiO_2, pentane/EtOAc, 9:1); **^{1}H NMR (400 MHz, $CDCl_3$):** δ 5.02 (ddt, J = 6.0, 2.5, 1.2 Hz, 1H), 3.84 (s, 3H), 3.81 (s, 3H), 2.26 – 2.21 (m, 2H), 2.03 – 1.99 (m, 1H), 1.98 (s, 3H), 1.97 (s, 3H), 1.87 – 1.79 (m, 2H), 1.34 – 1.15 (m, 3H), 0.8 (s, 9H); **^{12}C NMR (101 MHz, $CDCl_3$):** δ196.1, 165.7, 165.3, 152.1, 103.5, 61.2, 53.6, 53.4, 44.9, 35.9, 32.2, 31.5, 29.3, 27.5, 25.1, 24.2; **IR (neat):** 2961, 1756, 1720, 1683, 1434, 1366, 1250, 1164, 1148, 1097, 1050, 979, 802 cm^{-1}; **HRMS (ESI):** Calculated for $C_{19}H_{29}BrO_6$ $[M+Na]^+$: 455.1040; Found: 455.1037 m/z.

Dimethyl (E)-2-(2-bromo-2-methylpropanoyl)-2-(cyclododec-1-en-1-yloxy)malonate (**4m**)

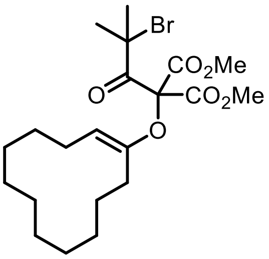

Following general procedure B and after 30 min, **4m** was obtained from enol ether **2m** (156 mg, 0.5 mmol) as a white solid (158 mg, 68% yield). Purification: Column chromatography (pentane/EtOAc 95:5).

Rf = 0.73 (SiO_2, pentane/EtOAc, 9:1); **^{1}H NMR (400 MHz, Acetone-*d_6*):** δ 4.63 (t, J = 7.8 Hz, 1H), 3.80 (s, 6H), 2.31 (t, J = 6.7 Hz, 2H), 1.98 (s, 6H), 1.81 – 1.67 (m, 2H), 1.49 – 1.22 (m, 16H); **^{13}C NMR (101 MHz, Acetone-*d_6*):** δ 195.7, 165.9, 153.1, 106.9, 92.3, 61.4, 53.6, 53.00, 31.3, 28.4, 27.3, 25.7, 25.4, 25.3, 24.8, 24.4, 23.8, 22.7 ; **IR (neat):** 2930, 2859, 1769, 1747, 1715, 1673 ,1467, 1246, 1140, 1133, 1051, 726 cm^{-1}; **HRMS (ESI):** Calculated for $C_{21}H_{33}BrO_6$ $[M+Na]^+$: 483.1353; Found: 483.1356 m/z ; **M.p.:** 50-53°C.

5. Synthesis and data analysis of *trans*-fused bicycles 5, 7i and 9a

General procedure C

In microwave vial equipped with a magnetic stirring bar, malonate enol ether **4** (0.2 mmol, 1 equiv), acridine orange (1.3 mg, 5 µmol, 2.5 mol%) and Hantzsch ester **HE** (127 mg, 0.5 mmol, 2.5 equiv) were added. The vial was sealed, degassed and flushed with N_2, and then dry acetonitrile (2 mL) was added. The mixture was stirred at room temperature (25-28 °C) under blue LEDs irradiation (λ_{max} = 455 nm) for 3 hours. The residue was filtered through a short pad of deactivated SiO_2, washed with EtOAc. The organic phase was evaporated *in vacuo* and purified by column chromatography to afford . In certain instances, additional crystallization was needed to afford pure *trans* fused cycles.

Pictures of the set-up: (distance between 455 nm LED and the center of the flask is around 10 cm)

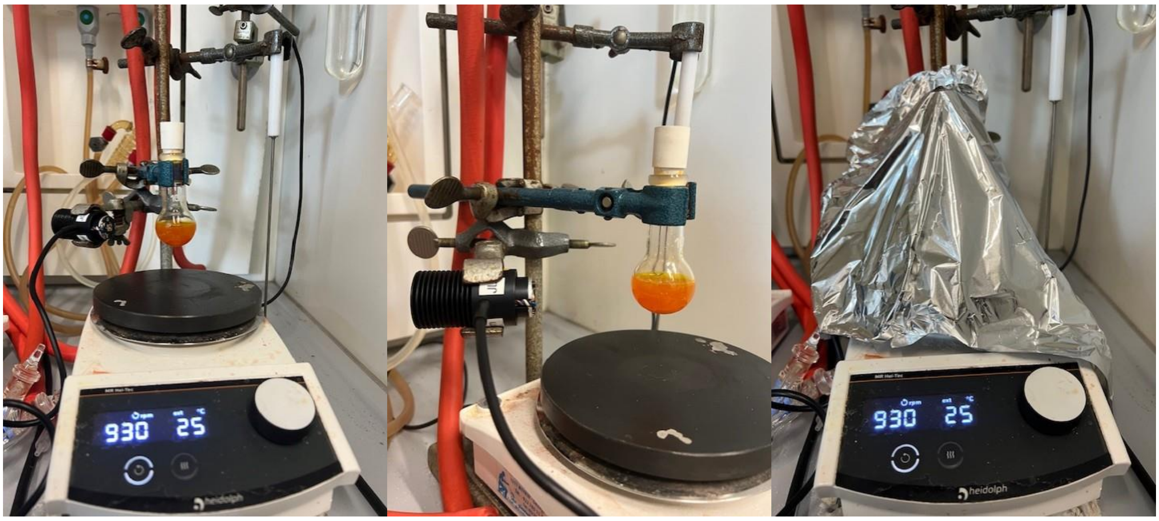

(pictures were taken on the 1 mmol scale experiment but remain the same with the microwave vial for 0.2 mmol scale).

General procedure D

In microwave vial equipped with a magnetic stirring bar, malonate enol ether **4** (0.2 mmol, 1 equiv), acridine orange (1.3 mg, 5 µmol, 2.5 mol%) and Hantzsch ester (127 mg, 0.5 mmol, 2.5 equiv) were added. The vial was sealed, degassed and flushed with N_2, and then dry acetonitrile (2 mL) was added. The mixture was stirred at room temperature (25-28 °C) under UV irradiation (λ_{max} = 365 nm) for 1.5 hours. Then DBU (1 equiv., 0.2 mmol, 30 µL) and LiCl (1.2 equiv., 0.24 mmol, 10.2 mg) were added. The vial was opened and stirred at room temperature for 30 min, then solvent was evaporated *in vacuo*. Treatment with 5 mL of HCl (6 N) was then performed to remove HE and oxidized HE, extraction with EtOAc (3 x 3 mL). The residue was then purified by column chromatography to afford **5**.

Dimethyl (3aR,7aR)-7a-hydroxy-3,3-dimethyl-2-oxooctahydro-1H-indene-1,1-dicarboxylate (**5a**)

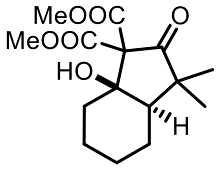

Following general procedure C, **5a** was obtained from acylated malonate enol ether **4a** (75 mg, 0.2 mmol) as a white solid (40 mg, 66% yield). Purification: Column chromatography (CH_2Cl_2/Pentane/Et_3N 9:1:0.1) and crystallization in pentane. (same conditions for 1 mmol scale experiment gave 64 % of **5a**)

^{1}H NMR (400 MHz, $CDCl_3$): δ 4.15 (d, J = 1.9 Hz, 1H), 3.85 (s, 3H), 3.76 (s, 3H), 2.02 (dt, J = 12.9, 3.1 Hz, 1H), 1.88 – 1.80 (m, 3H), 1.74 (qd, J = 12.1, 3.0 Hz, 1H), 1.67 – 1.58 (m, 1H), 1.58 – 1.45 (m, 2H), 1.35 – 1.22 (m, 1H), 1.22 (s, 3H), 1.16 (s, 3H); **^{13}C NMR (101 MHz, $CDCl_3$):** δ 210.9, 168.3, 166.6, 80.4, 75.3, 53.3, 53.2, 51.3, 47.9, 34.1, 26.5, 25.7, 22.6, 21.4, 20.6; **IR (neat):** 3534, 2945, 1761, 1734, 1707, 1432, 1237, 989, 969 cm^{-1}; **HRMS (ESI):** Calculated for $C_{15}H_{22}O_6$ $[M+Na]^+$: 321.1309; Found: 321.1299 m/z.

Dibenzyl (3aR,7aR)-7a-hydroxy-3,3-dimethyl-2-oxooctahydro-1H-indene-1,1-dicarboxylate (**5b**)

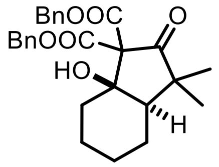

Following general procedure C, **5b** was obtained from acylated malonate enol ether **4b** (53 mg, 0.1 mmol) as a colorless oil (24 mg, 53% yield). Purification: preparative TLC (Hexane/THF 9:1).

^{1}H NMR (400 MHz, $CDCl_3$): δ 7.35 – 7.28 (m, 8H), 7.23 – 7.20 (m, 2H), 5.21 (d, J = 14.9 Hz, 2H), 5.10 (d, J = 2.9 Hz, 2H), 4.15 (d, J = 1.9 Hz, 1H), 2.00 – 1.95 (m, 1H), 1.83 – 1.70 (m, 4H), 1.59 – 1.52 (m, 1H), 1.49 – 1.40 (m, 2H), 1.24 – 1.20 (m, 1H), 1.17 (s, 3H), 1.16 (s, 3H); **^{13}C NMR (101 MHz, $CDCl_3$):** δ 210.6, 167.6, 166.0, 135.0, 134.6, 128.8, 128.7, 128.7, 128.6, 128.4, 128.3, 128.0, 80.5, 75.5, 68.0, 67.9, 51.2, 47.8, 34.1, 26.3, 25.7, 22.7, 21.4, 20.5; **HRMS (ESI):** Calculated for $C_{27}H_{30}O_6$ $[M+Na]^+$: 473.1935; Found: 473.1929 m/z.

Dimethyl (3aR,8aR)-8a-hydroxy-3,3-dimethyl-2-oxooctahydroazulene-1,1(2H)-dicarboxylate (**5c**)

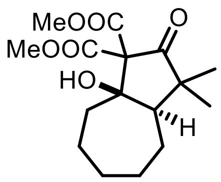

Following general procedure C, **5c** was obtained from acylated malonate enol ether **4c** (78 mg, 0.2 mmol) as a white solid (35 mg, 58% yield). Purification: Column chromatography (CH_2Cl_2/Pentane/Et_3N 9:1:0.1) and crystallization in pentane.

^{1}H NMR (400 MHz, $CDCl_3$): δ 4.04 (d, J = 2.0 Hz, 1H), 3.85 (s, 3H), 3.75 (s, 3H), 2.18 (ddd, J = 14.3, 5.8, 2.7 Hz, 1H), 1.94 (dt, J = 11.0, 2.2 Hz, 1H), 1.89 – 1.33 (m, 9H), 1.19 (s, 3H), 1.14 (s, 3H); **^{13}C NMR (101 MHz, $CDCl_3$):** δ 211.2, 168.2, 166.8, 83.2, 76.3, 53.3, 53.2, 53.0, 49.9, 37.4,

27.2, 26.2, 25.9, 22.4, 21.2, 21.1; **HRMS (ESI):** Calculated for $C_{16}H_{24}O_6$ $[M+Na]^+$: 335.1466; Found: 335.1475 m/z.

Dimethyl (3aR,7aR)-5,5-difluoro-7a-hydroxy-3,3-dimethyl-2-oxooctahydro-1H-indene-1,1-dicarboxylate (**5e**)

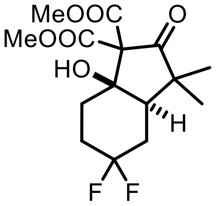

Following general procedure C, **5e** was obtained from acylated malonate enol ether **4e** (83 mg, 0.2 mmol) as a white solid (40 mg, 51% yield). Purification: Column chromatography (CH_2Cl_2/Pentane/Et_3N 8:2:0.1).

^{1}H NMR (400 MHz, $CDCl_3$): δ 4.52 (d, J = 2.0 Hz, 1H), 3.89 (s, 3H), 3.78 (s, 3H), 2.44 – 2.20 (m, 2H), 2.19 – 2.04 (m, 2H), 2.04 – 1.93 (m, 1H), 1.87 – 1.75 (m, 1H), 1.25 (s, 3H), 1.20 (s, 3H); **^{13}C NMR (101 MHz, $CDCl_3$):** δ 209.1, 168.1, 165.9, 123.35 (dd, J = 244.8, 239.8 Hz), 78.4, 74.2, 53.5, 53.4, 26.2, 22.5; **^{19}F NMR (282 MHz, $CDCl_3$):** δ -89.89 (d, J = 239.1 Hz), -99.98 (d, J = 239.1 Hz); **IR (neat):** 3676, 3527, 2988, 2970, 2902, 1767, 1733, 1708, 1438, 1366, 1233, 1027, 895, 682 cm^{-1}. **HRMS (ESI):** $C_{15}H_{20}F_2O_6$ $[M+Na]^+$: 357.1120; Found: 357.1103 m/z.

Dimethyl (3aR,7aR)-7a-hydroxy-3,3-dimethyl-2-oxohexahydrospiro[indene-5,2'-[1,3]dioxolane]-1,1(4H)-dicarboxylate (**5f**)

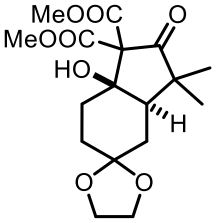

Following general procedure C and after 16 h, **5f** was obtained from acylated malonate enol ether **4f** (87 mg, 0.2 mmol) as a colorless oil (35 mg, 49% yield). Purification: preparative TLC (Pentane/ EtOAc, 8:2, Rf= 0.13).

^{1}H NMR (400 MHz, $CDCl_3$): δ 4.28 (d, J = 2.0 Hz, 1H), 4.00 – 3.92 (m, 4H), 3.86 (s, 3H), 3.77 (s, 3H), 2.22 – 2.00 (m, 4H), 1.82 (dd, J = 13.5, 4.3 Hz, 1H), 1.74 – 1.67 (m, 1H), 1.62 – 1.56 (m, 1H), 1.22 (s, 3H), 1.16 (s, 3H); **^{13}C NMR (101 MHz, $CDCl_3$):** δ 210.4, 168.3, 166.3, 108.9, 79.3, 74.8, 64.6, 64.5, 53.5, 53.4, 48.4, 47.7, 31.2, 30.5, 30.3, 26.4, 22.6; **HRMS (ESI):** Calculated for $C_{17}H_{24}O_8$ $[M+Na]^+$: 379.1364; Found: 379.1363 m/z.

5,5-diethyl 1,1-dimethyl (3aR,7aR)-7a-hydroxy-3,3-dimethyl-2-oxohexahydro-1H-indene-1,1,5,5(4H)-tetracarboxylate (**5g**)

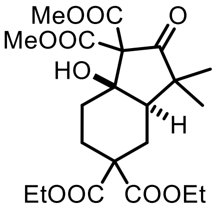

Following general procedure C, **5g** was obtained from acylated malonate enol ether **4g** (104 mg, 0.2 mmol) in 38% NMR yield. After several purification attempts, **5g** was only obtained as a minor compound in a mixture with an unknown by-product.

Dimethyl (3aR,7aR)-7a-hydroxy-3,3,5,5-tetramethyl-2-oxooctahydro-1H-indene-1,1-dicarboxylate (**5h**)

Following general procedure C, **5h** was obtained from acylated malonate enol ether **4h** (36 mg, 0.1 mmol) in 38% NMR yield. After several purification attempts, **5h** was only obtained as a minor compound in a mixture.

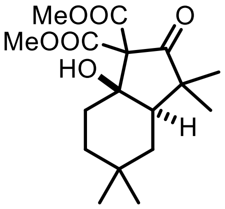

Dimethyl (3aR,7aR)-7a-hydroxy-3,3-dimethyl-2-oxo-5-(trifluoromethyl)octahydro-1H-indene-1,1-dicarboxylate (**5j**)

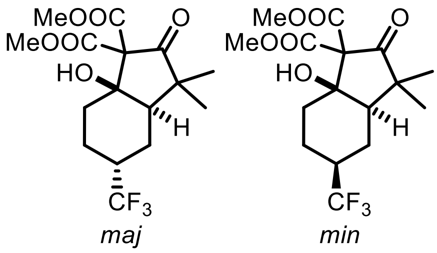

Following general procedure C, **5j** was obtained from acylated malonate enol ether **4j** (89 mg, 0.2 mmol) as a mixture of two diastereomers (*d.r.* 3:1, 44% overall NMR yield). Purification: Column chromatography (CH_2Cl_2/Pentane/Et_3N 9:1:0.1) and crystallization in pentane. Unfortunately, only the major isomer was recovered as a white solid in 14% yield and was characterized.

^{1}H NMR (400 MHz, $CDCl_3$): δ 4.37 (d, J = 1.9 Hz, 1H), 3.86 (s, 3H), 3.75 (s, 3H), 2.51 (qt, J = 11.9, 6.2 Hz, 1H), 2.25 – 2.13 (m, 2H), 2.07 (td, J = 13.4, 5.9 Hz, 1H), 1.98 – 1.89 (m, 2H), 1.88 – 1.76 (m, 2H), 1.22 (s, 3H), 1.17 (s, 3H); **^{13}C NMR (101 MHz, $CDCl_3$):** δ 209.8, 168.3, 166.2, 79.4, 75.0, 53.5, 53.3, 47.7, 45.3, 36.38 (q, J = 25.7 Hz), 30.40 (q, J = 20.3 Hz), 30.1, 26.5, 22.2, 19.1, 18.6; **^{19}F NMR (282 MHz, acetone-*d_6*):** δ -67.20;); **IR (neat):** 3452, 2956, 1759, 1736, 1713, 1438, 1270, 1220, 968, 790, 669 cm^{-1}. **HRMS (ESI):** $C_{16}H_{21}F_3O_6$ $[M+Na]^+$: 389.1183; Found: 389.1180 m/z.

Dimethyl (3aR,7aR)-7a-hydroxy-3,3,5-trimethyl-2-oxooctahydro-1H-indene-1,1-dicarboxylate (**5k**)

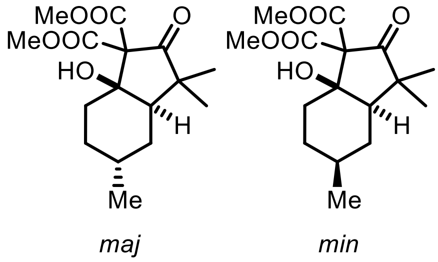

Following general procedure C, **5k** was obtained from acylated malonate enol ether **4k** (78 mg, 0.2 mmol) as a mixture of two diastereomers (*d.r.* 2:1, 55% overall NMR yield). Purification: Column chromatography (CH_2Cl_2/Pentane/Et_3N 9:1:0.1) and crystallization in

pentane. Unfortunately, only the major isomer was recovered as a white solid in 18% yield and was characterized.

^{1}H NMR (400 MHz, CDCl$_3$): δ 4.07 (d, J = 1.9 Hz, 1H), 3.85 (s, 3H), 3.77 (s, 3H), 2.20 – 1.95 (m, 4H), 1.86 – 1.71 (m, 2H), 1.43 – 1.36 (m, 1H), 1.27 – 1.24 (m, 1H), 1.18 (s, 3H), 1.15 (s, 3H), 1.01 (d, J = 7.2 Hz, 3H); **^{13}C NMR (101 MHz, CDCl$_3$):** δ 210.9, 168.2, 166.6, 80.6, 75.7, 53.3, 53.2, 47.6, 44.5, 28.8, 26.7, 26.4, 26.1, 22.5, 18.0; **HRMS (ESI):** Calculated for $C_{16}H_{24}O_6$ $[M+Na]^+$: 335.1466; Found: 335.1463 m/z.

Dimethyl (3aR,13aR)-13a-hydroxy-3,3-dimethyl-2-oxotetradecahydro-1H-cyclopenta [12]annulene-1,1-dicarboxylate (**5m**) and dimethyl 2-(2-methyl-2-(2-oxocyclododecyl) propanoyl) malonate (**6m**)

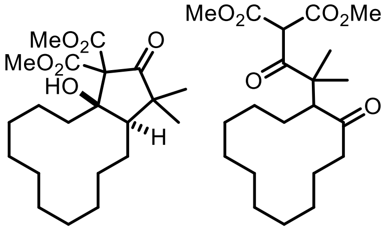

5m minor **6m** major

Following general procedure C and after 16 h, **5m** and **6m** were obtained from acylated malonate enol ether **4m** (92 mg, 0.2 mmol) as an inseparable mixture (ratio 1:5, 75% overall yield). Purification: Column chromatography (CH_2Cl_2/Pentane/Et_3N 9:1:0.1).

^{1}H NMR (400 MHz, CDCl$_3$): δ 4.95 (s, 1H, maj), 4.21 (d, J = 2.2 Hz, 1H, min), 3.84 (s, 3H, min), 3.78 (s, 3H, maj), 3.77 (s, 3H, maj), 3.72 (s, 3H, min), 3.14 (dd, J = 10.9, 2.4 Hz, 1H, maj), 2.63 (ddd, J = 16.8, 9.1, 3.5 Hz, 1H, maj), 2.45 (ddd, J = 16.8, 8.3, 3.5 Hz, 1H, maj), 2.14 (ddd, J = 6.9, 4.9, 2.2 Hz, 1H, min), 2.01 – 1.93 (m, 2H, min), 1.86 – 1.71 (m, 1H, maj), 1.69 – 1.58 (m, 2H, maj), 1.52 – 1.11 (m, 18H maj + 18H min), 1.19 (m, 6H maj + 6H min); **^{13}C NMR (101 MHz, CDCl$_3$):** δ 214.3 (C=O, maj), 211.2 (C=O, min), 202.7 (C=O, maj), 168.3 (C=O, min), 166.6 (C=O, min), 165.1 (C=O, maj), 165.0 (C=O, maj), 84.2 (C-O, min), 74.9 (C, min), 60.2 (CH, maj), 56.0 (CH, maj), 53.4 (CH_3, maj), 53.4 (CH_3, maj), 53.3 (CH_3, min), 53.1 (CH_3, min), 51.4 (C, maj), 49.5 (C, min), 49.4 (CH, min), 43.5 (CH_2, maj), 35.5 (CH_2, min), 29.8 (C), 27.8 (CH_3, min), 27.5 (CH_2, maj), 27.0 (CH_2, maj), 26.3 (CH_2, min), 26.2 (CH_2, maj), 26.0 (CH_2, maj), 25.8 (CH_2, maj), 25.4 (CH_2, min), 25.0 (CH_2, maj), 24.9 (CH_2, maj), 24.1 (CH_2, min), 24.0 (CH_2, min), 23.7 (CH_2, min), 23.2 (CH_2, min), 23.1 (CH_3, maj), 22.9 (CH_2, maj), 22.9 (CH_3, min), 22.4 (CH_2, maj), 21.3 (CH_2, min), 20.7 (CH_3, maj), 20.6 (CH_2, min).

Dimethyl 4,4,7,7,9,9-hexamethyl-3-oxo-1-oxaspiro[4.5]decane-2,2-dicarboxylate (**7i**)

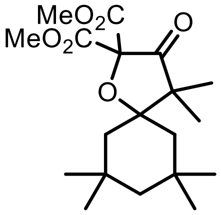

Following general procedure C, **7i** was obtained from acylated malonate enol ether **4i** (37 mg, 0.09 mmol) as a colorless oil (7 mg, 23% yield). Purification: preparative TLC (pentane/EtOAc; 9:1).

^{1}H NMR 500 MHz, ($CDCl_3$): δ 3.82 (s, 3H), 1.76 (s, 3H), 1.64 (d, *J*=13.6 Hz, 2H), 1.63 (s, 3H), 1.53 (d, *J*=13.6 Hz, 2H), 1.29 (d, *J*=13.9 Hz, 1H), 1.19 (d, *J*=13.9 Hz, 1H), 1.10 (s, 6H), 0.98 (s,6H). **^{13}C NMR 100 MHz, ($CDCl_3$):** 168.3, 141.4, 114, 108.2, 82.5, 53.3, 51.4, 46.8, 33.8, 29.6, 19, 18.5. **IR (neat):** 2951, 2923, 2862, 1751, 1456, 1435, 1353, 1263, 1105, 1052, 979, 876, 783 cm^{-1}. **HRMS (ESI):** Calculated for $C_{19}H_{30}O_6Na$ $[M+Na]^+$: 377.1935; Found: 377.1926 m/z.

Synthesis of 1,1-dimethyl-2-oxo-2,4,5,6,7,7a-hexahydro-1H-indene-3-carboxylate (**9a**)

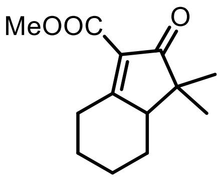

In microwave vial equipped with a magnetic stirring bar, *trans*-fused cycle **5a** (0.1 mmol, 1 equiv., 30 mg) was dissolved in dry THF (1 mL) and flushed with N_2. Then ^{t}BuOK (0.12 mmol, 1.2 equiv., 14 mg) was added and the mixture was stirred at room temperature (25-28 °C) for 1 hour. The solvent was then evaporated, and the residue was purified by column chromatography (pentane/EtOAc 9:1) to afford methyl 1,1-dimethyl-2-oxo-2,4,5,6,7,7a-hexahydro-1H-indene-3-carboxylate **9a** as a colorless oil (19 mg, 85 % yield).

^{1}H NMR (400 MHz, $CDCl_3$): δ 3.83 (s, 3H), 3.57 (m, 1H), 2.37 (dd, J = 12.8, 3.9 Hz, 1H), 2.27 – 2.18 (m, 1H), 2.09 – 2.03 (m, 1H), 1.59 – 1.37 (m, 2H), 1.29 – 1.21 (m, 1H), 1.10 (s, 3H), 1.00 (s, 3H); **^{13}C NMR (101 MHz, $CDCl_3$):** δ 208.0, 188.1, 164.2, 126.6, 53.6, 51.9, 46.5, 30.2, 30.1, 27.1, 26.3, 25.3, 20.2; **HRMS (ESI):** Calculated for $C_{13}H_{18}O_3$ $[M+Na]^+$: 245.1149 ; Found: 245.1155 m/z.

6. NMR spectra

Compound 2e ^{1}H NMR (400 MHz, acetone-d_6)

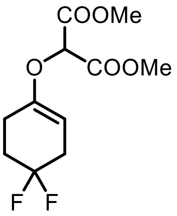

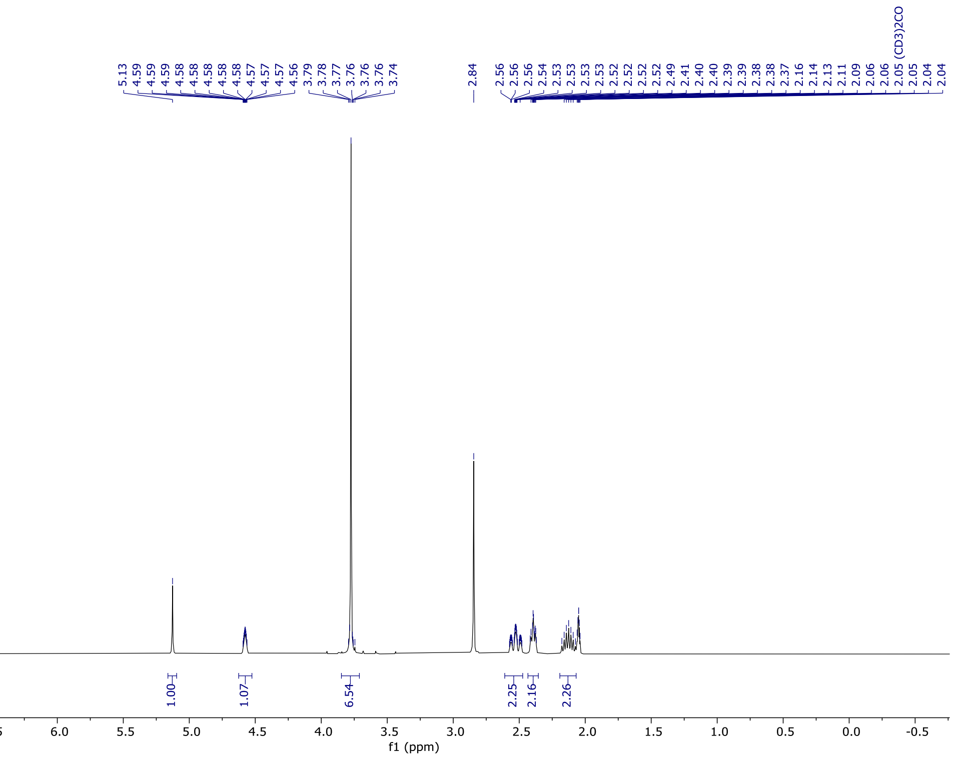

Compound 2e ^{13}C NMR (101 MHz, acetone-d_6)

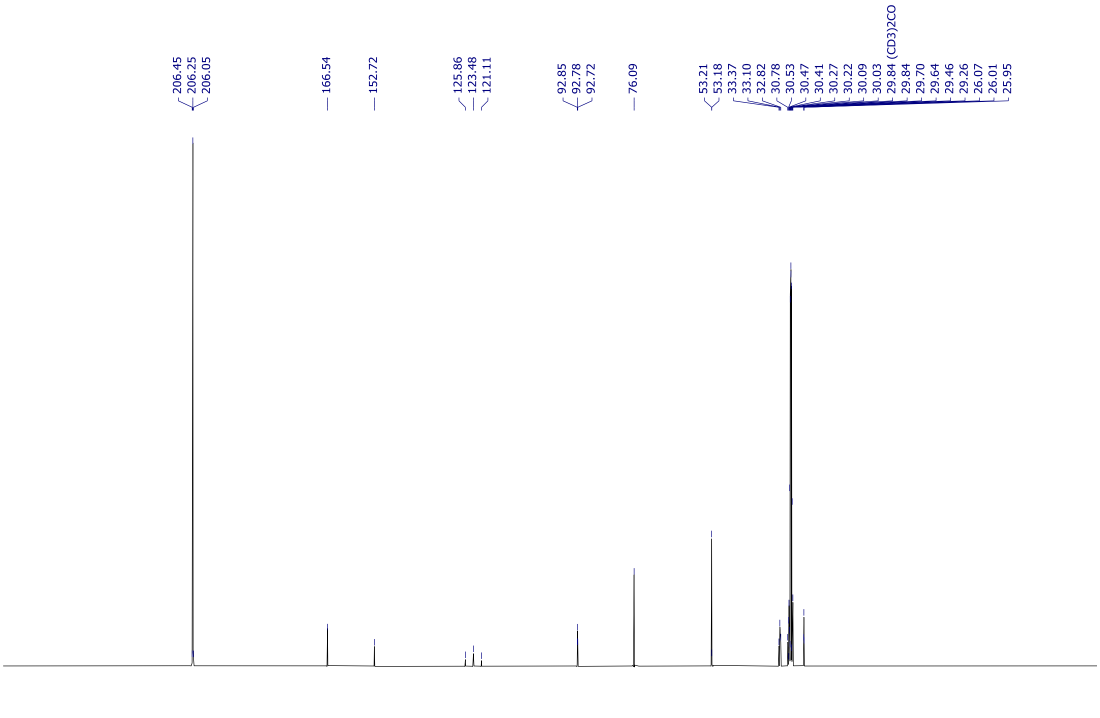

Compound 2f ^{1}H NMR (400 MHz, acetone-d_6)

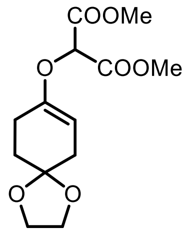

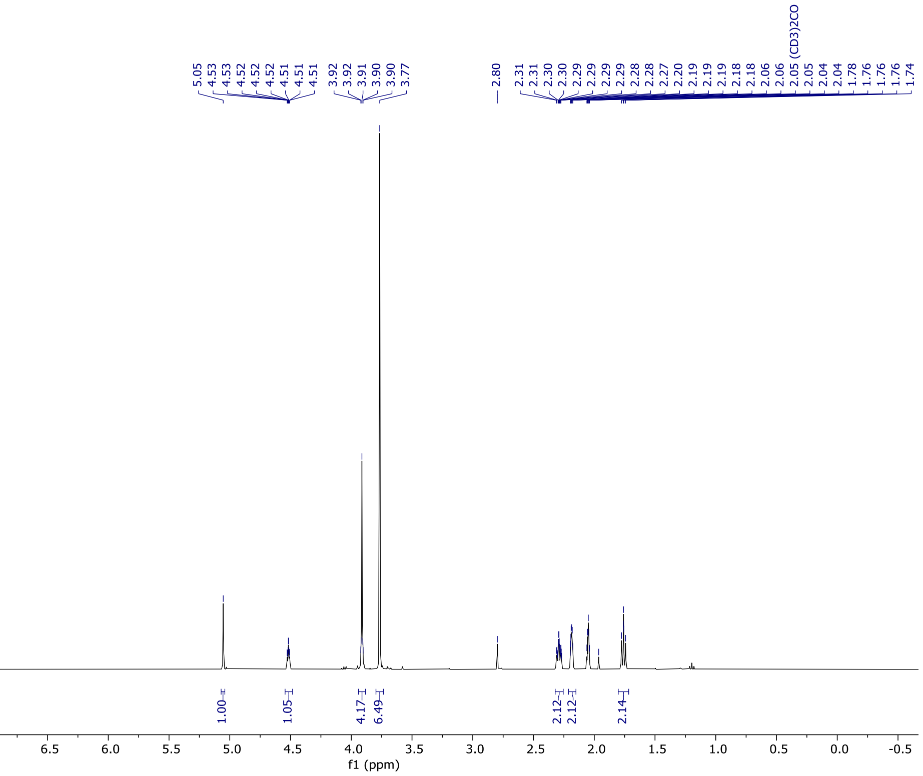

Compound 2f ^{13}C NMR (101 MHz, acetone-d_6)

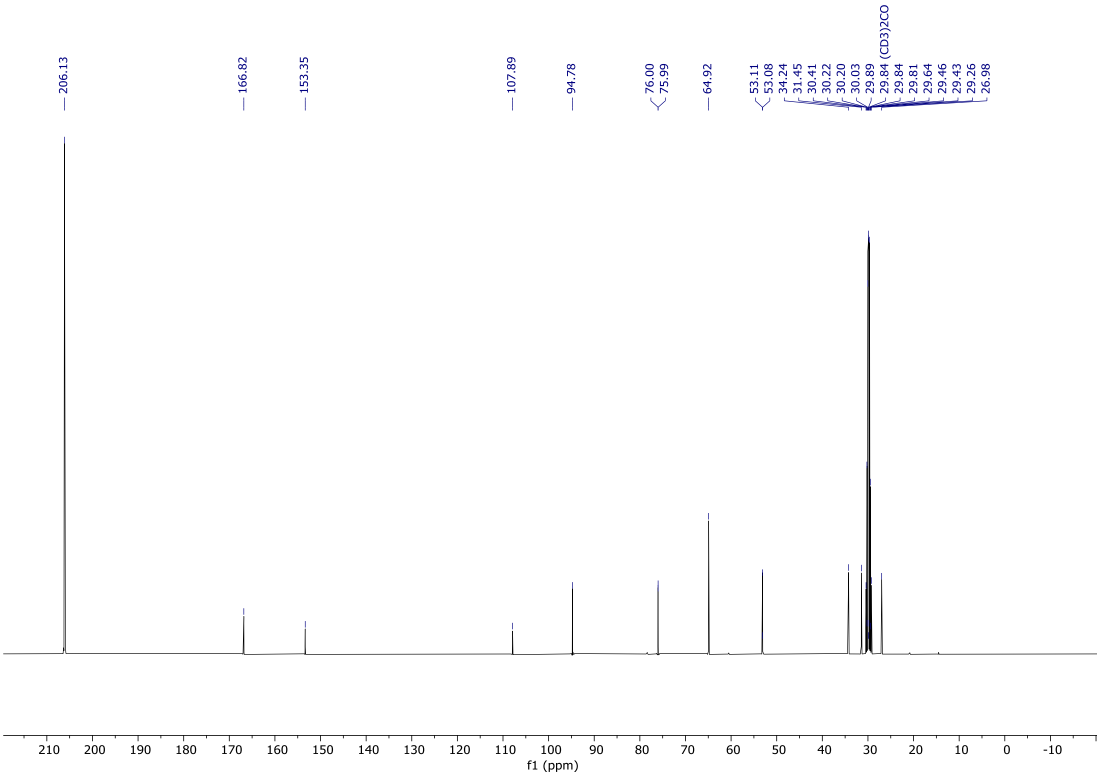

Compound 2g ^{1}H NMR (400 MHz, acetone-*d_6*)

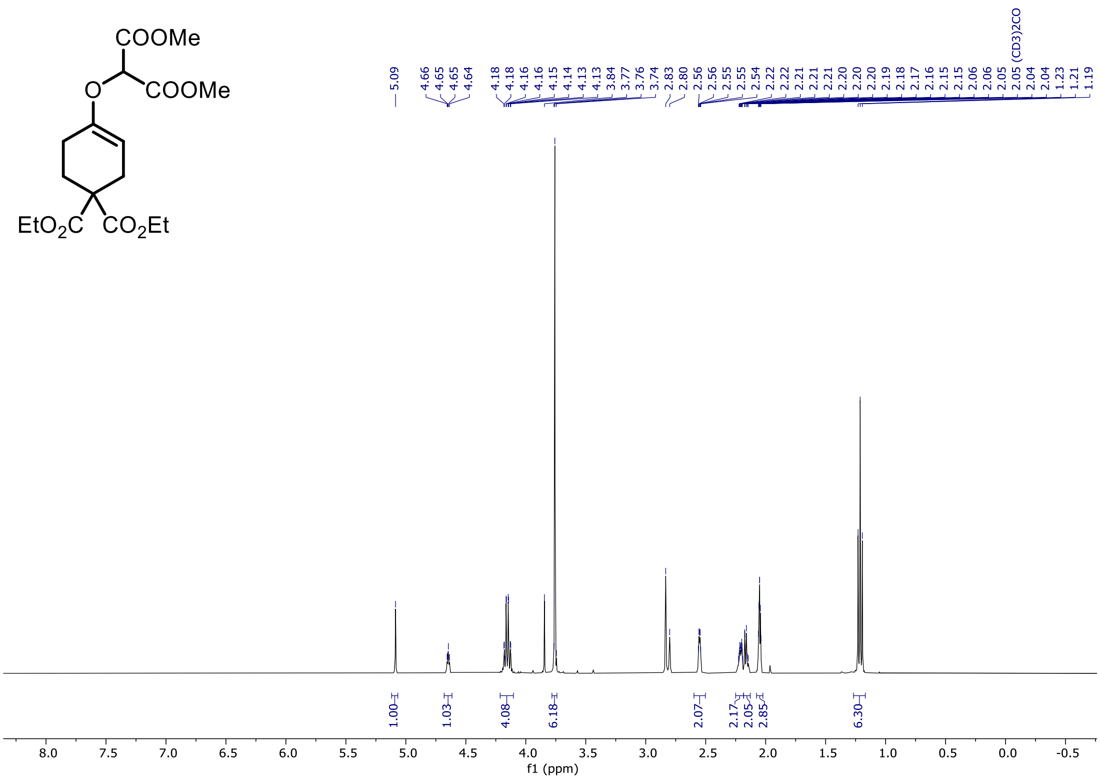

Compound 2g ^{13}C NMR (101 MHz, acetone-*d_6*)

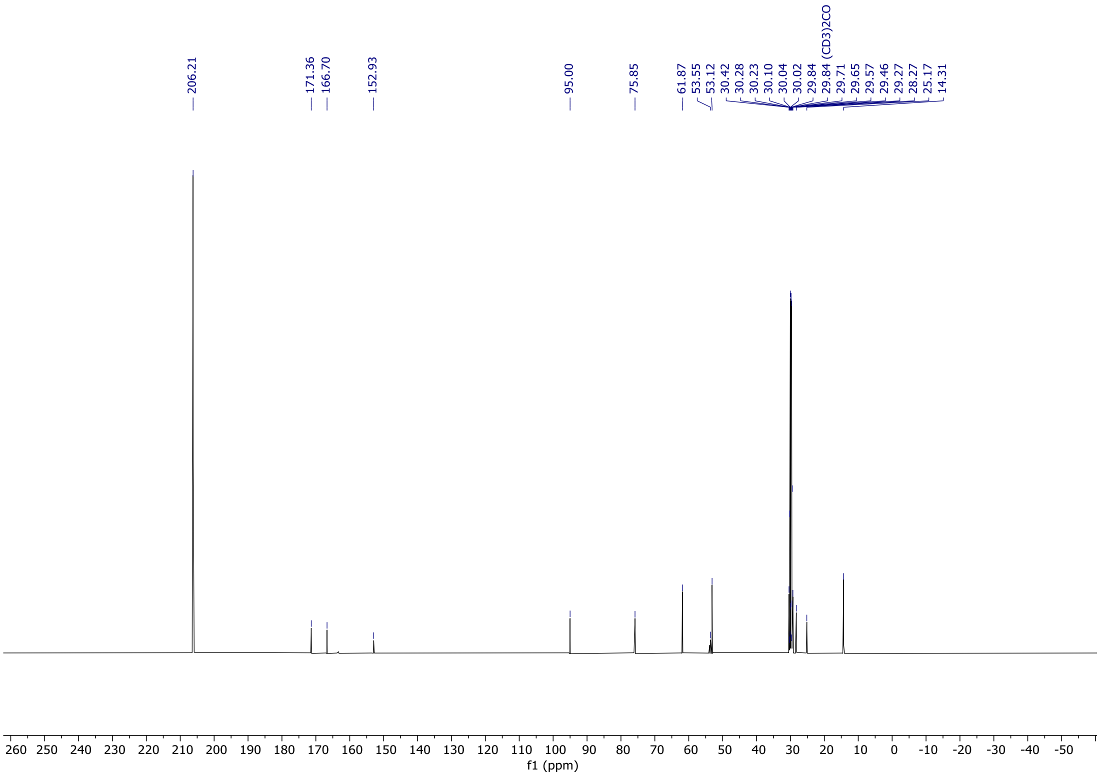

Compound 2h ^{1}H NMR (400 MHz, acetone-d_6)

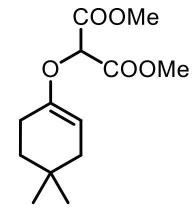

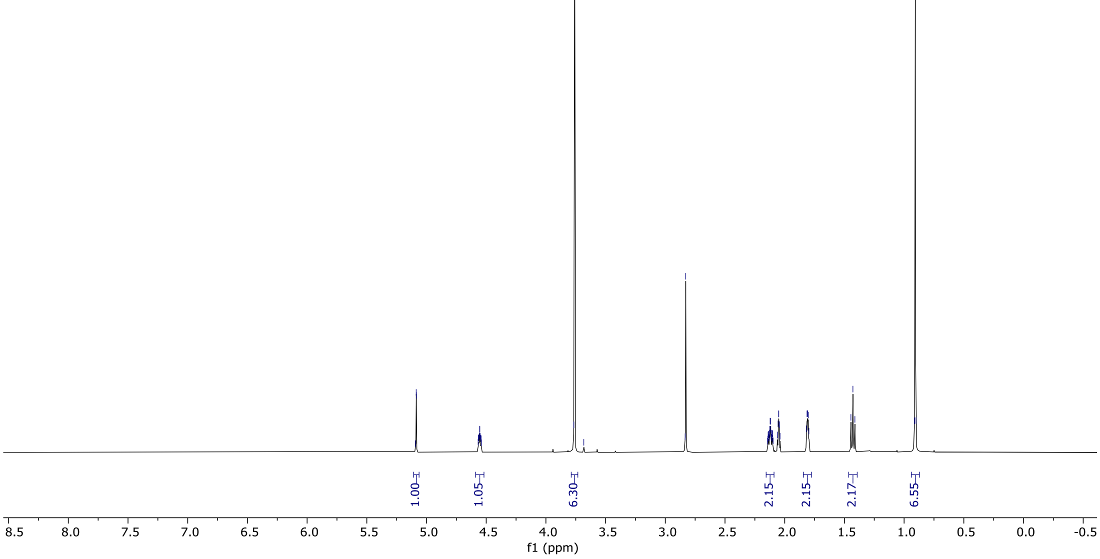

Compound 2h ^{13}C NMR (101 MHz, acetone-d_6)

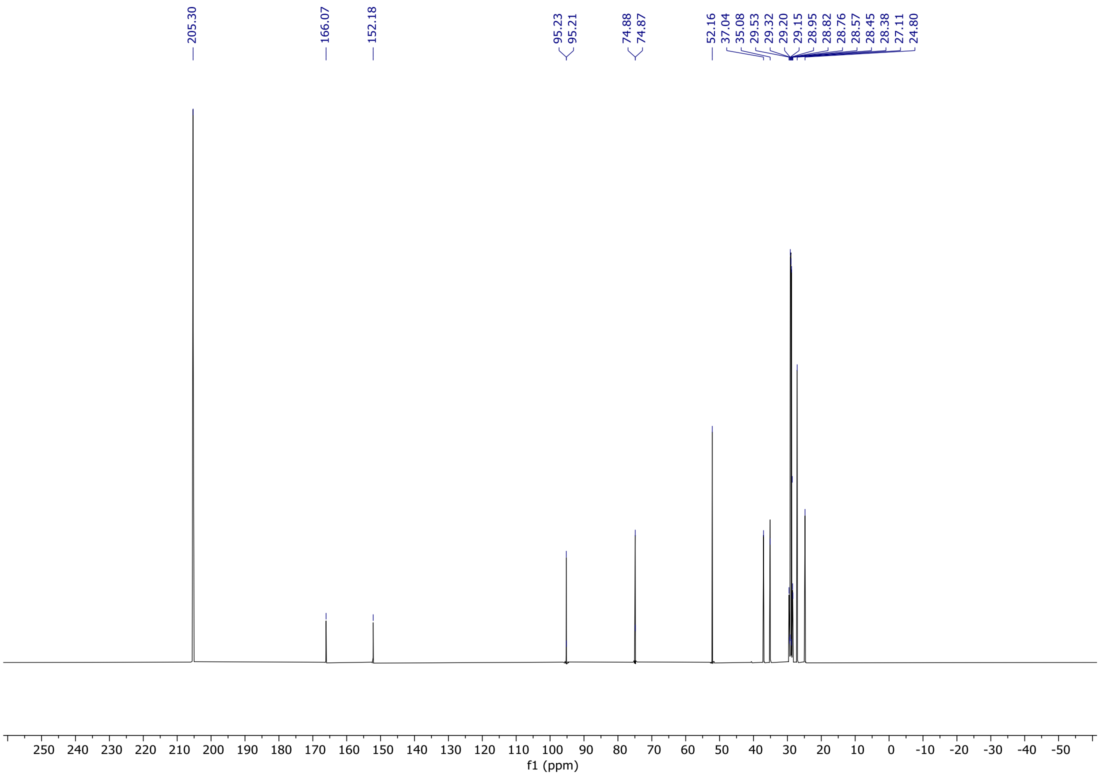

Compound 2i ^{1}H NMR (400 MHz, acetone-*d_6*)

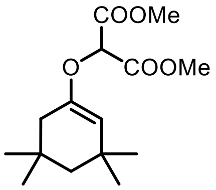

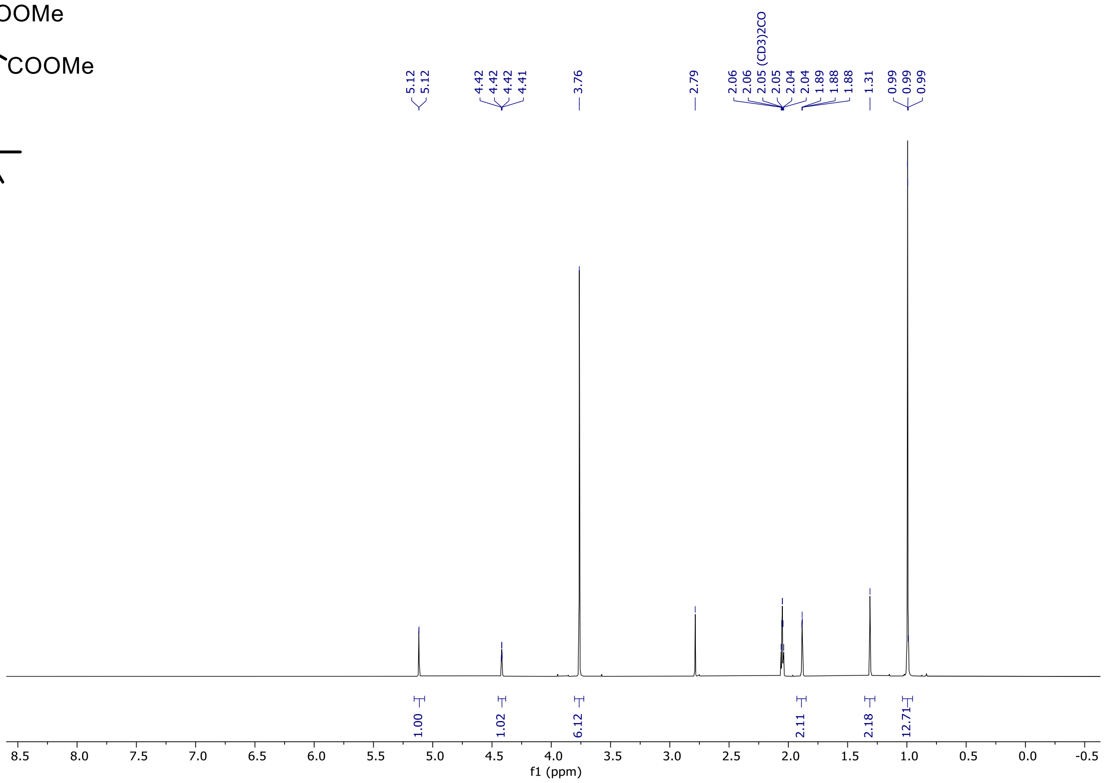

Compound 2i ^{13}C NMR (101 MHz, acetone-*d_6*)

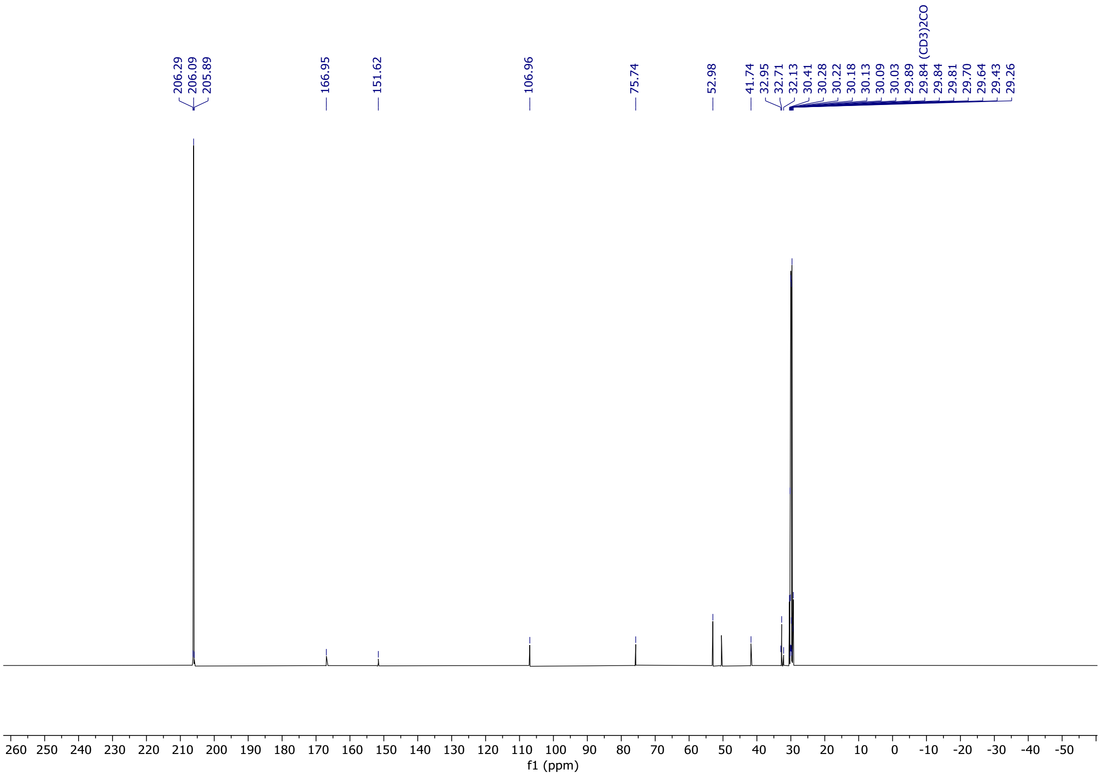

Compound 2k ^{1}H NMR (400 MHz, acetone-d_6)

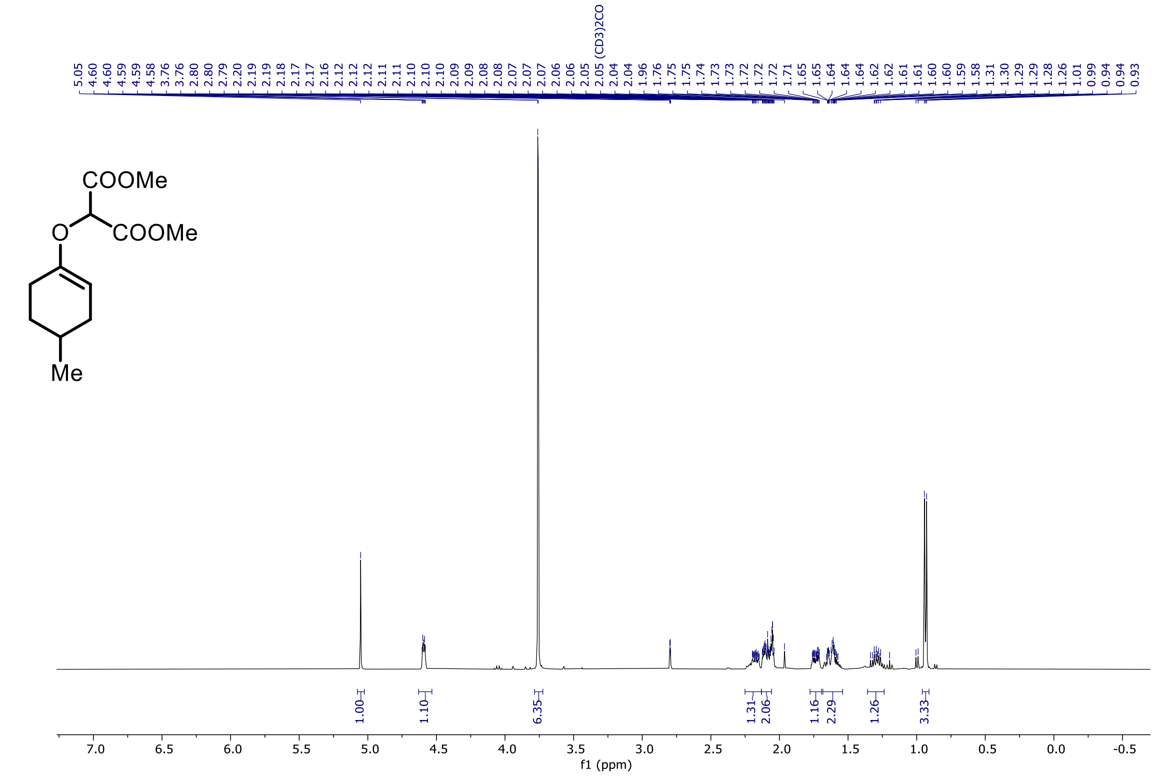

Compound 2k ^{13}C NMR (101 MHz, acetone-d_6)

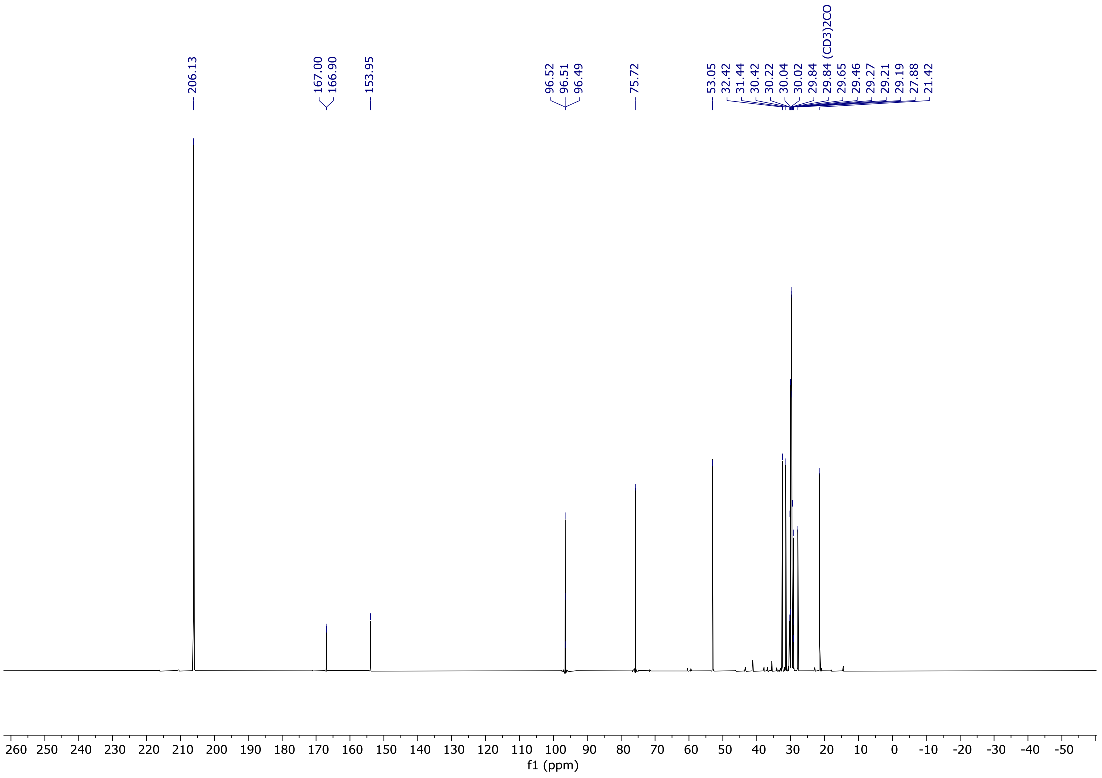

Compound 4a ^{1}H NMR (400 MHz, acetone-d_6)

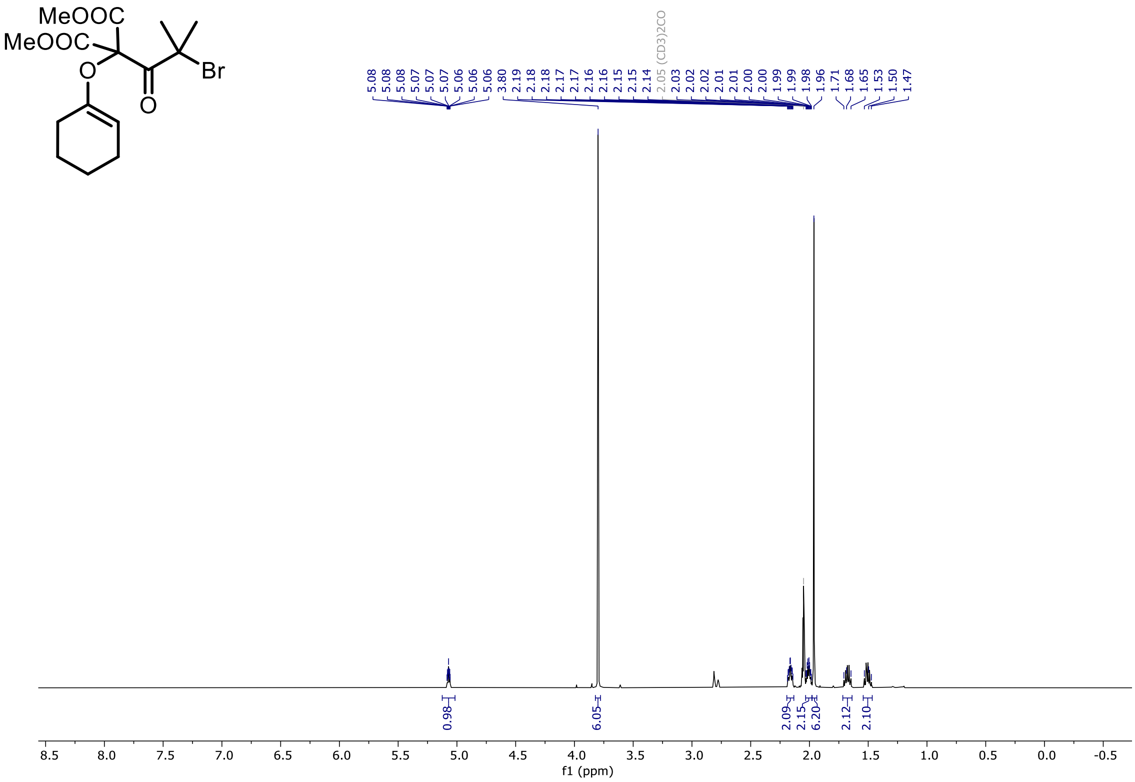

Compound 4a ^{13}C NMR (101 MHz, acetone-d_6)

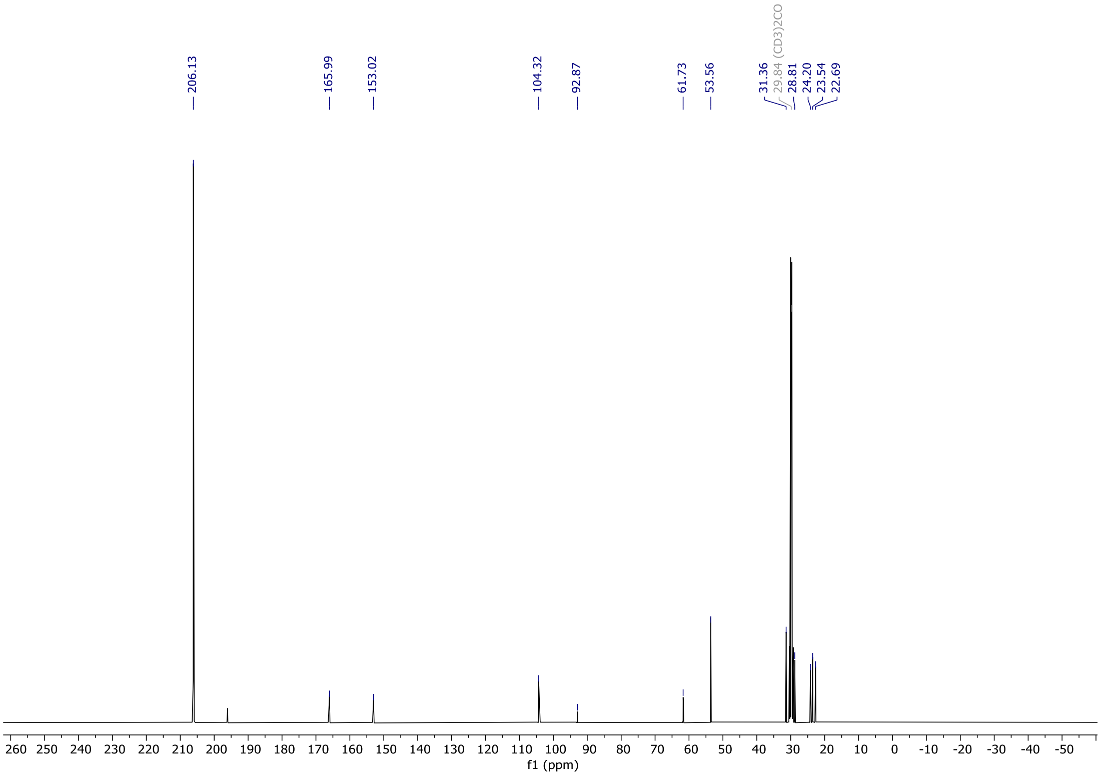

Compound 4b ^{1}H NMR (400 MHz, acetone-d_6)

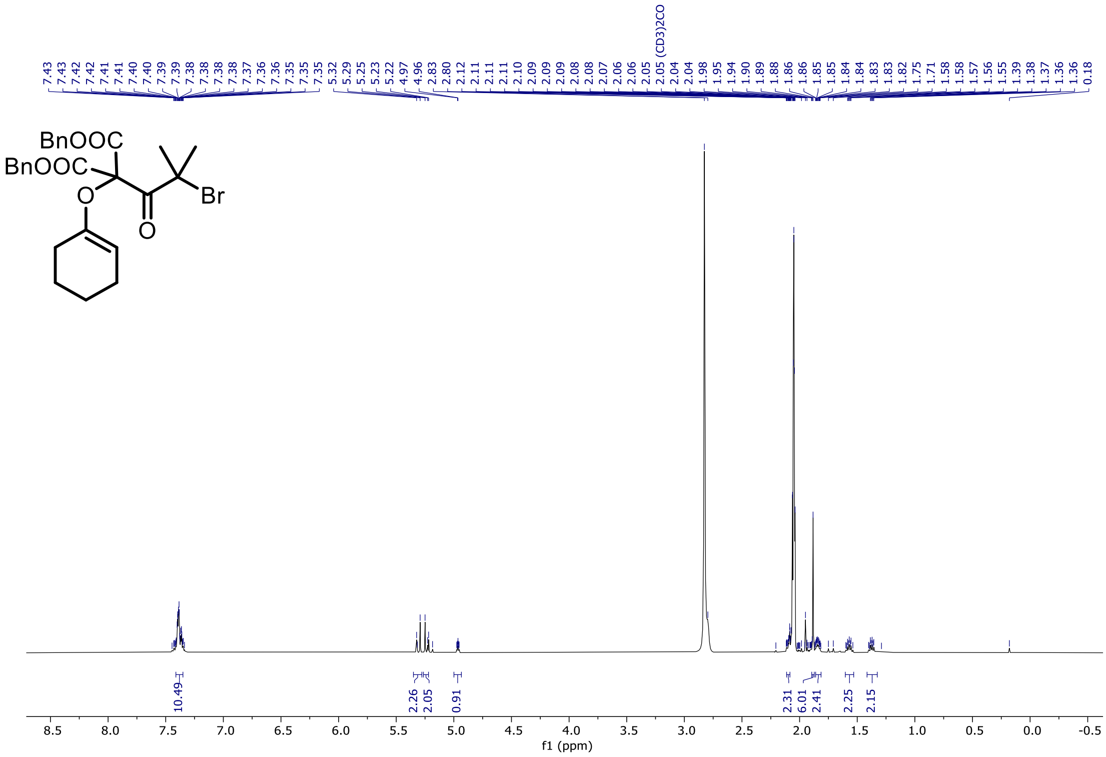

Compound 4b ^{13}C NMR (101 MHz, acetone-d_6)

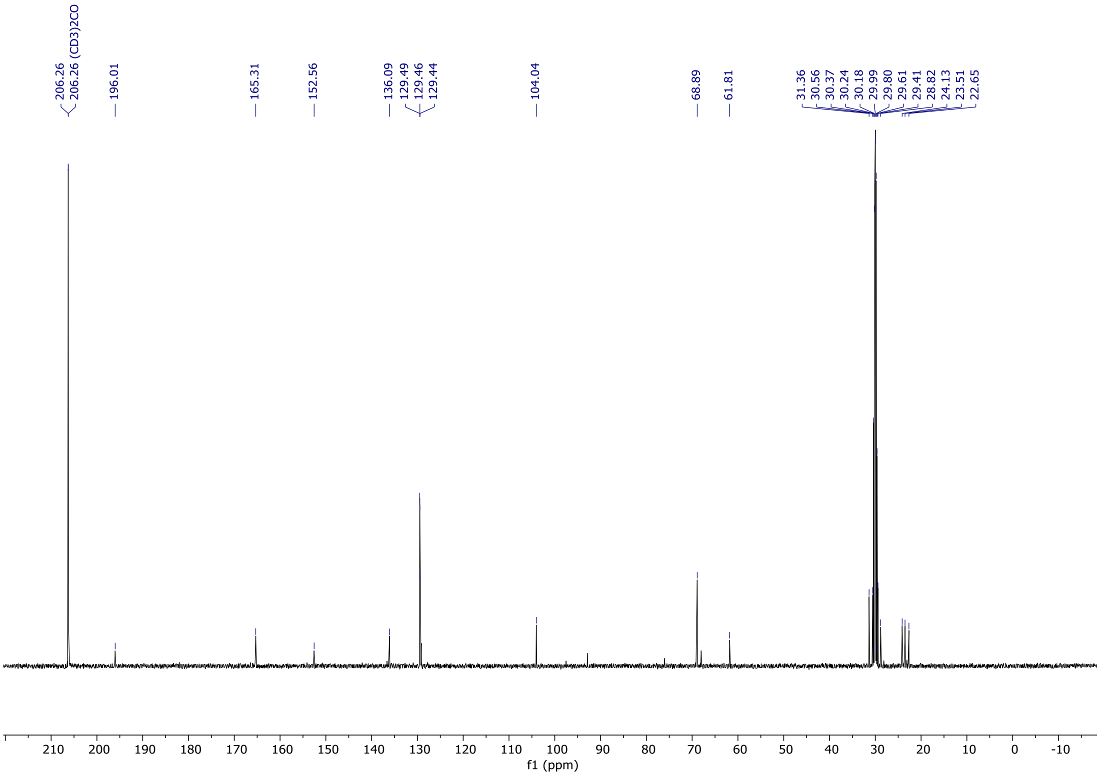

Compound 4c ^{1}H NMR (400 MHz, acetone-d_6)

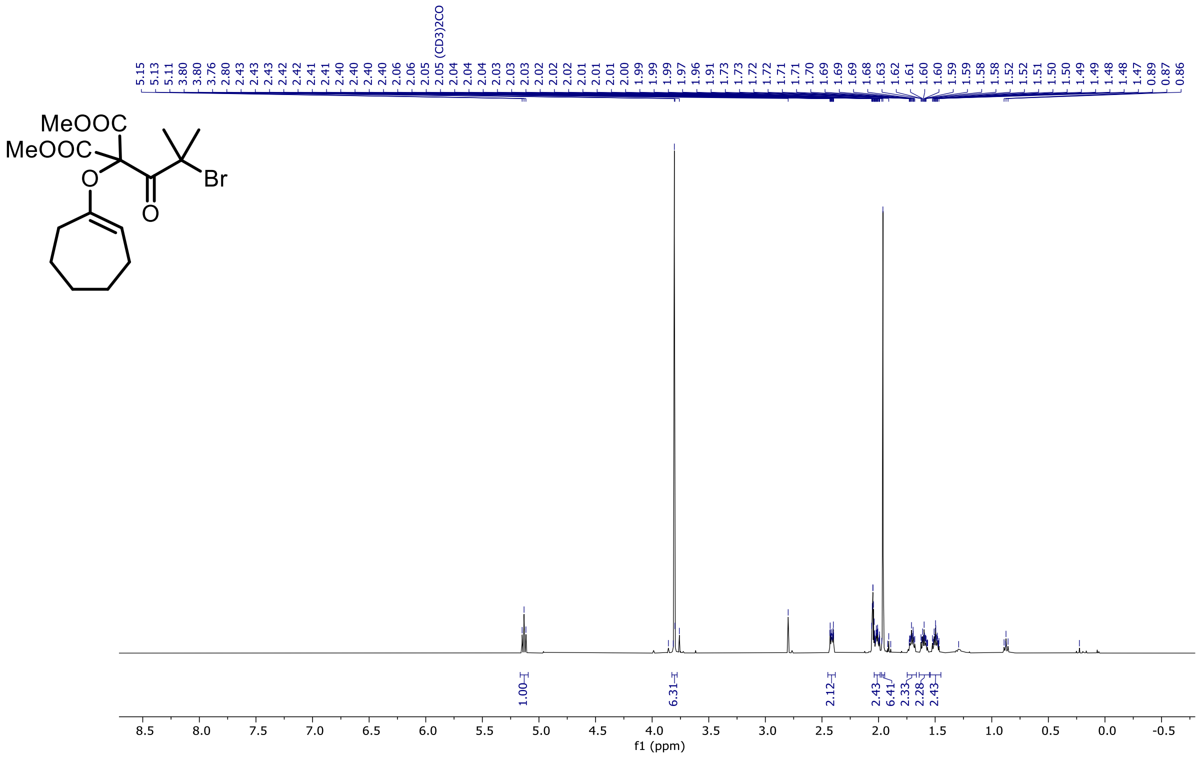

Compound 4c ^{13}C NMR (101 MHz, acetone-d_6)

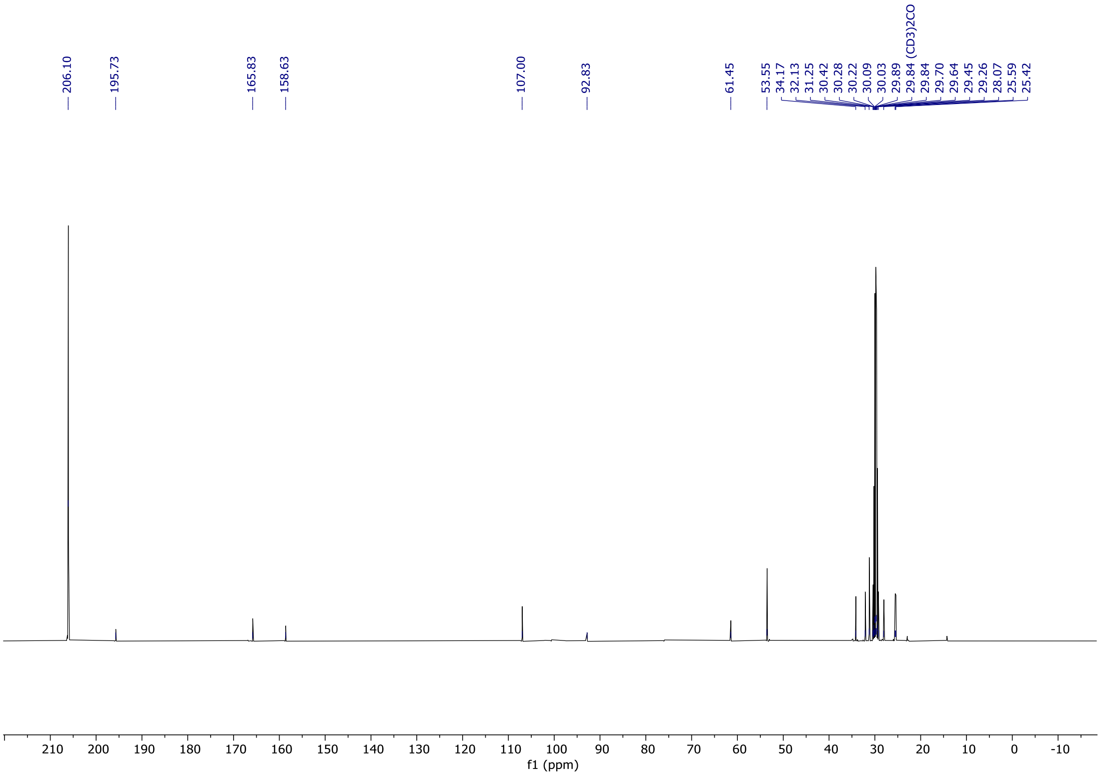

Compound 4d ^{1}H NMR (400 MHz, acetone-d_6)

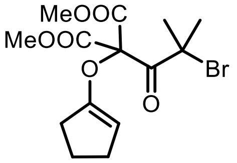

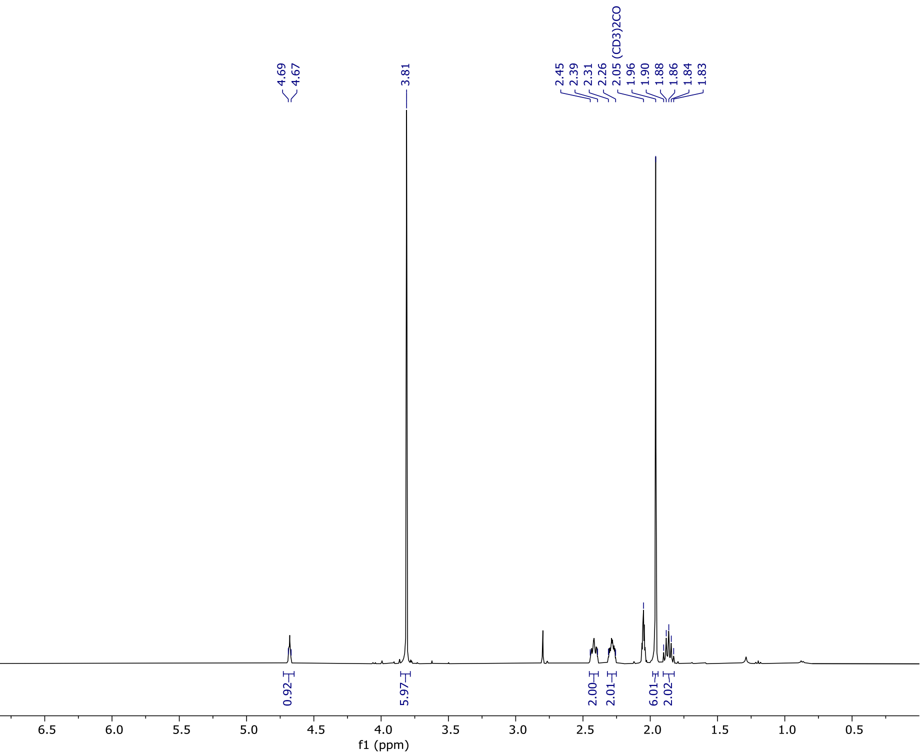

Compound 4d ^{13}C NMR (101 MHz, acetone-d_6)

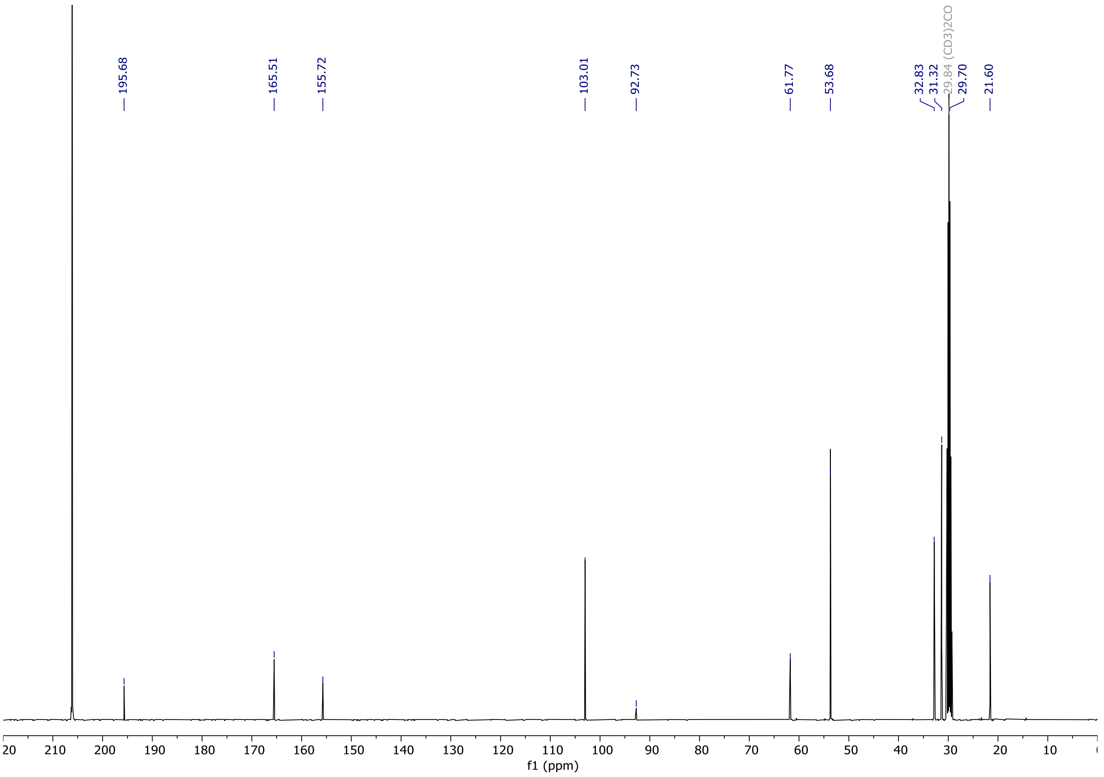

Compound 4e ^{1}H NMR (400 MHz, acetone-d_6)

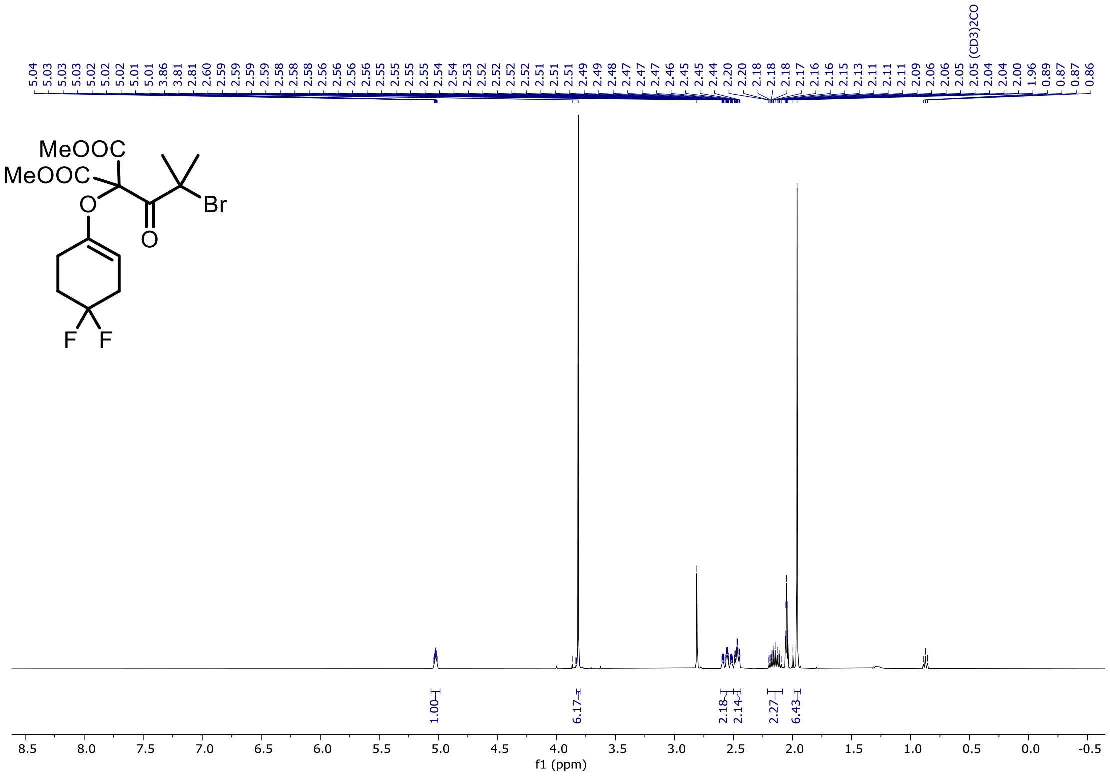

Compound 4e ^{13}C NMR (101 MHz, acetone-d_6)

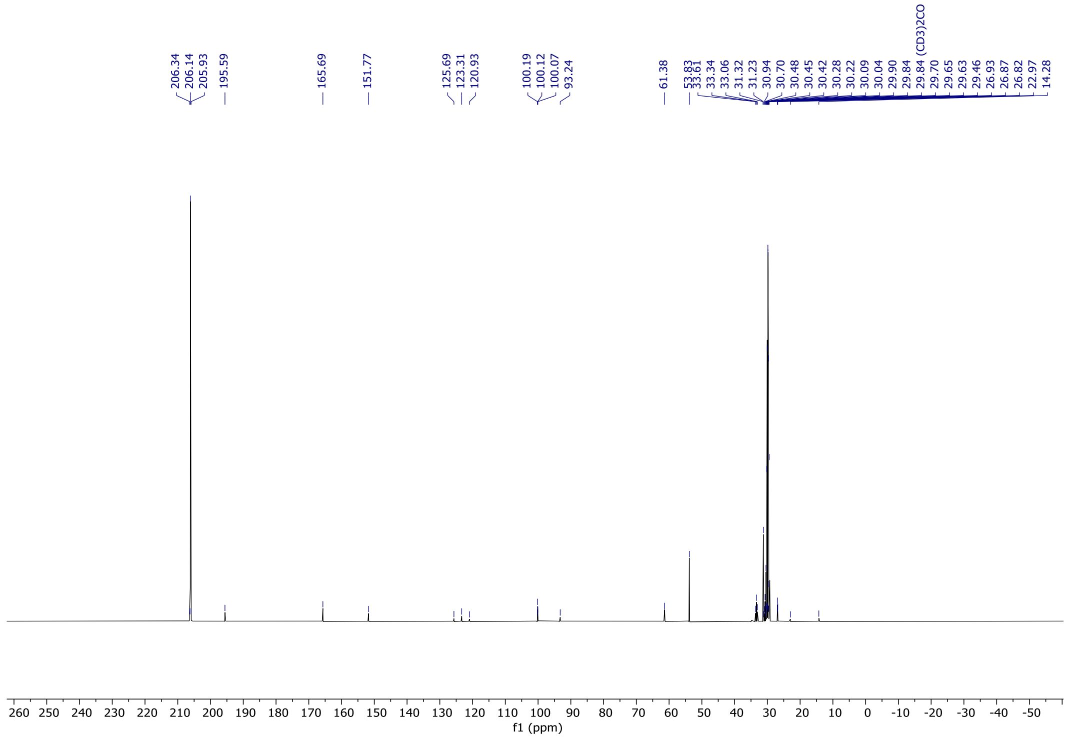

Compound 4e ^{19}F NMR (282 MHz, acetone-d_6)

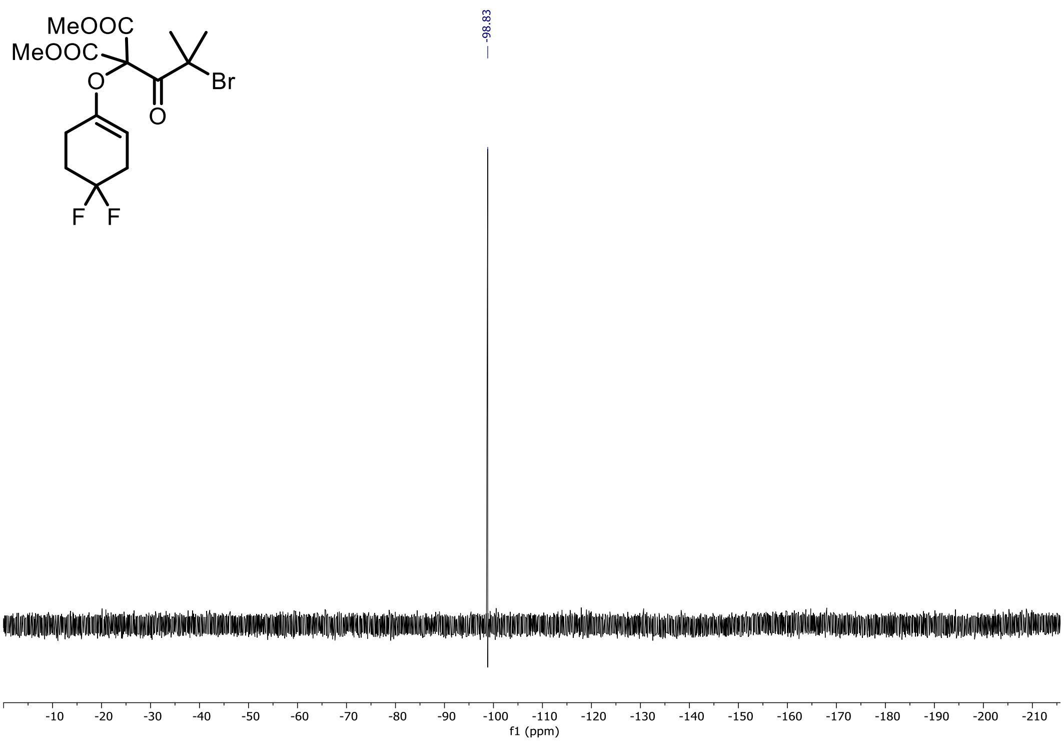

Compound 4f ^{1}H NMR (400 MHz, acetone-d_6)

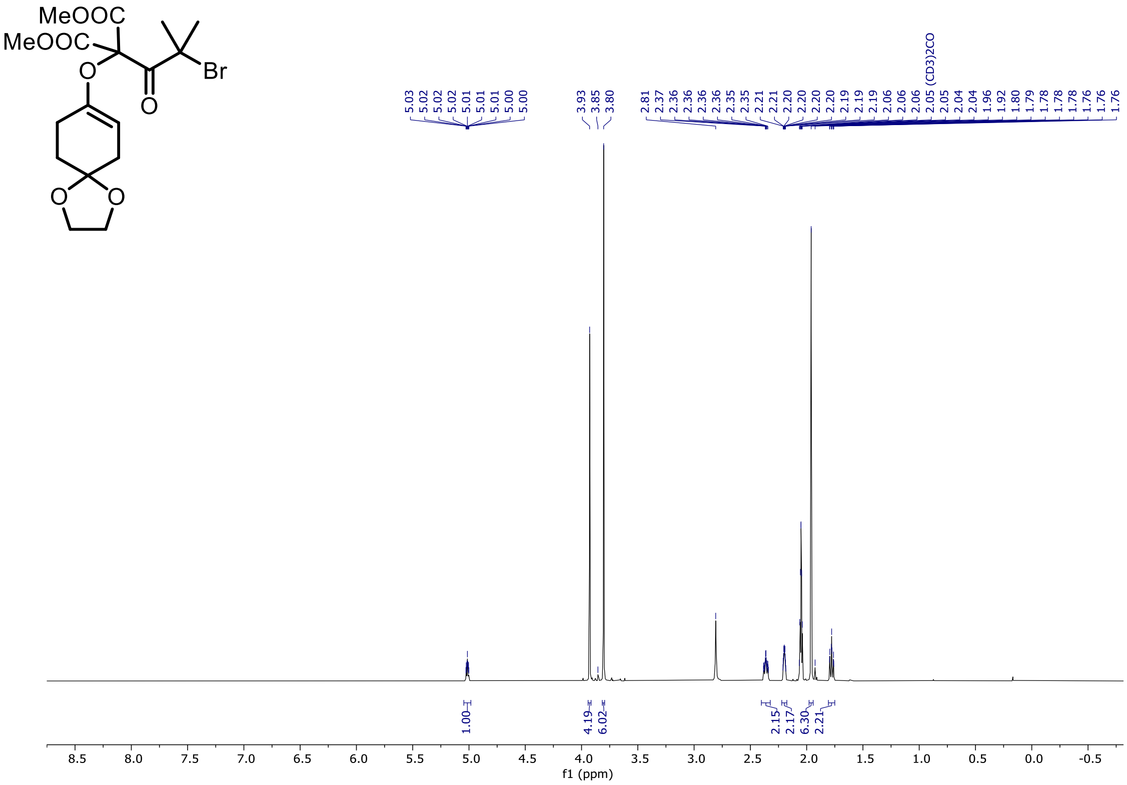

Compound 4f ^{13}C NMR (101 MHz, acetone-d_6)

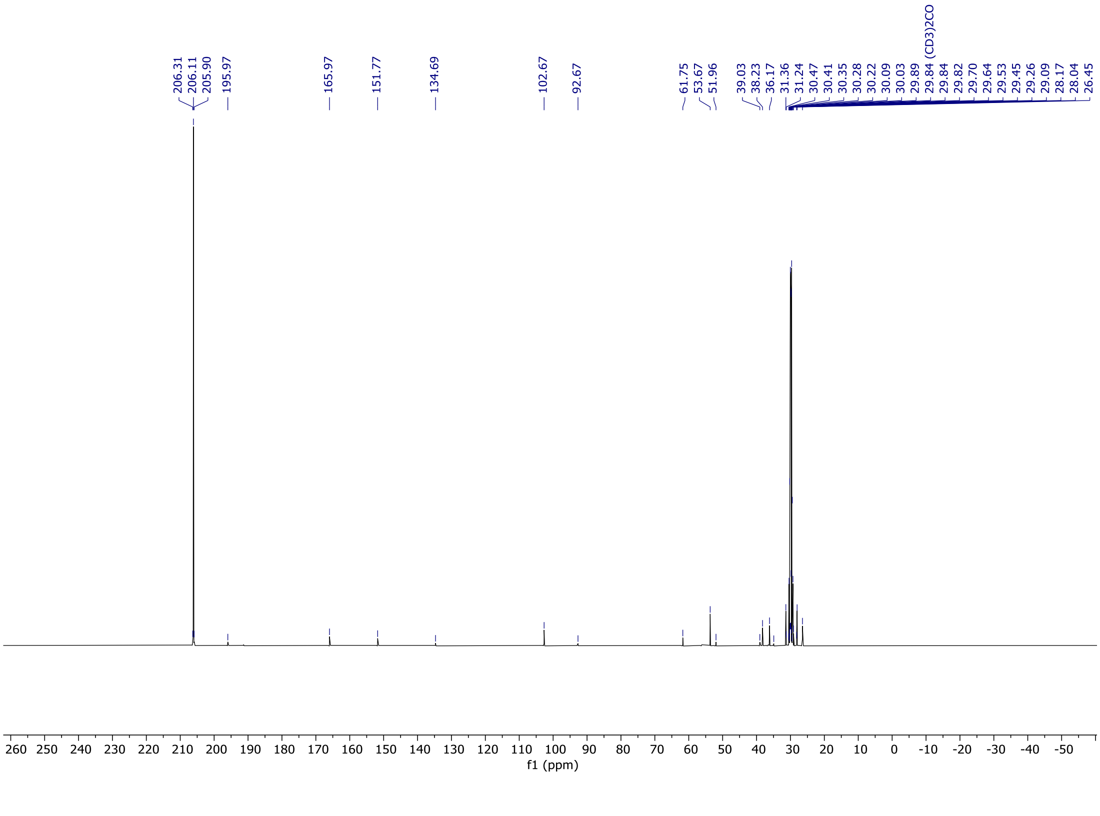

Compound 4g ^{1}H NMR (400 MHz, acetone-d_6)

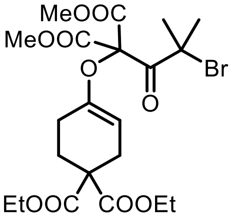

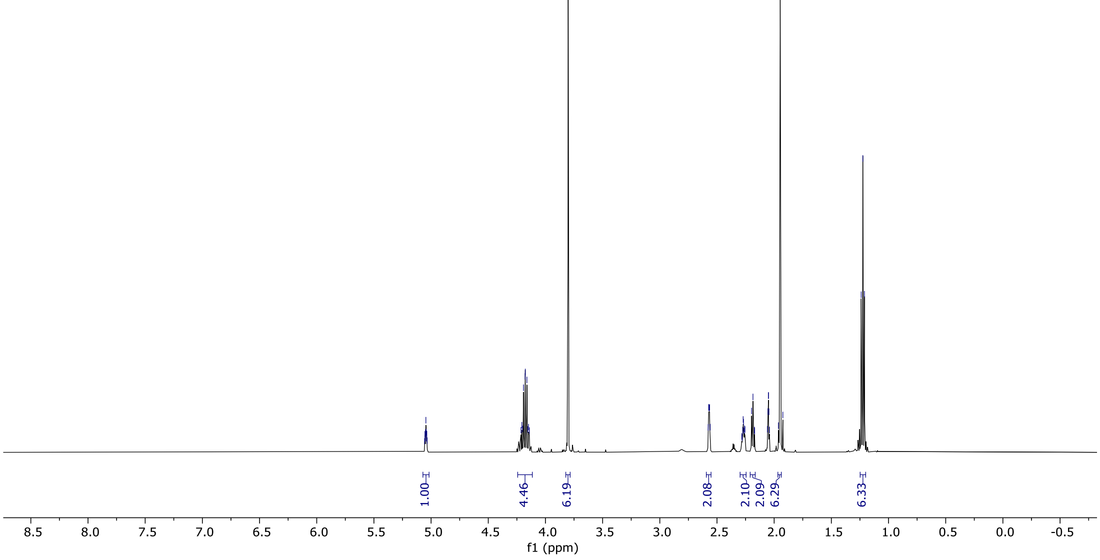

Compound 4g ^{13}C NMR (101 MHz, acetone-d_6)

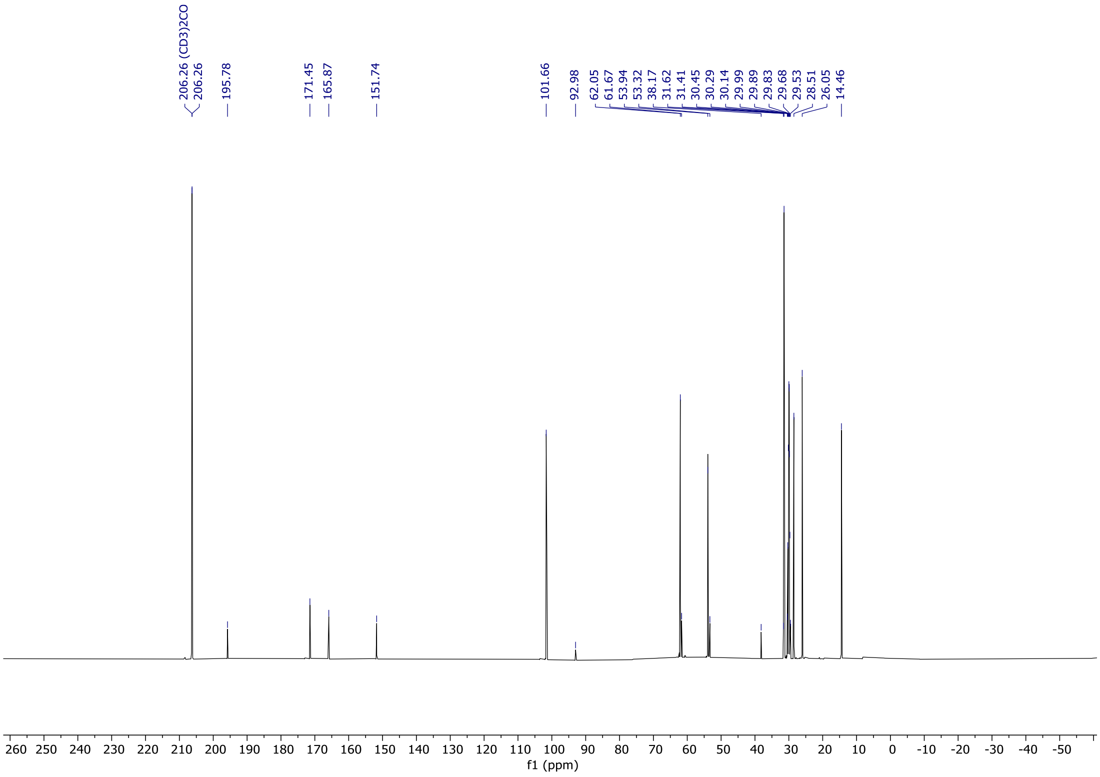

Compound 4h ^{1}H NMR (400 MHz, acetone-d_6)

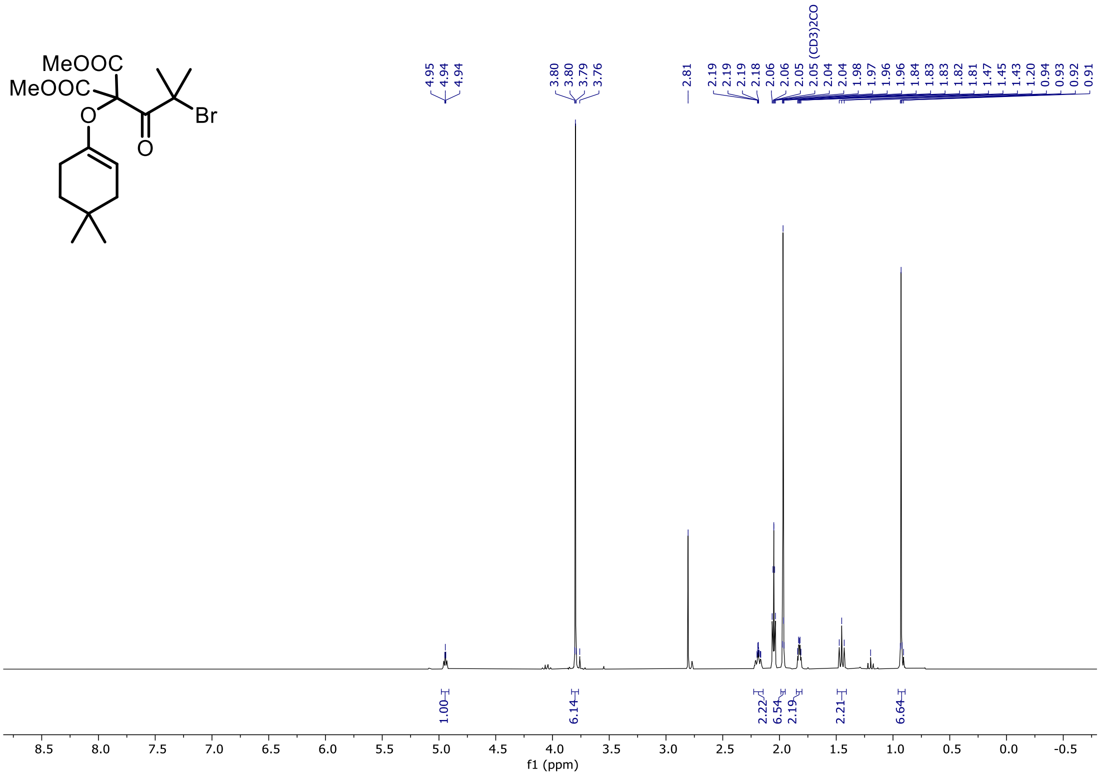

Compound 4h ^{13}C NMR (101 MHz, acetone-d_6)

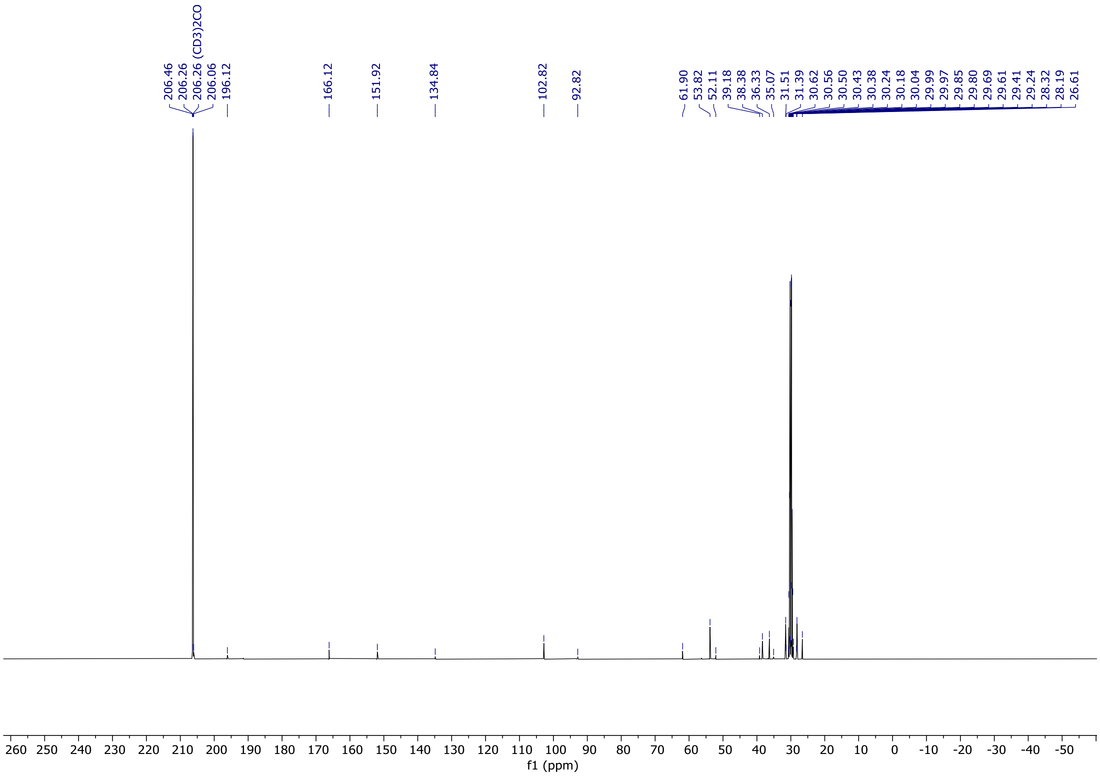

Compound 4i ¹H NMR (400 MHz, acetone-d_6)

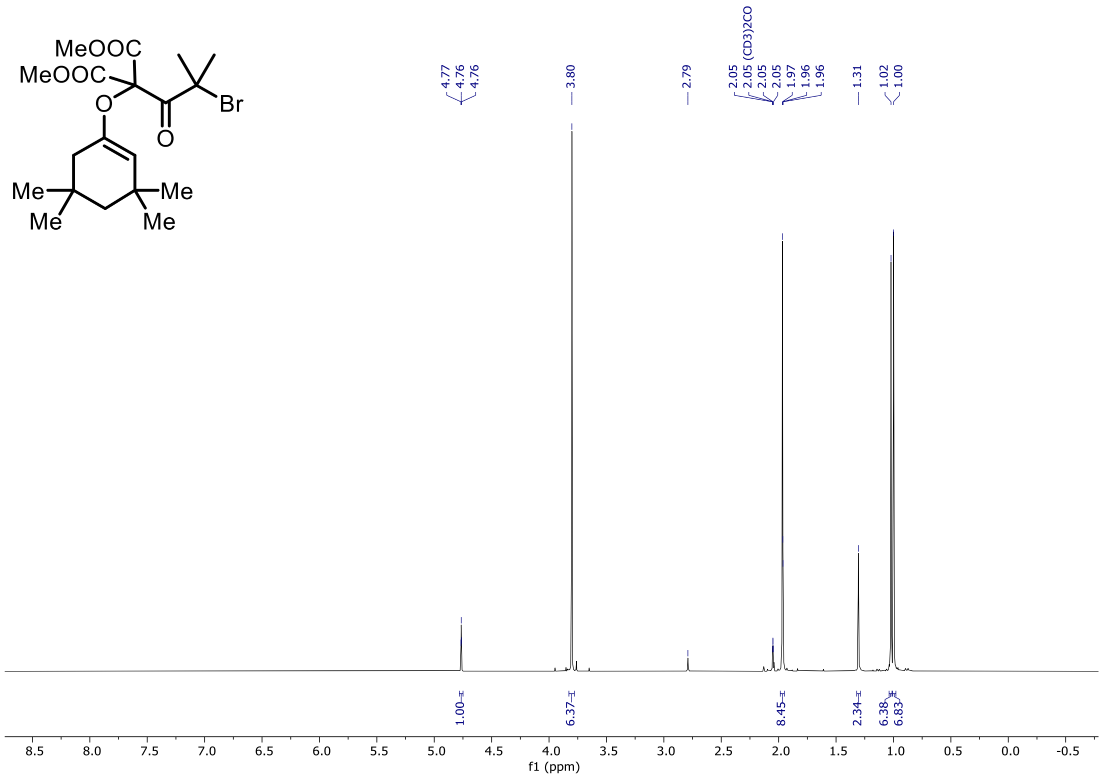

Compound 4i ¹³C NMR (101 MHz, acetone-d_6)

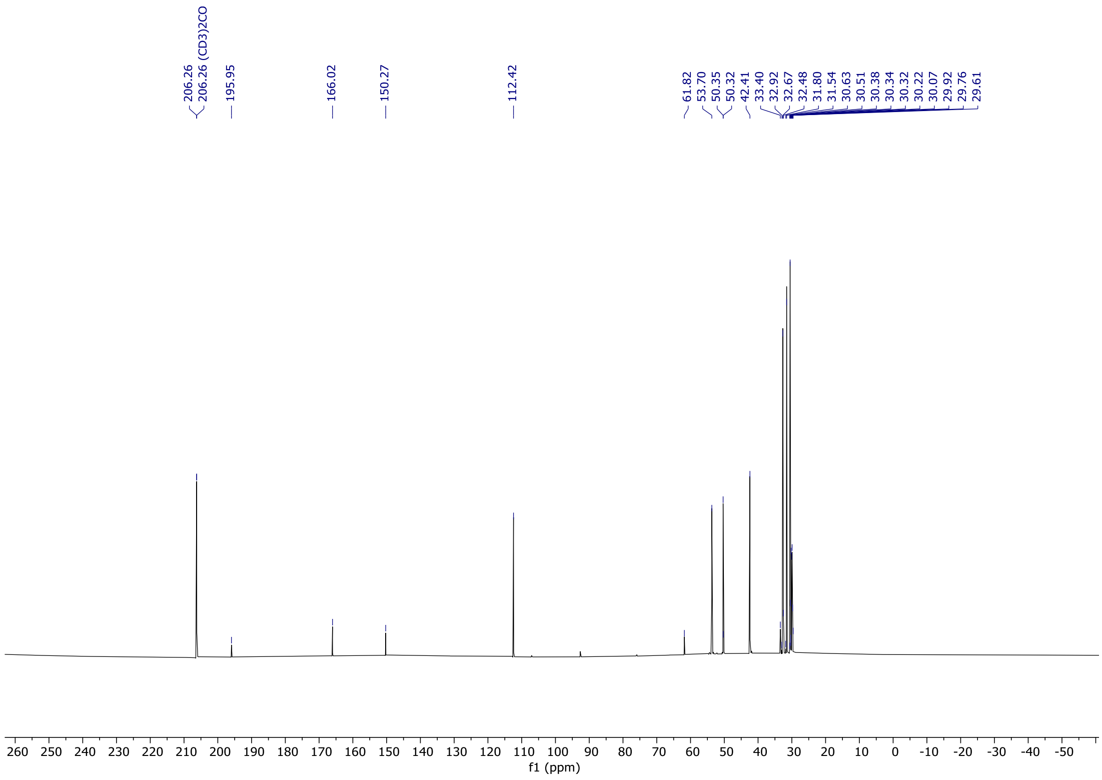

Compound 4j ^{1}H NMR (400 MHz, acetone-d_6)

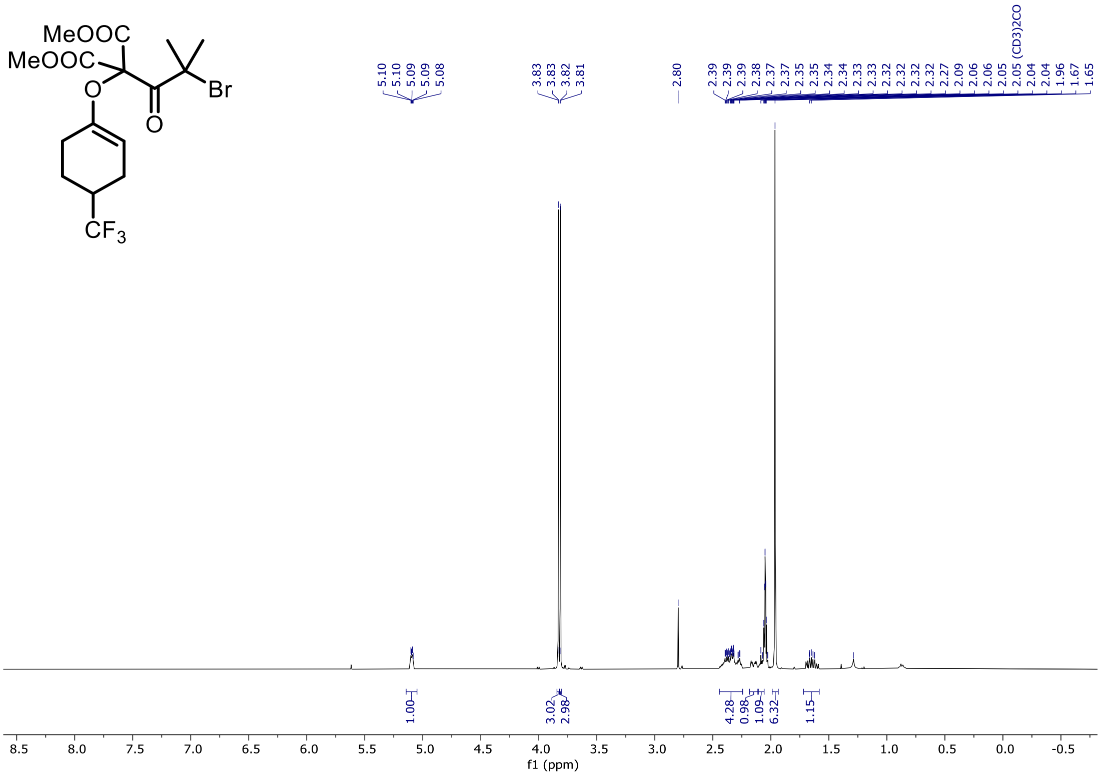

Compound 4j ^{13}C NMR (101 MHz, acetone-d_6)

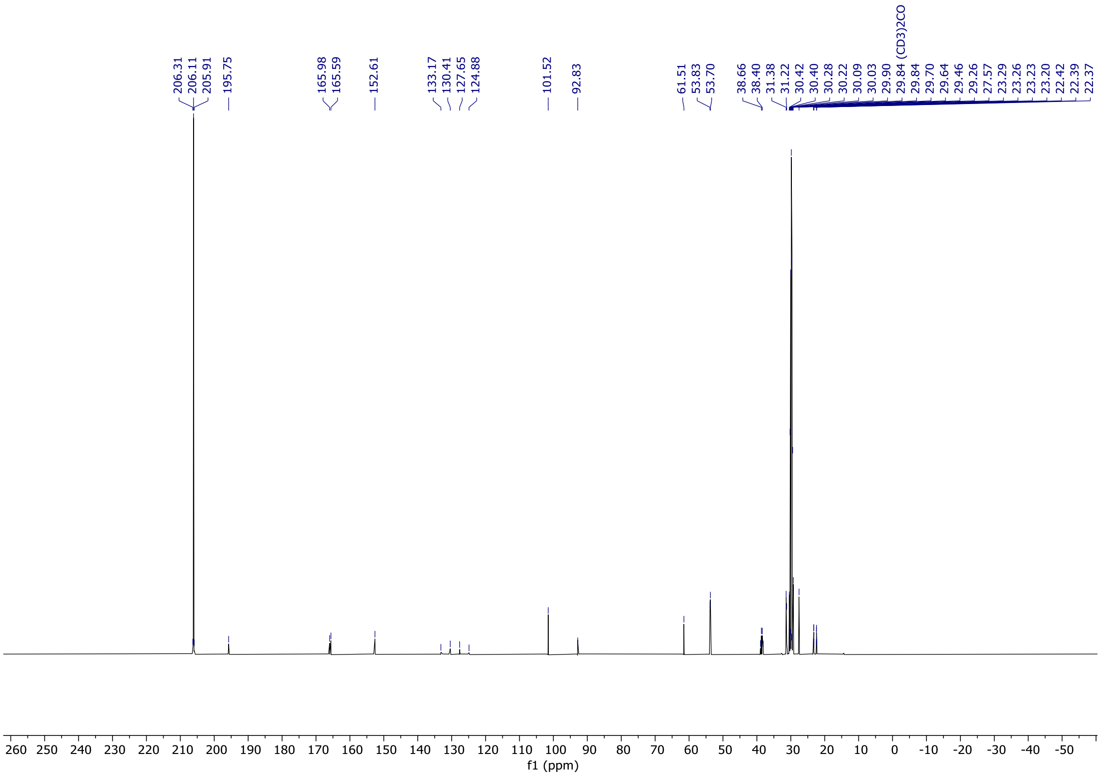

Compound 4j ^{19}F NMR (282 MHz, acetone-d_6)

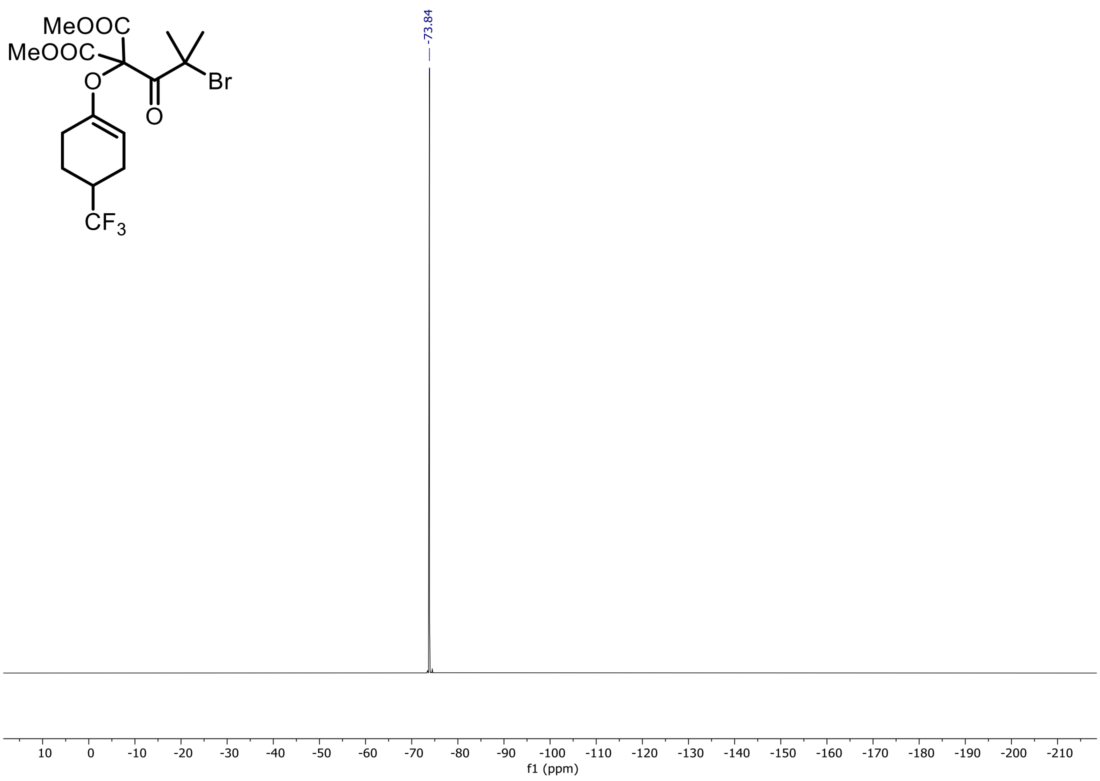

Compound 4k ^{1}H NMR (400 MHz, acetone-d_6)

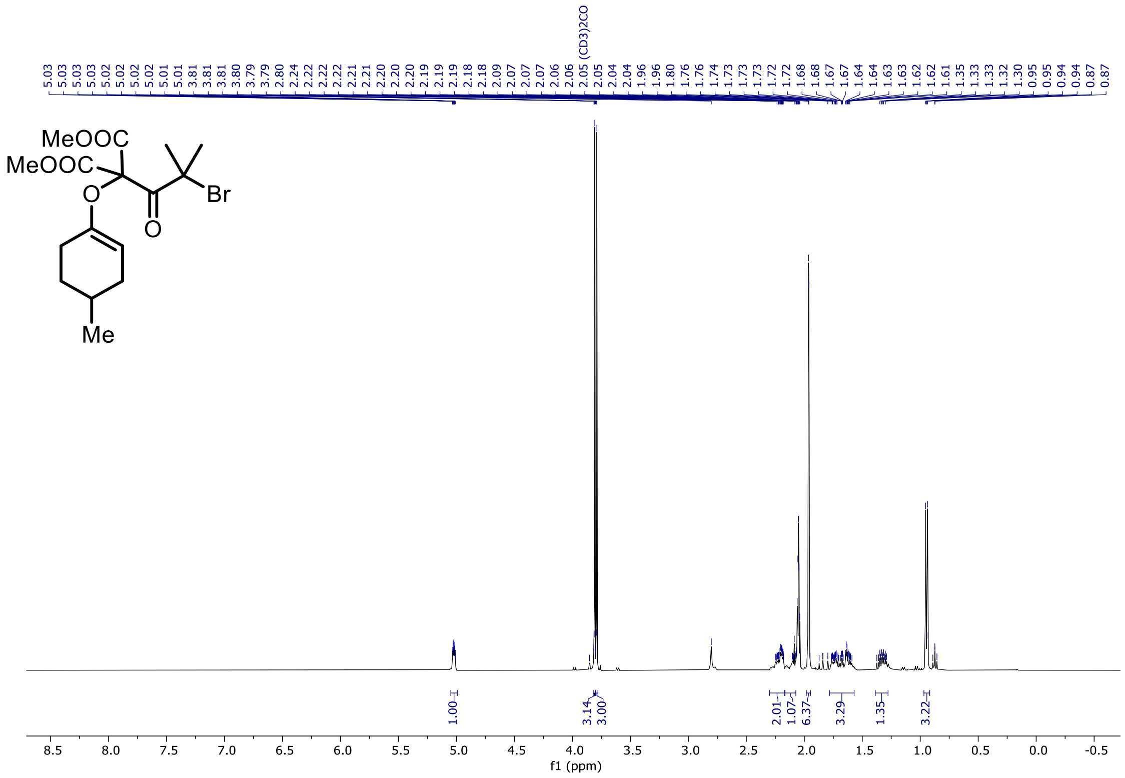

Compound 4k ^{13}C NMR (101 MHz, acetone-d_6)

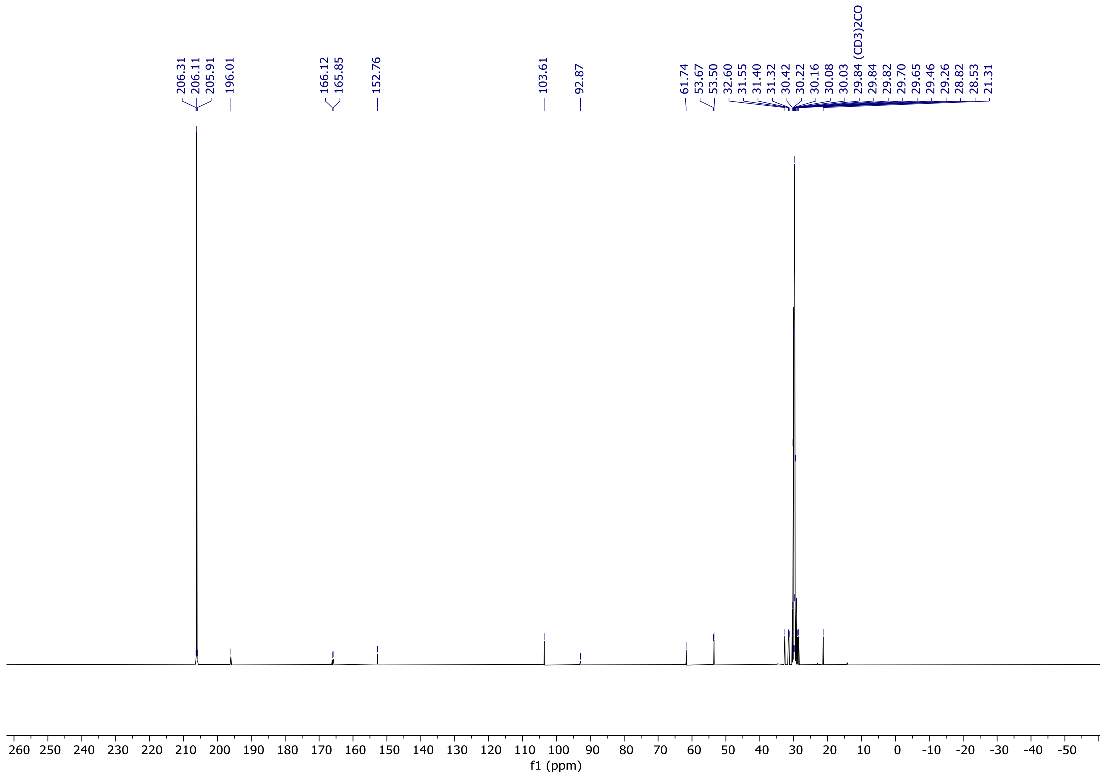

Compound 4l ^{1}H NMR (400 MHz, $CDCl_3$)

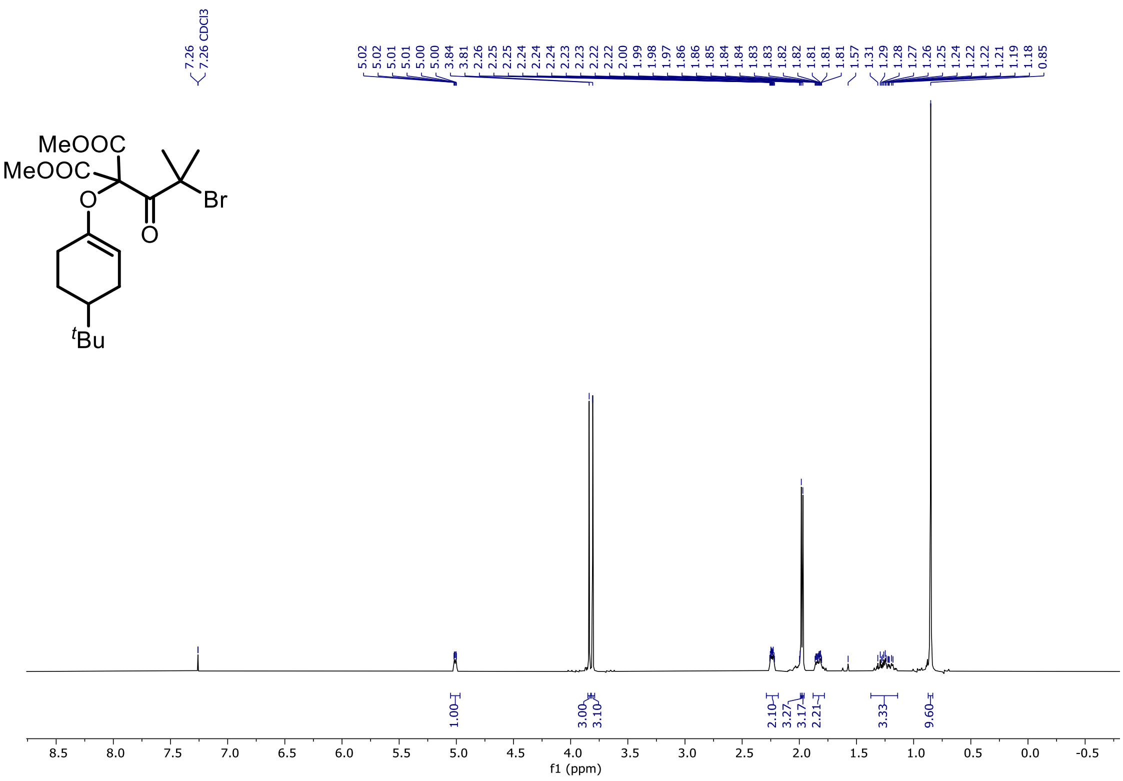

Compound 4l ^{13}C NMR (101 MHz, $CDCl_3$)

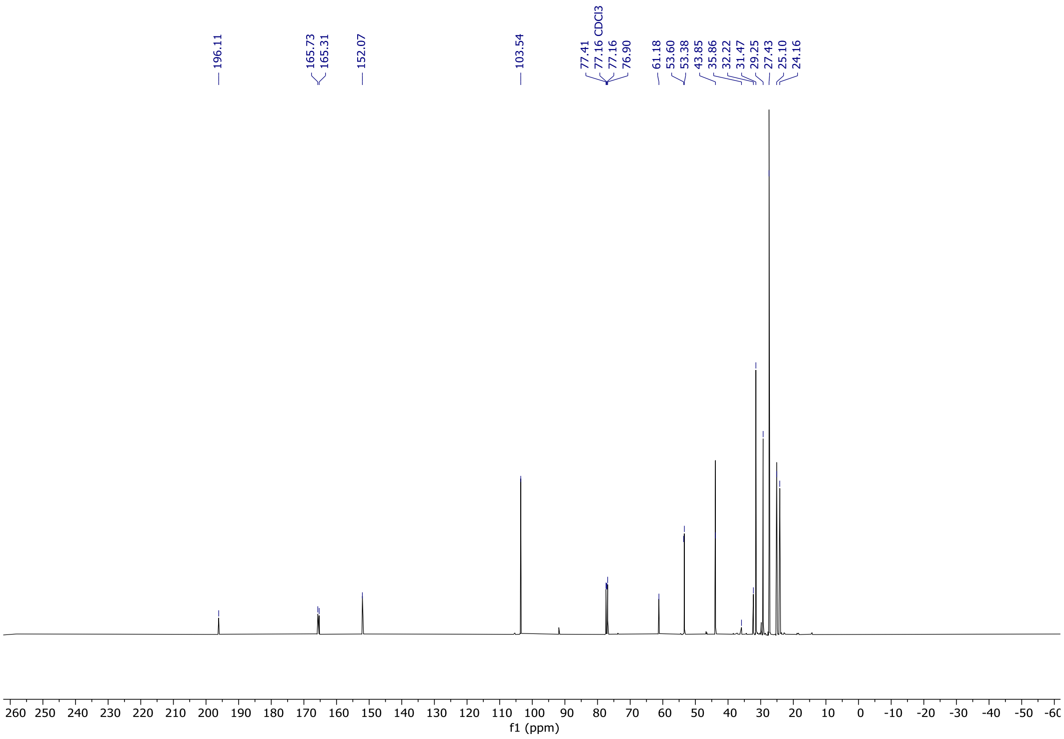

Compound 4m ¹H NMR (400 MHz, acetone-d_6)

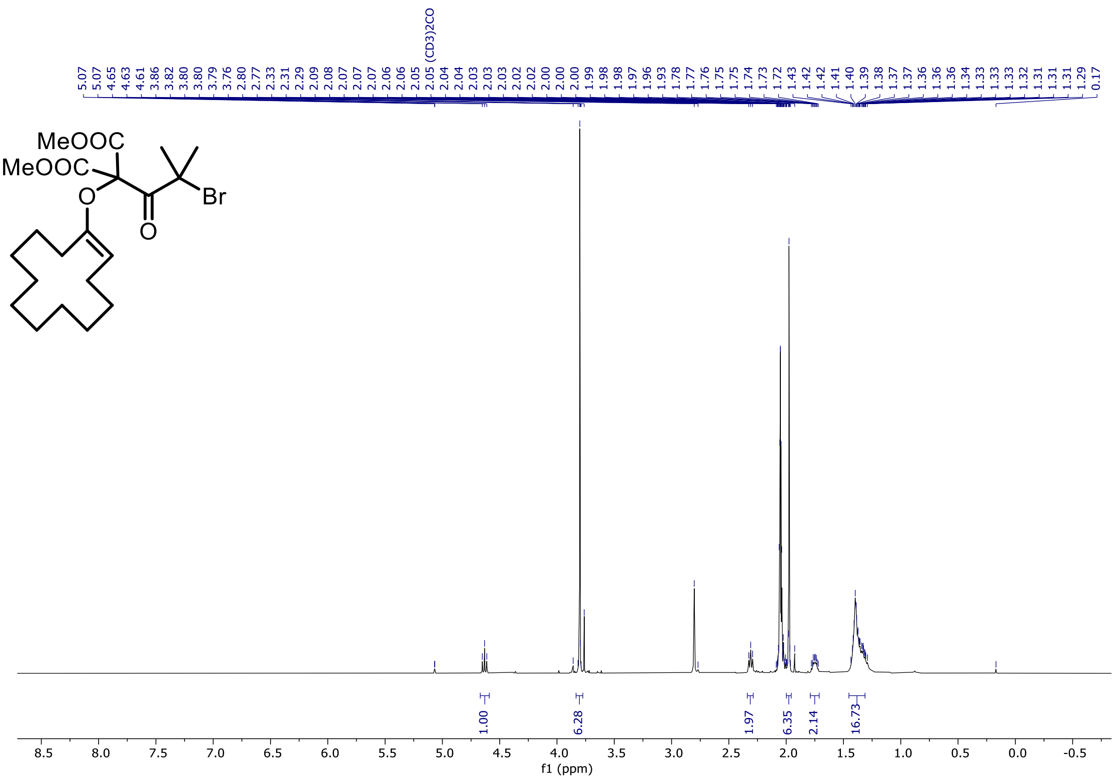

Compound 4m ¹³C NMR (101 MHz, acetone-d_6)

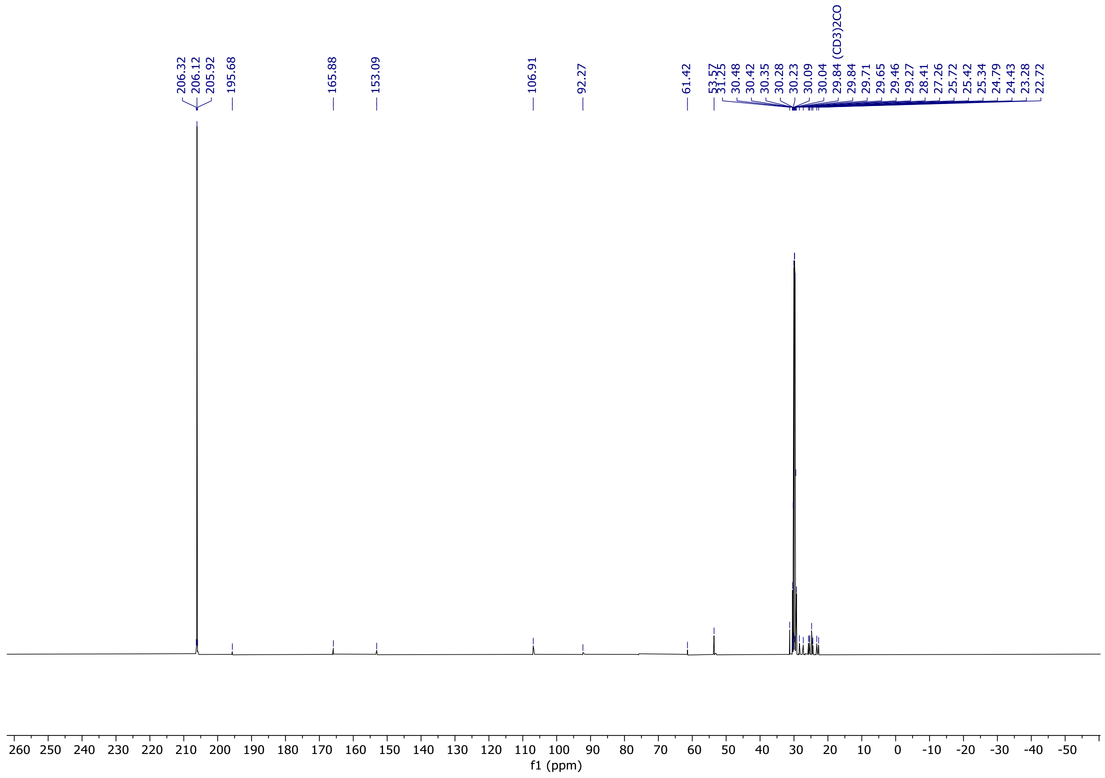

Compound 5a ^{1}H NMR (400 MHz, $CDCl_3$)

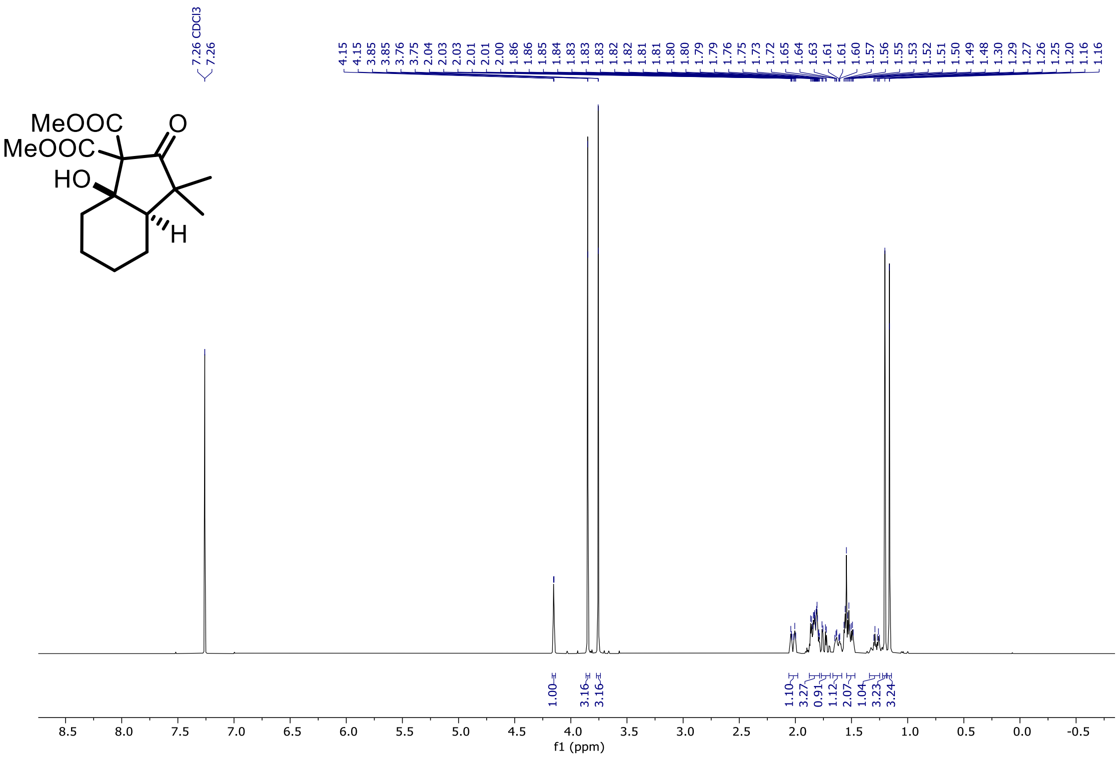

Compound 5a ^{13}C NMR (101 MHz, $CDCl_3$)

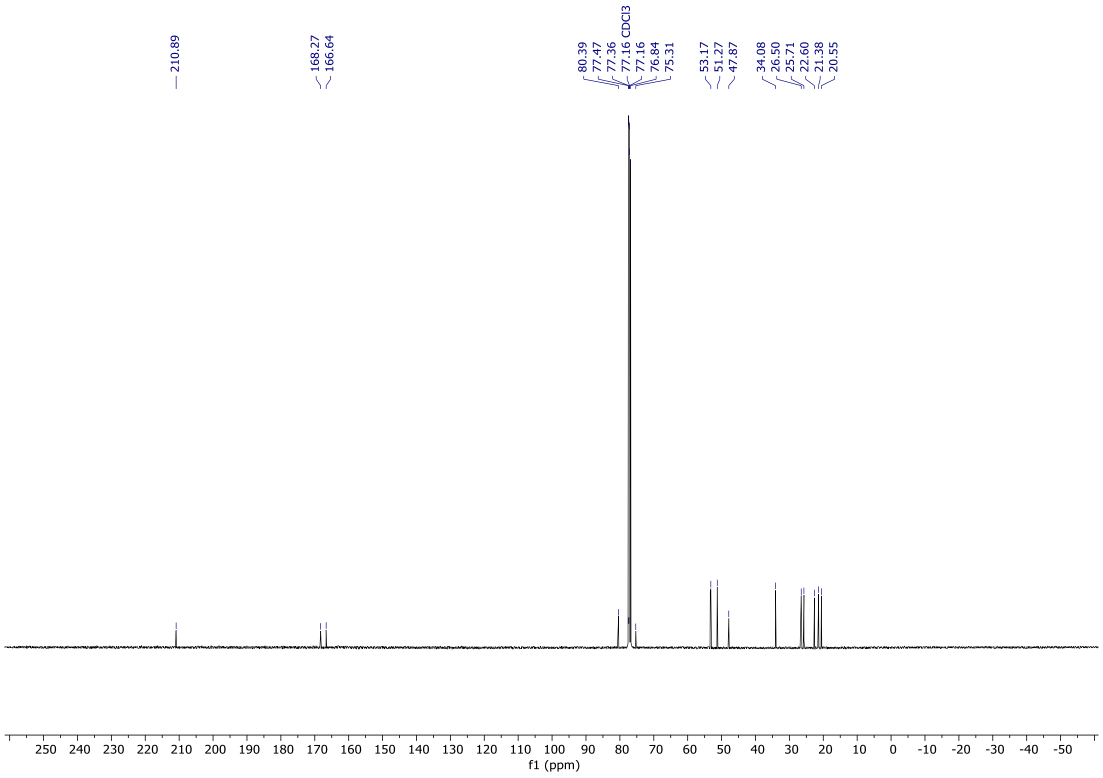

Compound 5b ^{1}H NMR (400 MHz, $CDCl_3$)

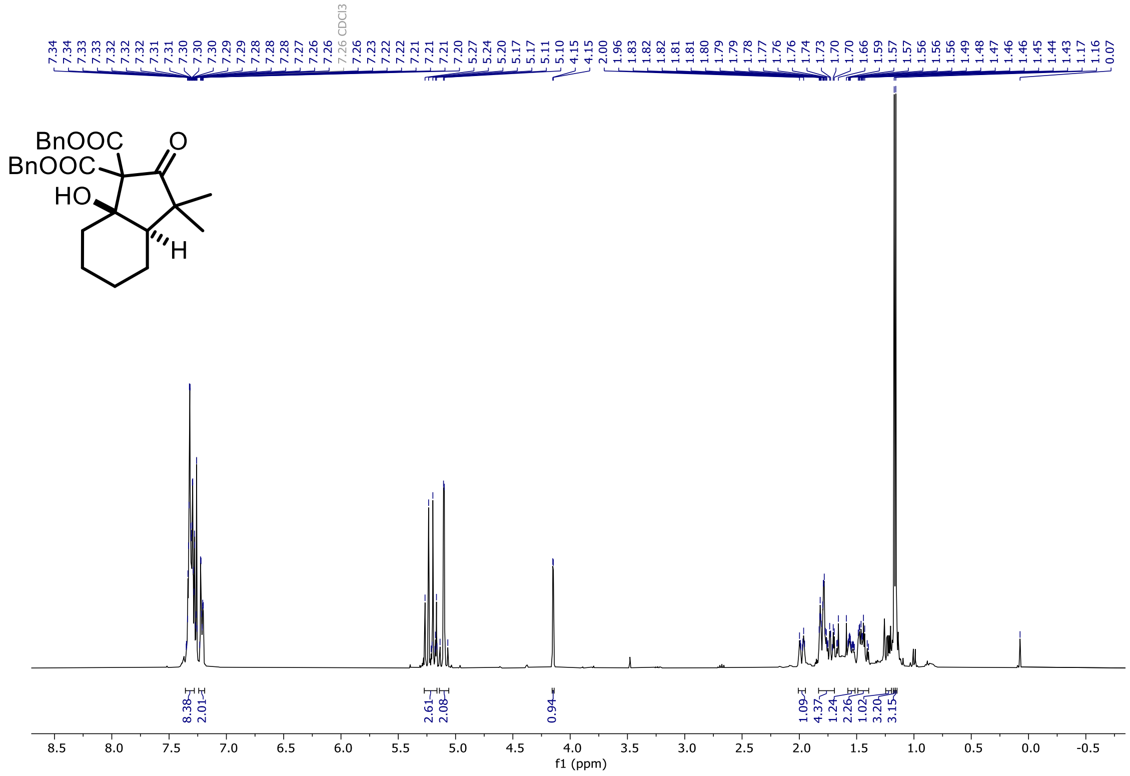

Compound 5b ^{13}C NMR (101 MHz, $CDCl_3$)

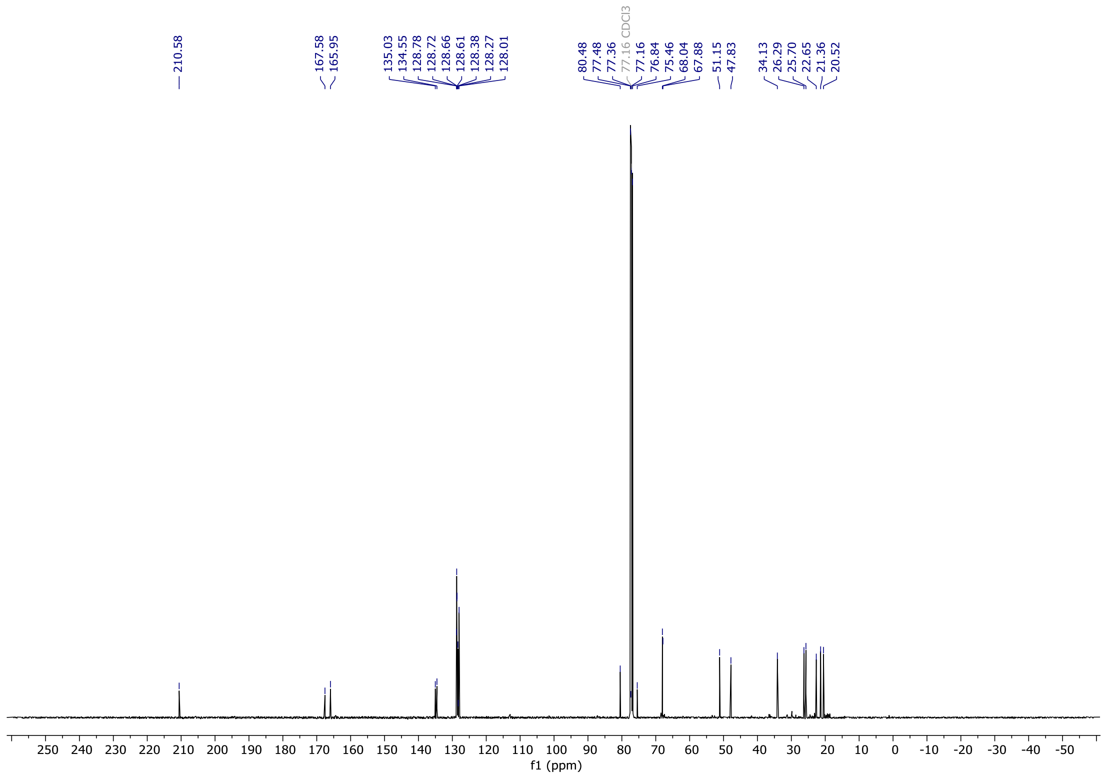

Compound 5c ^{1}H NMR (400 MHz, $CDCl_3$)

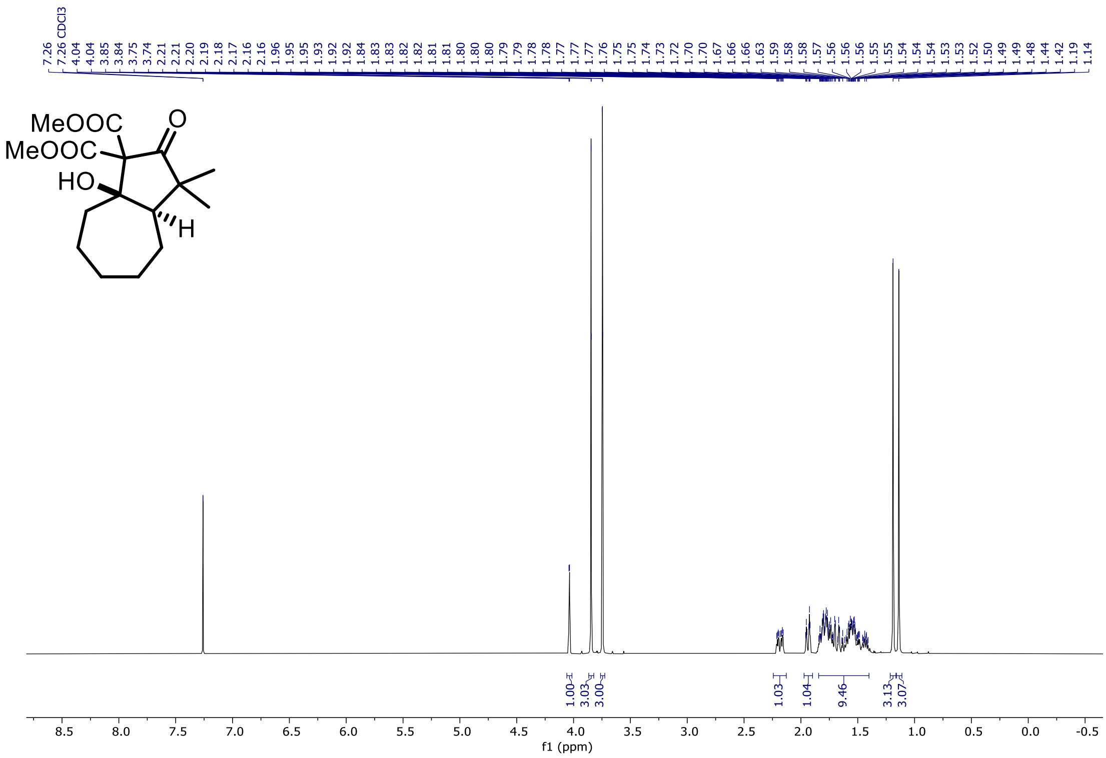

Compound 5c ^{13}C NMR (101 MHz, $CDCl_3$)

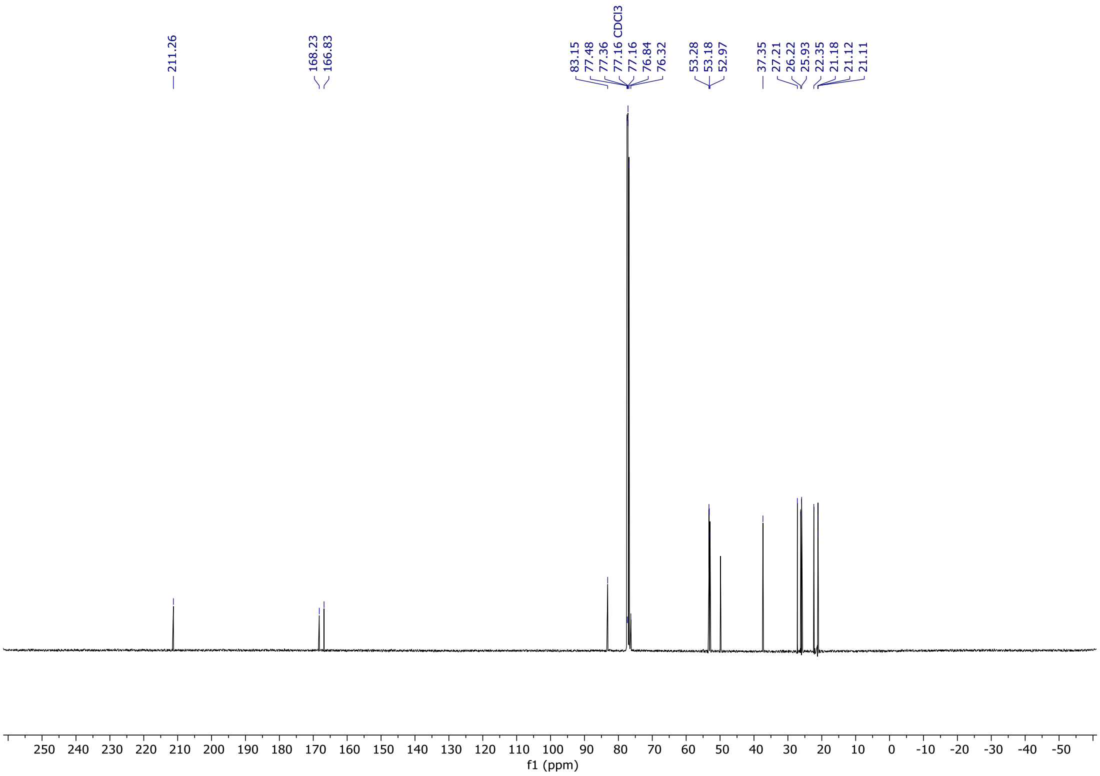

Compound 5e ^{1}H NMR (400 MHz, $CDCl_3$)

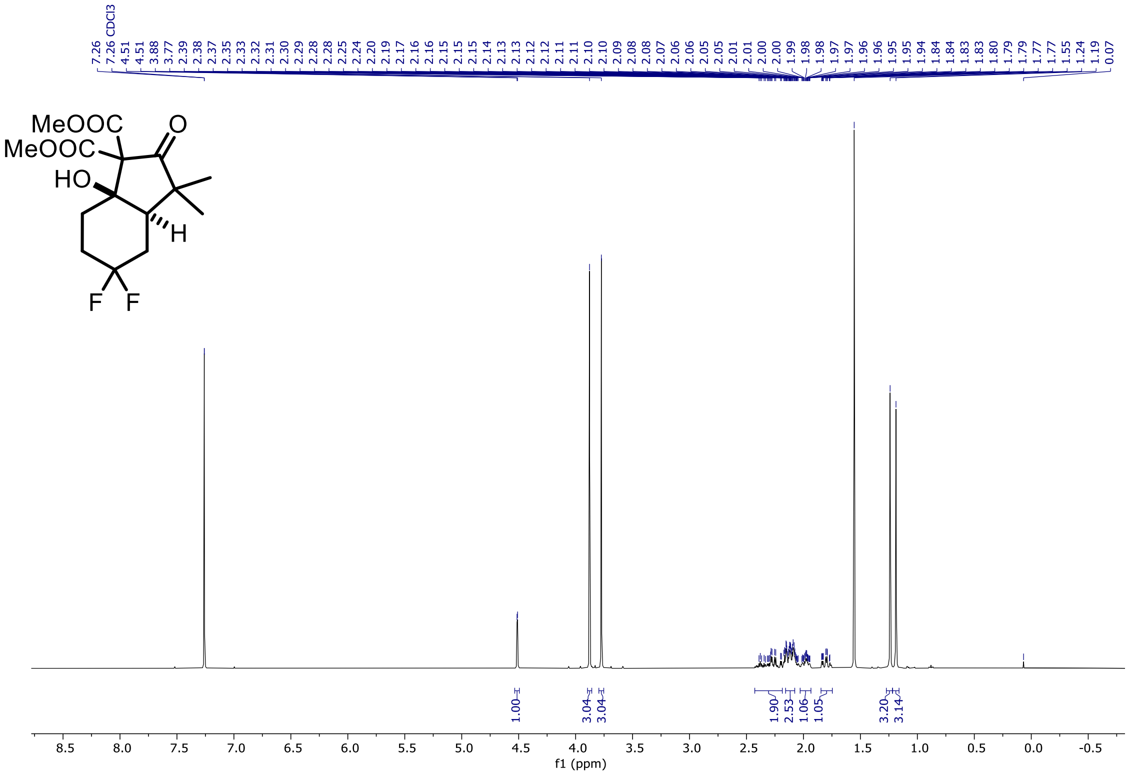

Compound 5e ^{13}C NMR (101 MHz, $CDCl_3$)

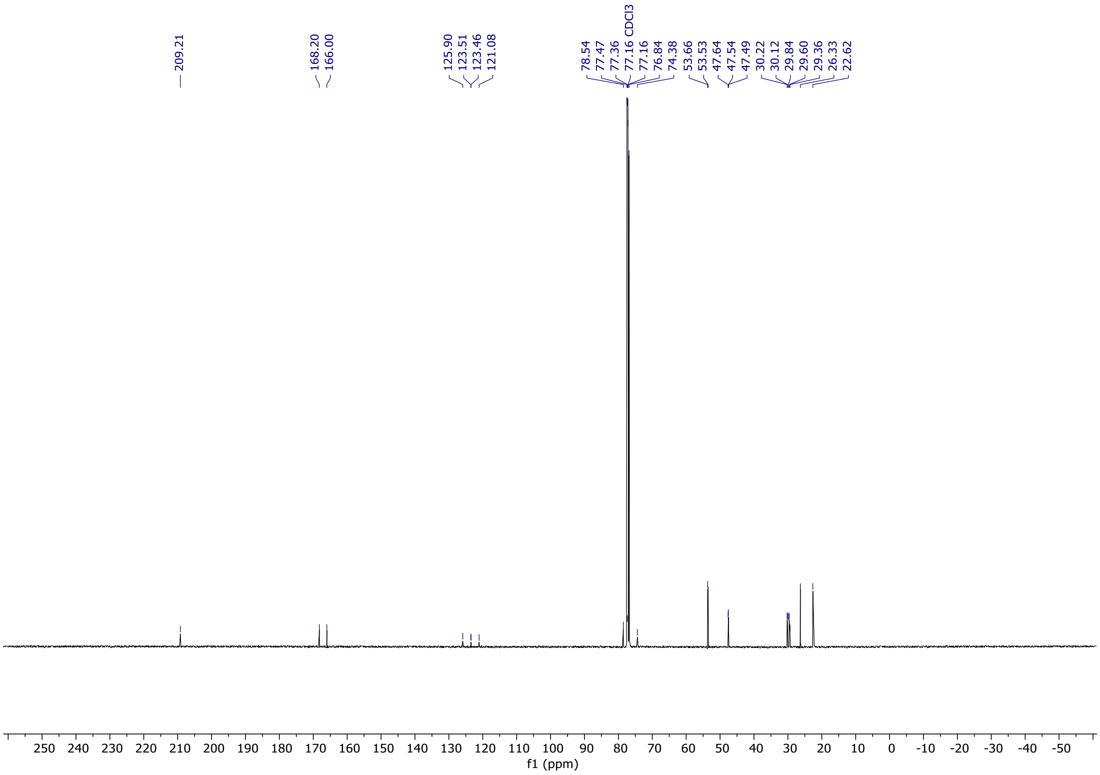

Compound 5e ^{19}F NMR (282 MHz, $CDCl_3$)

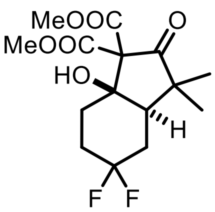

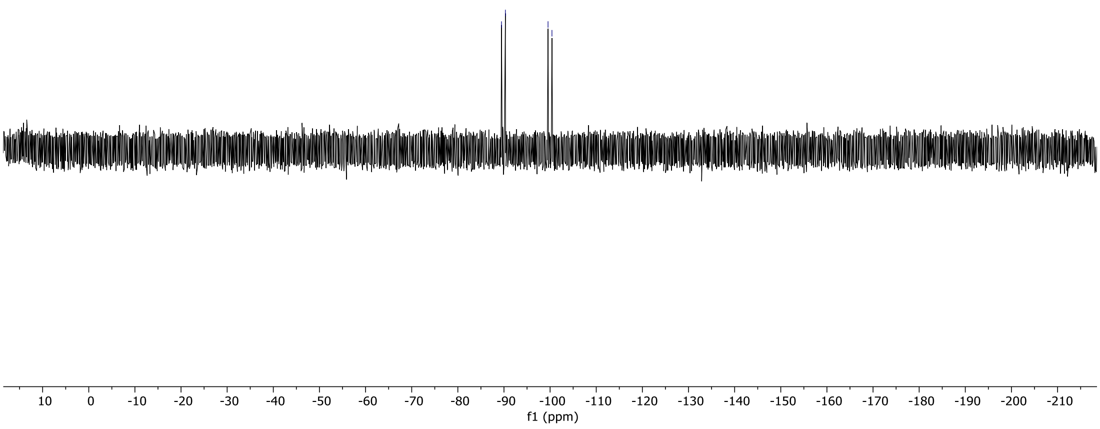

Compound 5f ^{1}H NMR (400 MHz, $CDCl_3$)

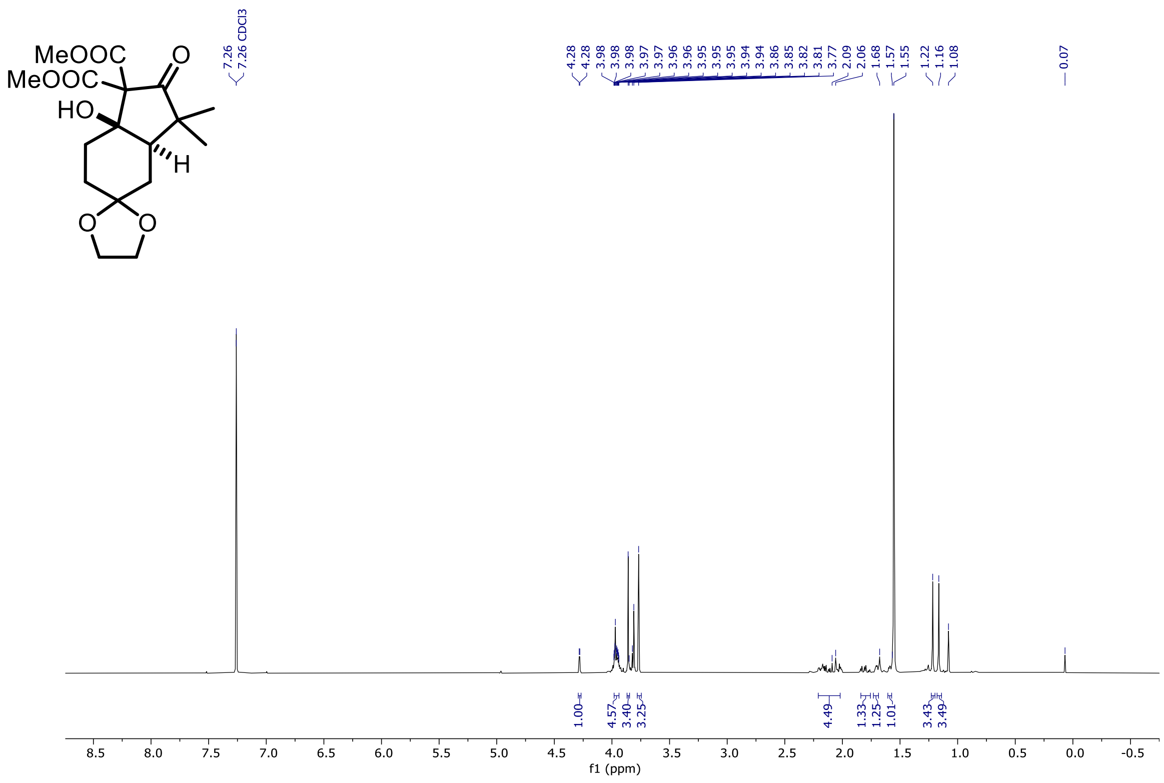

Compound 5f ^{13}C NMR (101 MHz, $CDCl_3$)

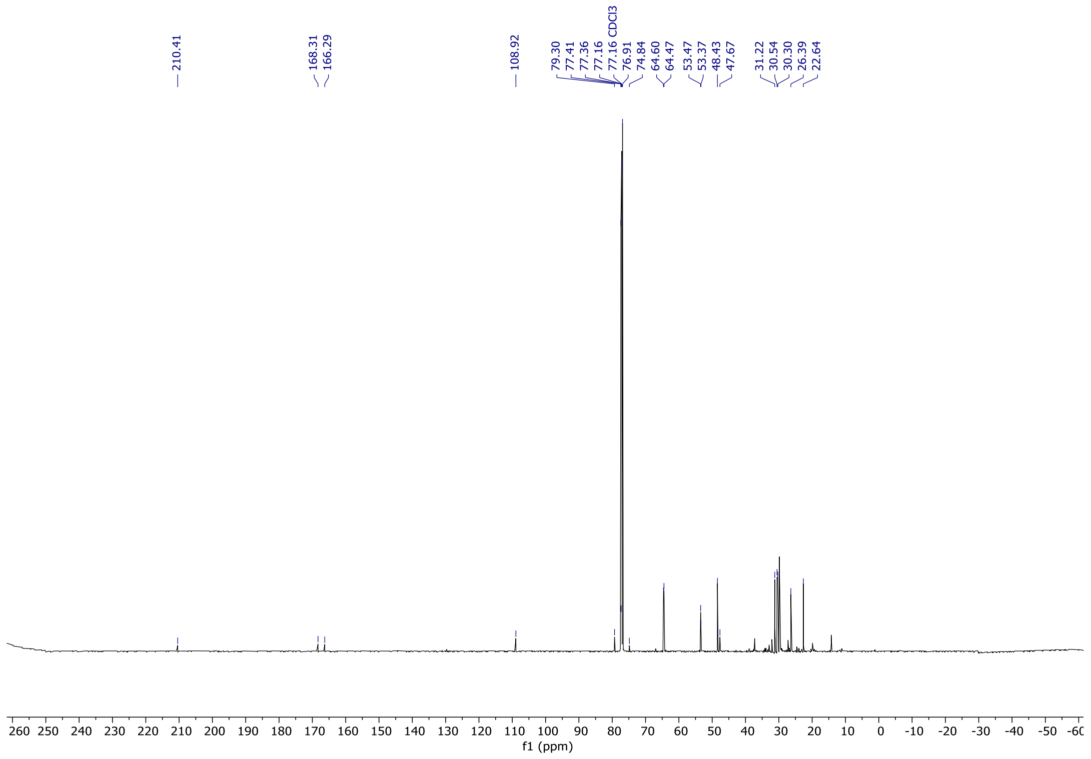

Compound 5j ^{1}H NMR (400 MHz, $CDCl_3$)

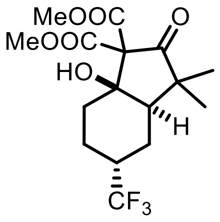

maj

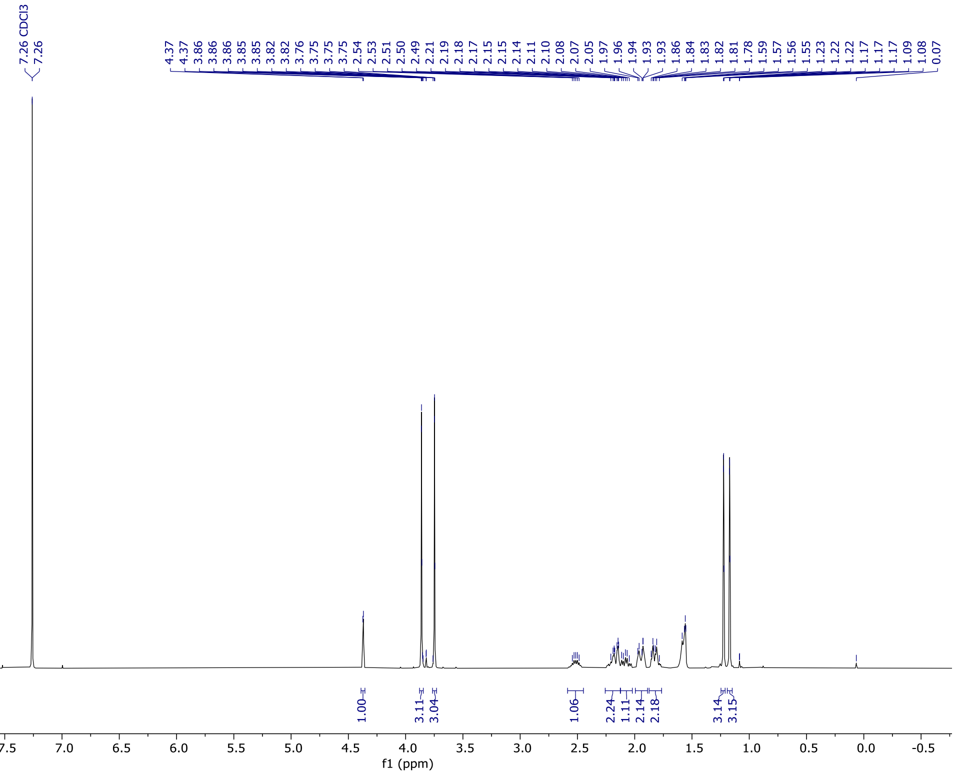

Compound 5j ^{13}C NMR (101 MHz, $CDCl_3$)

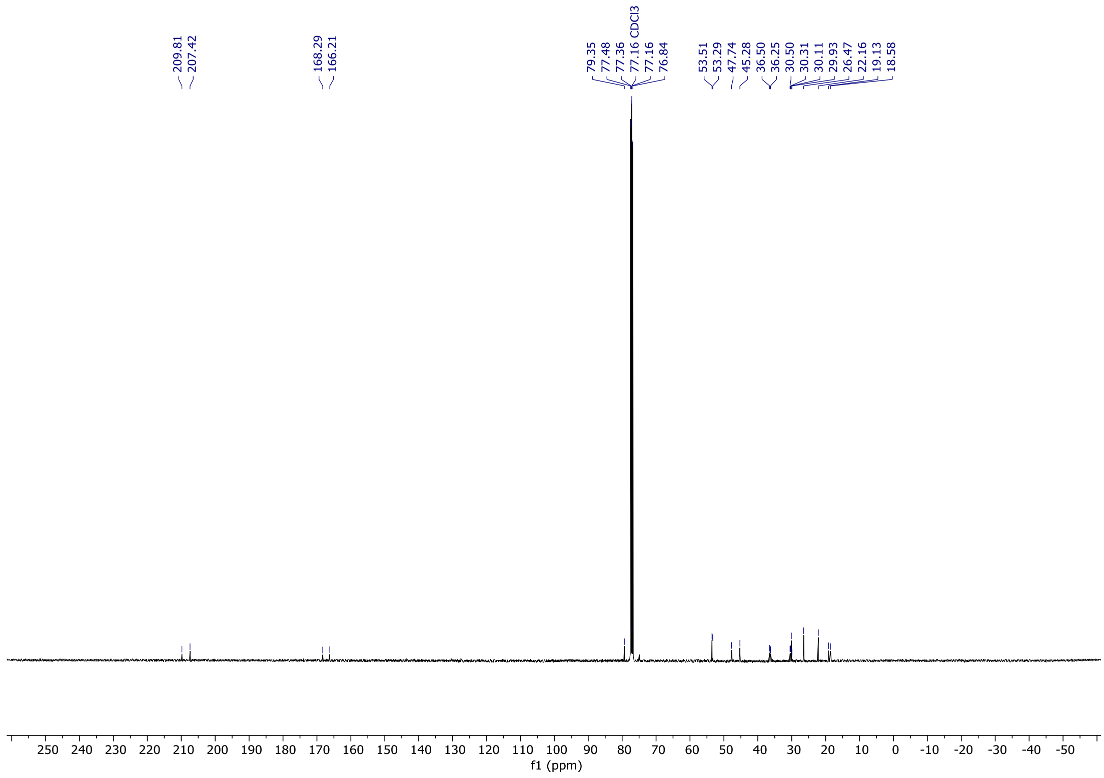

Compound 5j ^{19}F NMR (282 MHz, $CDCl_3$)

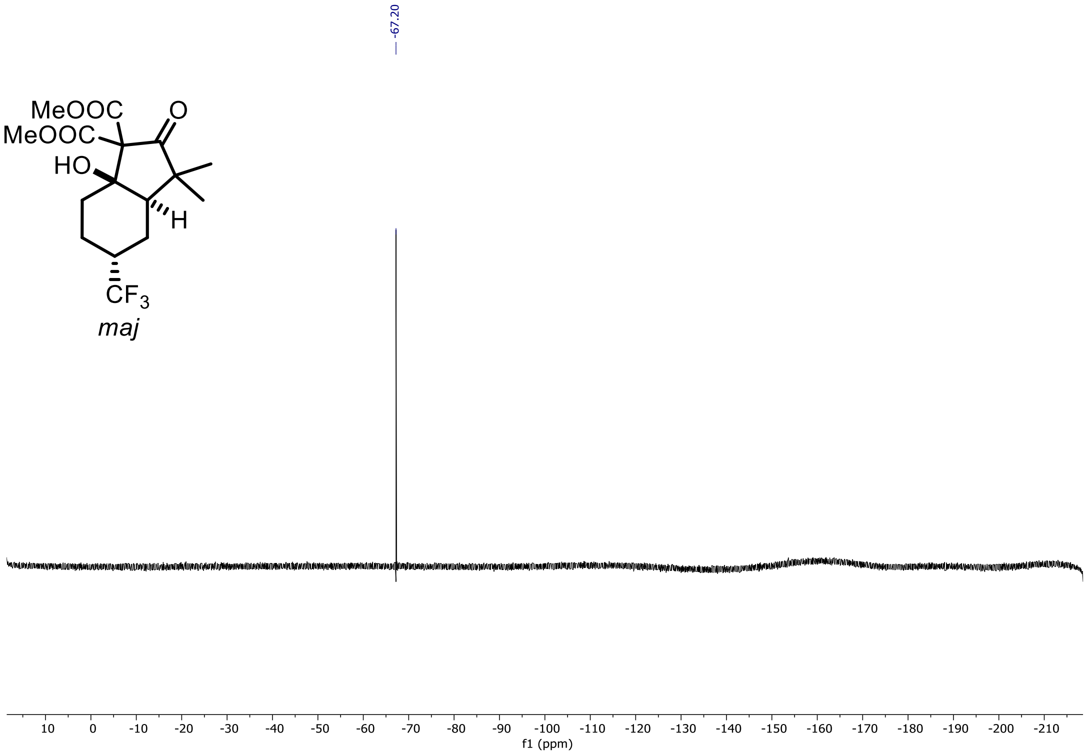

Compound 5k ^{1}H NMR (400 MHz, $CDCl_3$)

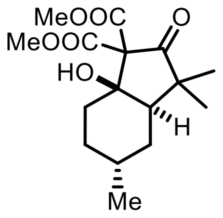

maj

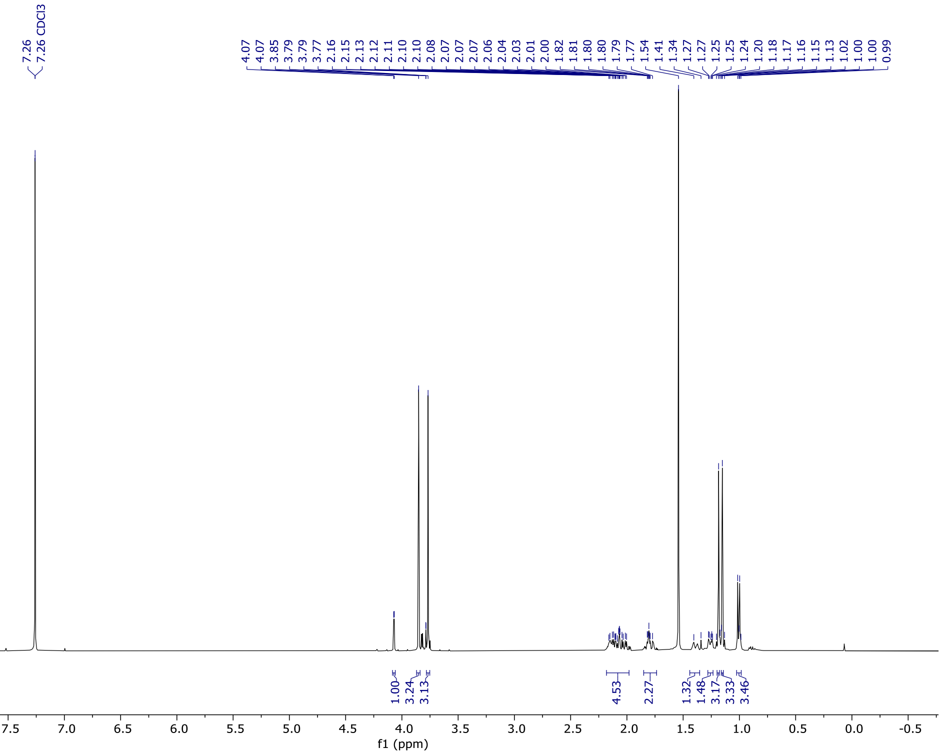

Compound 5k ^{13}C NMR (101 MHz, $CDCl_3$)

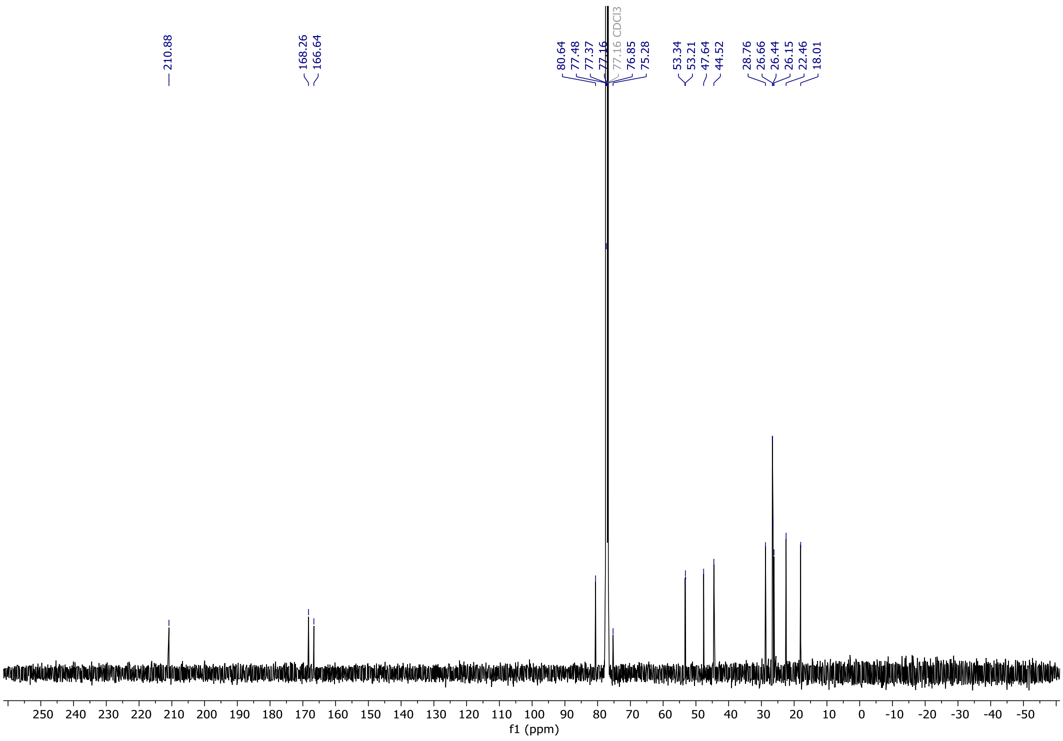

Mixture of 5m and 6m ^{1}H NMR (400 MHz, $CDCl_3$)

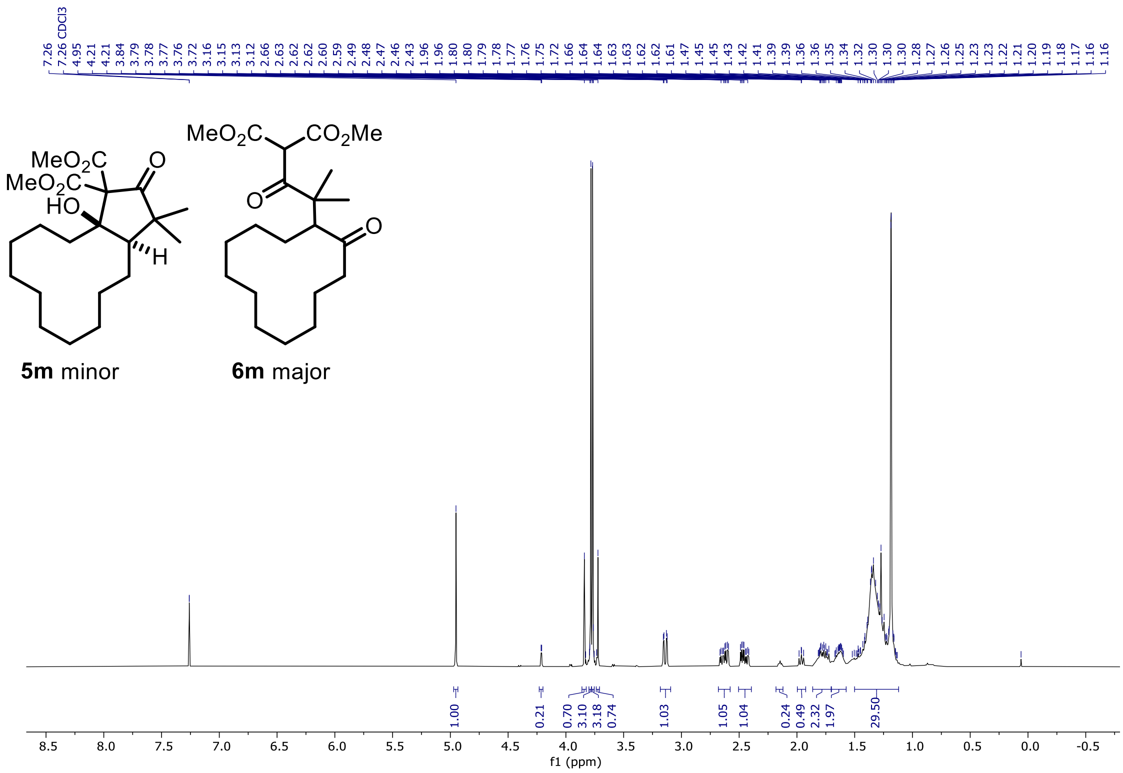

Mixture of 5m and 6m ^{13}C NMR (101 MHz, $CDCl_3$)

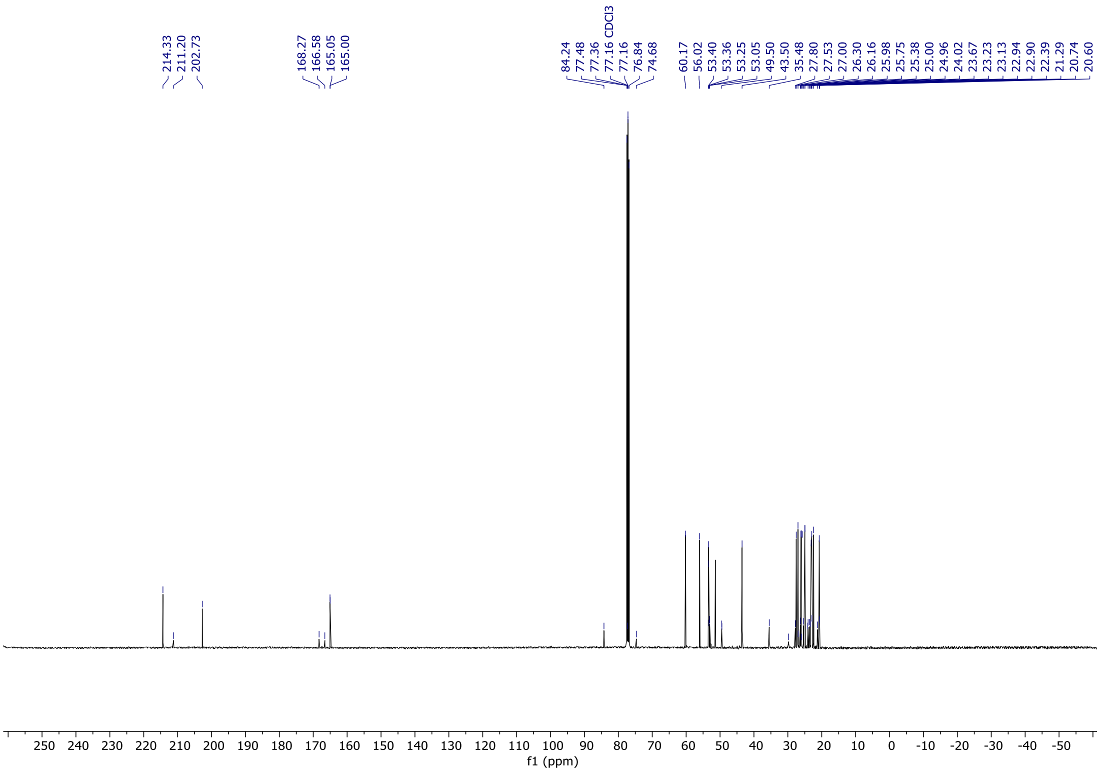

Compound 7i ^{1}H NMR (400 MHz, $CDCl_3$)

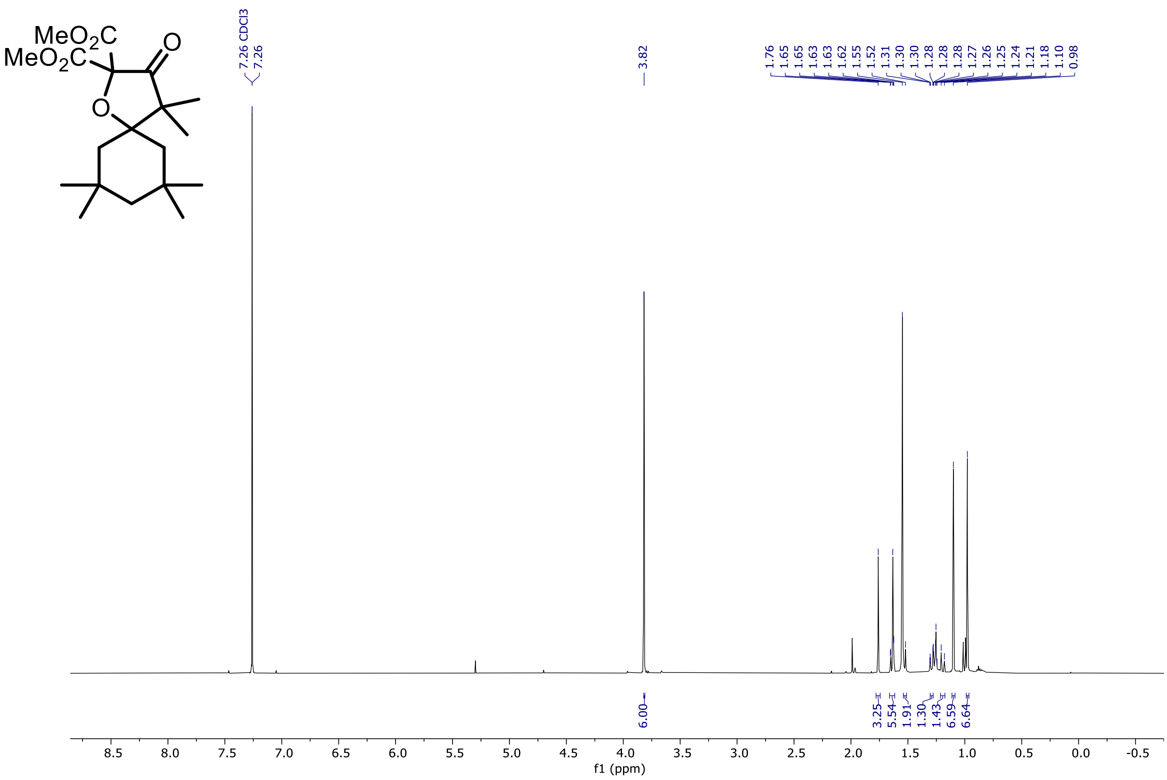

Compound 7i ^{13}C NMR (101 MHz, $CDCl_3$)

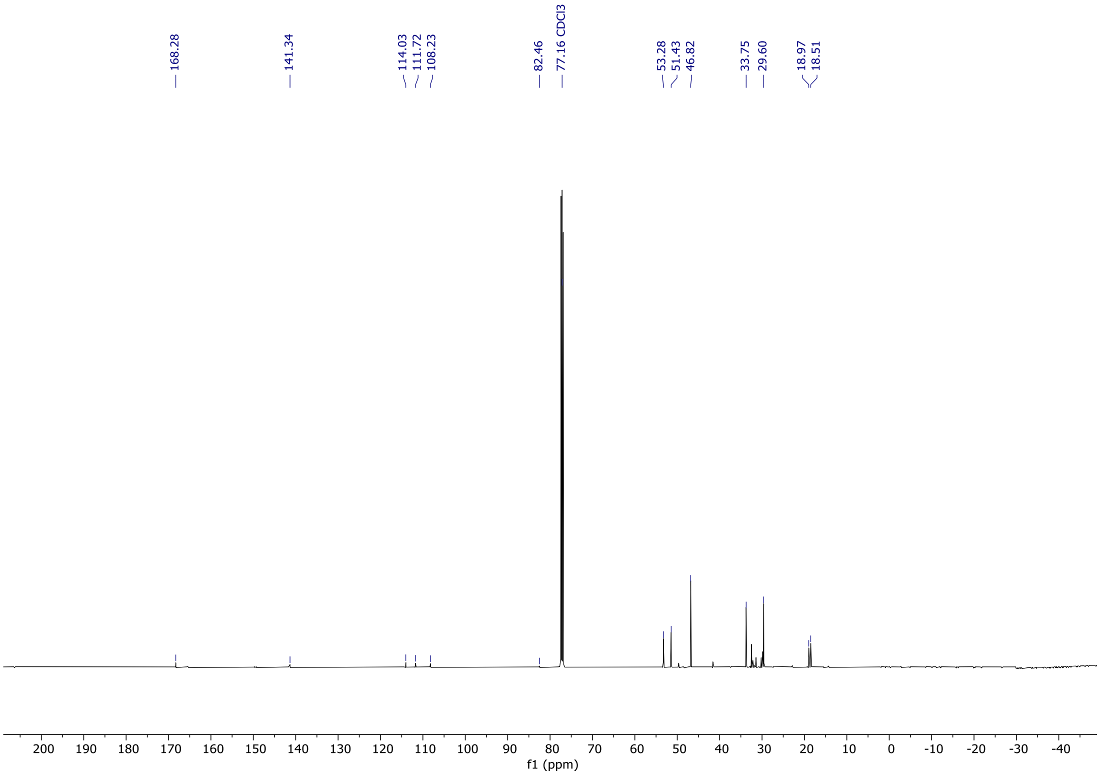

Compound 9a ^{1}H NMR (400 MHz, $CDCl_3$)

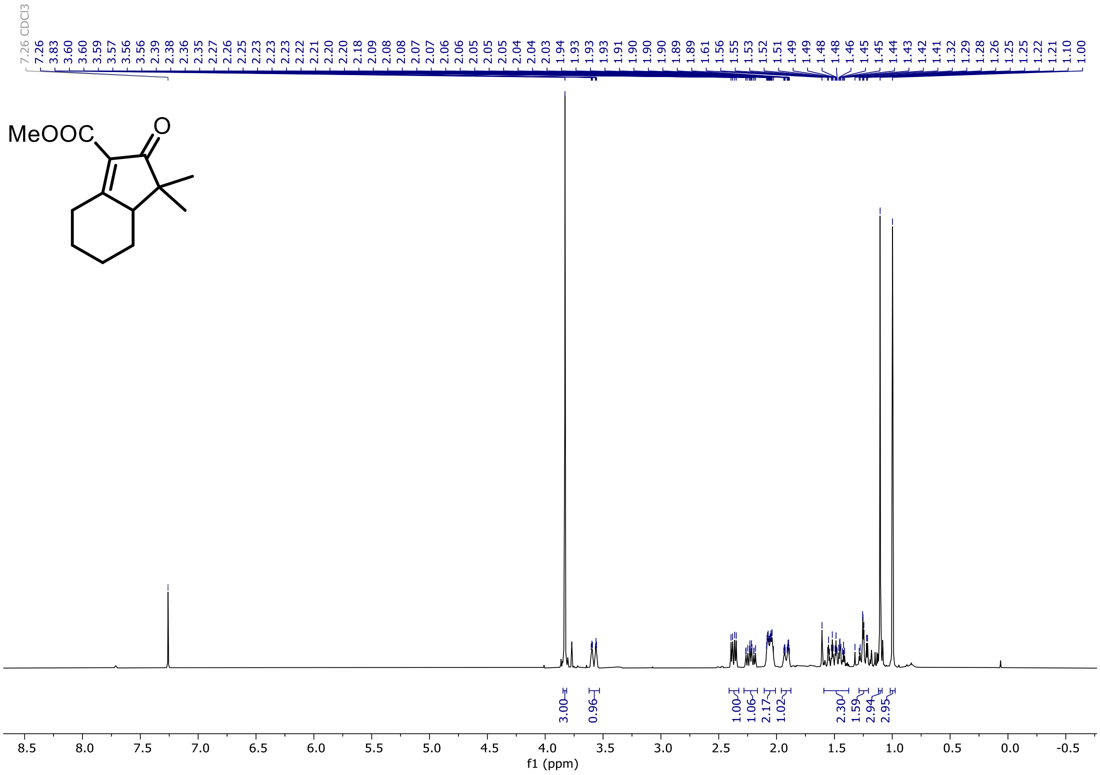

Compound 9a ^{13}C NMR (101 MHz, $CDCl_3$)

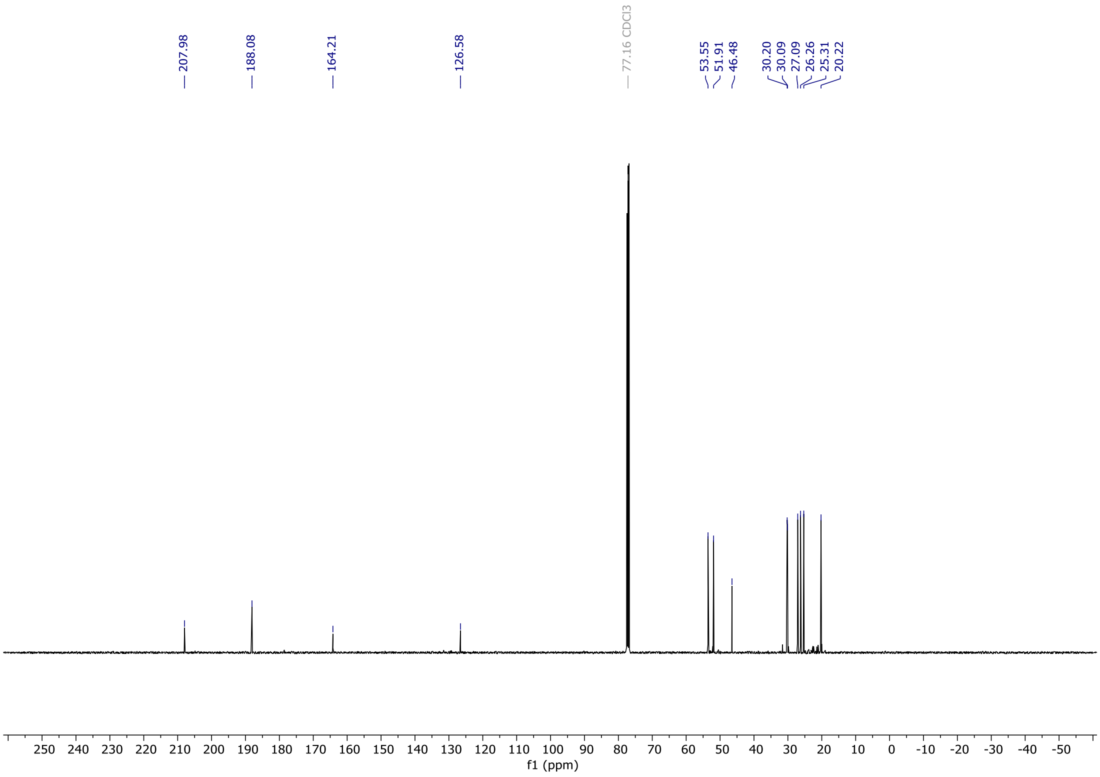

7. Photophysical and Electrochemical data

Absorption and emission spectra of **AO** and **AOH⁺**

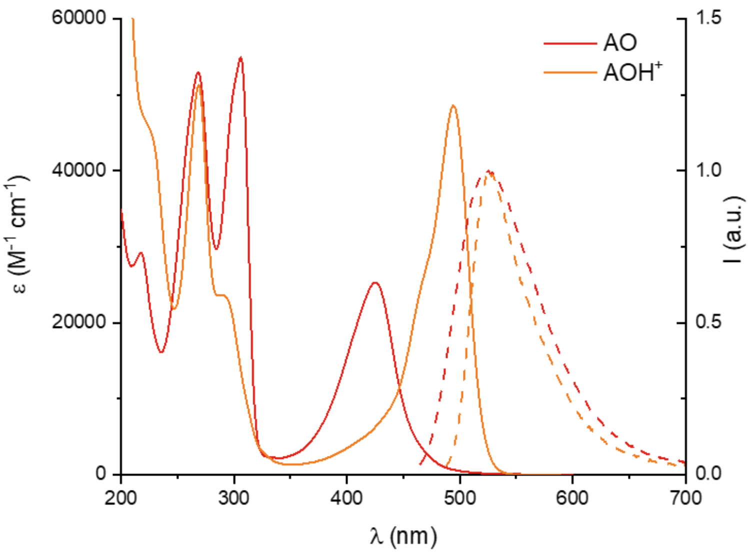

Figure S1. *Absorption (full lines) and normalized emission (dashed lines) spectra of **AO** (red) and protonated **AOH⁺** (orange) in air-equilibrated acetonitrile at RT with concentrations around 1 × 10^{-5} M.*

Absorption and emission spectra of **HE**

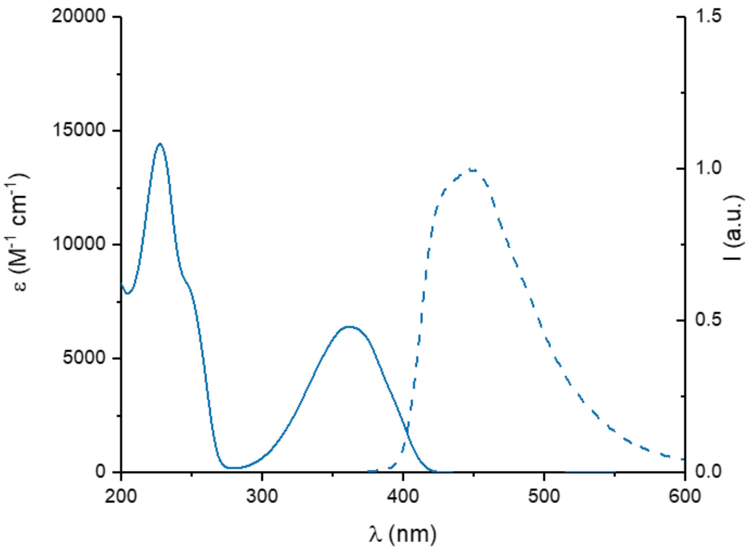

Figure S2. *Absorption (full lines) and normalized emission (dashed lines) spectra of **HE** in air-equilibrated acetonitrile at RT with concentrations around 1 × 10^{-4} M.*

Absorption spectrum of substrate **4a**

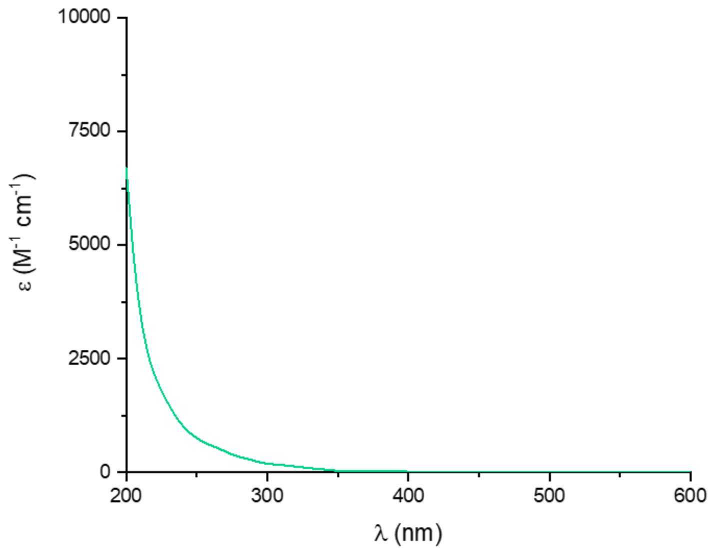

Figure S3. *Absorption spectrum of* ***4a*** *in air-equilibrated acetonitrile at RT with concentrations around 2×10^{-4} M.*

Photophysical data

All following data are in acetonitrile, unless otherwise stated.

Compound[a]	λ_{max} (nm)	ε (M^{-1} cm^{-1})	λ_{em} (nm)	ϕ_f (%)[b]	τ (ns)[e]	k_r (10^6 s^{-1})[f]	k_{nr} (10^6 s^{-1})[g]	E_{00} (eV)
**AO**	425	25300	525	n.d.	n.d.	n.d.	n.d.	2.62
**AOH^+**	494	48570	525	37[c]	2.5	148	252	2.44
**HE**	362	6400	447	1.7[d]	0.32[d]	53	3072	3.06

Table S2. *[a] concentrations 1×10^{-4} to 5×10^{-6} M; [b] ϕ_f estimated error = ±10%; [c] cumarine 153 (ϕ = 53 % in EtOH); [d] data in DMSO [9]; [e] λ_{exc} = 400 nm; [f] k_r = ϕ/τ;* [g] *k_{nr} = (1-ϕ)/τ.*

Electrochemical data

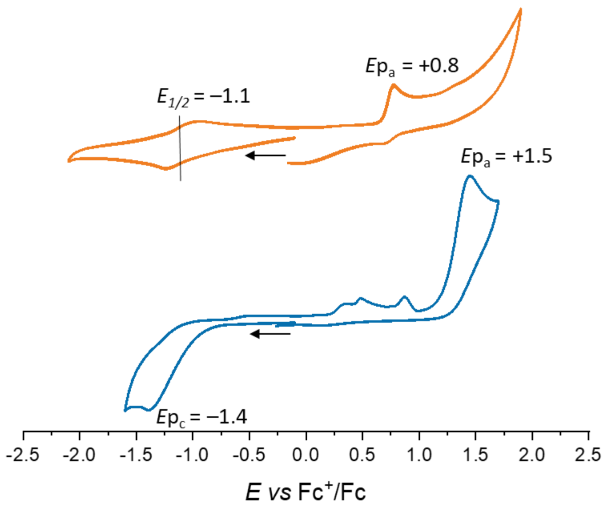

Figure S4. *Cyclic voltammetry curves of* ***AOH⁺*** *(orange) and* ***4a*** *(blue) recorded at Pt electrode (Ø = 3 mm, v = 0.1 V/s) with concentration 5 × 10^{-4} M in dry acetonitrile under inert atmosphere using $[nBu_4N][PF_6]$ 10^{-1} M as supporting electrolyte. The arrows indicate the direction of the scan (negative potential first).*

Extra peaks are visible in the CV curves of **AOH^+** (around 0.0 V) and **4a** (between +0.25 and +1.0 V) due to degradation products formed after the irreversible electrochemical processes.

	Reduction			Oxidation		
Compound	Ep_c	Ep_a	$E_{1/2}$	Ep_a	Ep_c	$E_{1/2}$
**AO**			−2.0[a]			
**AOH^+**	−0.85	−0.60	−0.72	+1.2	-	-
**HE**				+0.93[b]	-	-
**4a**	−1.0	-	-	+1.9	-	-

Table S3. *Half-wave ($E_{1/2}$) and anodic or cathodic peak potentials (Ep_a or Ep_c) for irreversible processes (V vs SCE) in acetonitrile. Conversion of V vs Fc^+/Fc to V vs SCE was done using $E_{1/2}$ (Fc^+/Fc) = +0.4 V vs SCE.* [3] *[a]* [10]*; [b]* [11] *.*

Determination of excited states redox potentials and ΔG_0 of photo-induced SET

For a photo-induced single electron transfer (SET) process, where A is the photocatalyst that reduces the quencher B: $\Delta G_0 = nF\,[E\,(A^+/A^*) - E\,(B/B^-)]$, with A* indicating A at its excited state. The redox potentials of the excited state of A are $E^{red}\,(A^*/A^-) \sim E^{red}\,(A/A^-) + E_{00}(A^*,A)$ and $E^{ox}\,(A^+/A^*) \sim E^{ox}\,(A^+/A) - E_{00}(A^*,A)$.[12]

Considering **AO** as photocatalyst, upon excitation, **AO*** should do a SET to oxidize the **HE** and start the reaction. As E^{red} (**$AO^*/AO^{-\bullet}$**) = +0.62 V and E^{ox} (**$HE^{+\bullet}/HE$**) = +0.93 V, ΔG_0 ≈ +0.93 –

0.62 > 0 making the process not spontaneous. It's this observation that prompted us to consider whether the active photocatalyst was the protonated form of the Acridine Orange (**AOH**$^{+}$).

The potentials of the excited state of **AOH**$^{+}$ are: E^{red} (**AOH**$^{+*}$/**AOH**$^{\bullet}$) = +1.72 V and E^{ox} (**AOH**$^{++\bullet}$/ **AOH**$^{+*}$) = –1.24 V. Which means that **AOH**$^{+*}$ should be able to oxidize the **HE** with $\Delta G_0 \approx +0.93 - 1.72 < 0$. Additionally, **AOH**$^{+*}$ could also reduce directly the enol ether **4a**: $\Delta G_0 \approx -1.24 - (-1.0) < 0$. Due to these considerations, investigation of whether the protonated form of **AO** is present in the reaction mixture were performed (see proposed mechanism).

On another note, when a shorter wavelength of irradiation is used (365 nm) to excite the **HE** directly, **HE***can act as photo-reductant toward **4a**: E^{ox} (**HE**$^{+\bullet}$/**HE***) = –2.13 V, giving $\Delta G_0 \approx -2.13 - (-1.0) < 0$.

<u>Spectroscopic investigation of the reaction mixture</u>

The photochemical reaction was set up with **4a** as substrate, following the general procedure C (*vide supra*). Before irradiating, 5 μL of the reaction mixture were taken and diluted in 2.5 mL of acetonitrile. The absorption spectrum was recorded. After completion of the reaction, another 5 μL of reaction mixture were taken, diluted in 2.5 mL of acetonitrile and the absorption features rechecked.

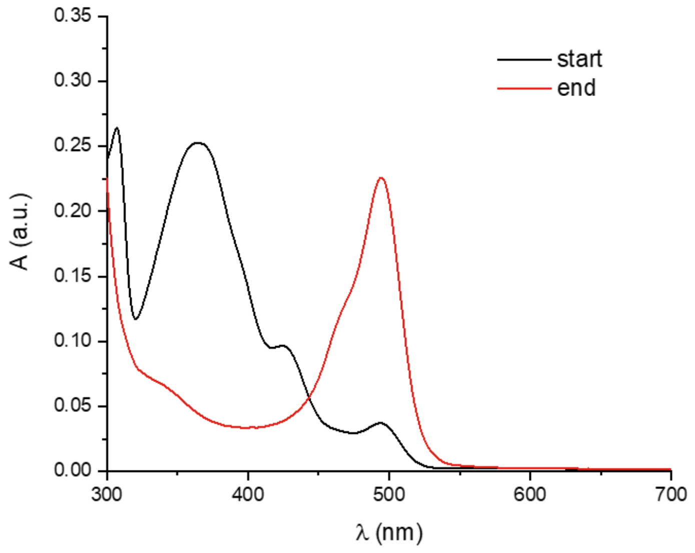

Figure S5. *Absorption spectra of diluted aliquots of the reaction mixture (**AO** as starting catalyst) taken at the beginning (black) and end (red) of the reaction.*

As visible in Figure S5, in the absorption spectrum before irradiation, a peak at 494 nm already appears, attesting the presence of **AOH⁺** in solution. By the end of the reaction, this absorption feature is predominant, showing how most of **AO** is converted to **AOH⁺** during the reaction.

As a control experiment, the same procedure was followed but using **AOH⁺** instead of **AO** in the general procedure C.

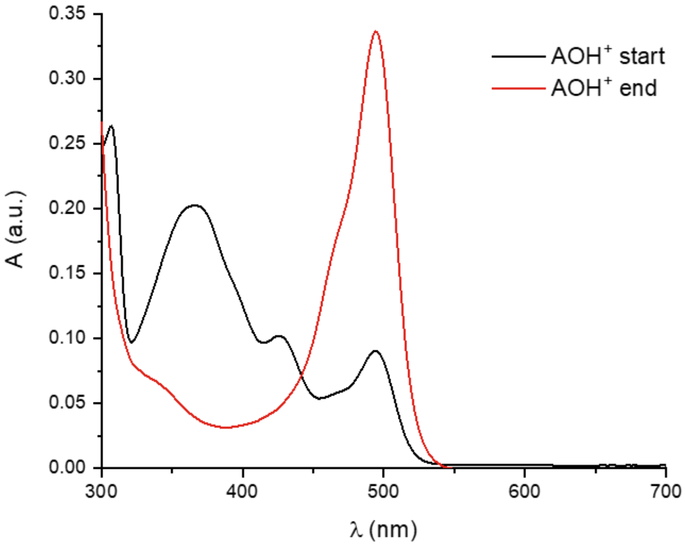

Figure S6 *Absorption spectra of diluted aliquots of the reaction mixture (**AOH⁺** as starting catalysis) taken at the beginning (black) and end (red) of the reaction.*

The spectral features shown in Figure S6, for both the starting and ending spectra of the reaction mixture, are similar to those reported in Figure S5. This result shows how, regardless of whether **AO** or **AOH⁺** are introduced in the reaction mixture, an equilibrium takes place making **AOH⁺** available in solution from the beginning. **AOH⁺** can then act as the active photocatalyst and, by the end of the reaction, most **AO** is protonated. Furthermore, it was verified that the two reactions bring to the same yield of product **5a**.

<u>Source of **AO** protonation</u>

As it was hypothesized that **HE** could be the source of protons for **AO** → **AOH⁺**, the absorption spectra of a solution of **AO** was recorded upon addition of 1 or 2 equivalents of **HE**.

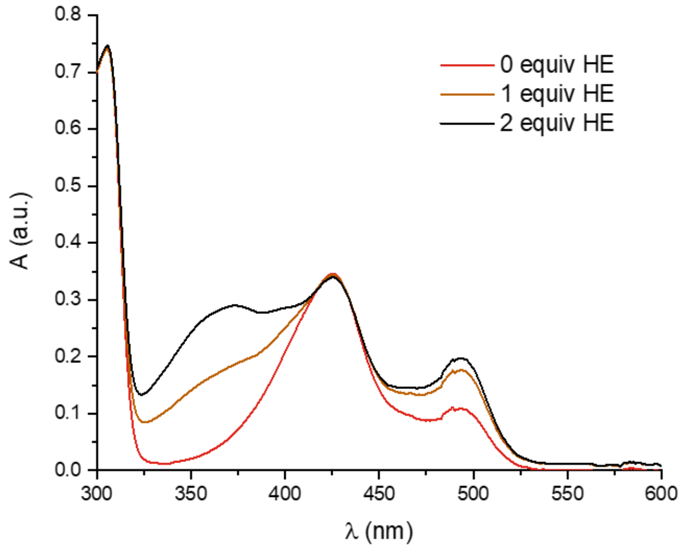

Figure S7. *Absorption spectra of **AO** (red line), **AO** + 1 equiv **HE** (brown line) and **AO** + 2 equiv **HE** (black line) in air-equilibrated acetonitrile at RT with **AO** concentrations around 1 × 10^{-5} M.*

Surprisingly, looking at the spectrum of **AO** (Figure S7, red line), partial protonation of **AO** is already present before addition of **HE**. However, adding 1 and then 2 equivalents of **HE**, the peak at 494 nm increases in intensity showing an increase in concentration of **AOH^+**. In the reaction conditions (general procedure C), **AO** (5 μmol) is 100 time less than **HE** (0.5 mmol). This could be the source of protonation of **AO** to **AOH^+** during the reaction evolution.

8. Quenching experiments

Quenching experiments were performed measuring the emission profile of the photo-excited species upon increasing concentration of the quencher ([Q]). The emission intensity was then plotted at single wavelength *vs* [Q] and fitted using the Stern-Volmer equation ($I^0/I = 1 + \tau^0 k_{SET}[Q]$). Measurements were performed making sure that: (i) the same experimental settings of the spectrofluorimeter were used during the whole quenching experiment, (ii) excitation and emission wavelengths were not influenced by the increasing concentration of the quencher (*i.e.* same overall A at the λ_{exc} and no reabsorption at λ_{em}), (iii) concentration of the photo-excited species was such to have A ≈ 0.3 -0.5 at λ_{max}.[12] All quenching experiments were carried out in analytical grade extra dry acetonitrile (AcroSeal®) under inert atmosphere, using UHV stopcock quartz cuvettes.

The efficiency of each quenching process (η_Q) was calculated using:

$$\eta_{Q1} = \frac{k_{Q1}[Q1]}{k_r + k_{nr} + k_{Q1}[Q1] + k_{Q2}[Q2]}$$

where Q1 and Q2 are two possible quenchers and k_Q the relative quenching constant.

Substrate **4a** is sometime indicated as “sub”.

Quenching of **AOH⁺** by **HE**

In the reaction mixture, 2.5 equivalents of **HE** are used for a theoretical [**HE**] = 0.25 M. However, most of the **HE** is not in solution. The [**HE**]$_{sat}$ was determined by making a saturated solution, taking a known volume of the supernatant liquid and recording its absorption spectrum after appropriate dilution. [**HE**]$_{sat}$ = 0.02 M. In order to observe a meaningful quenching of the **AOH⁺** emission without affecting the overall solution volume in the cuvette, **HE** was added as a solid to the **AOH⁺** solution (2.5 mL).

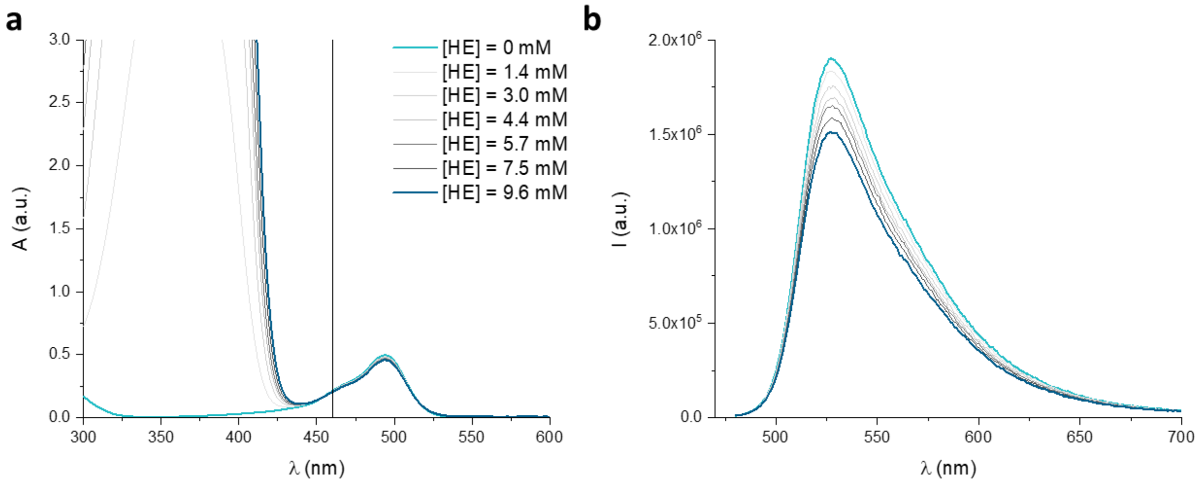

Figure S8. *a) absorption and b) emission spectra of **AOH⁺** (10^{-5} M, light blue line) upon increasing amount of **HE** (up to 9.6 mM, blue line). In a) the vertical line indicates λ_{exc} = 460 nm.*

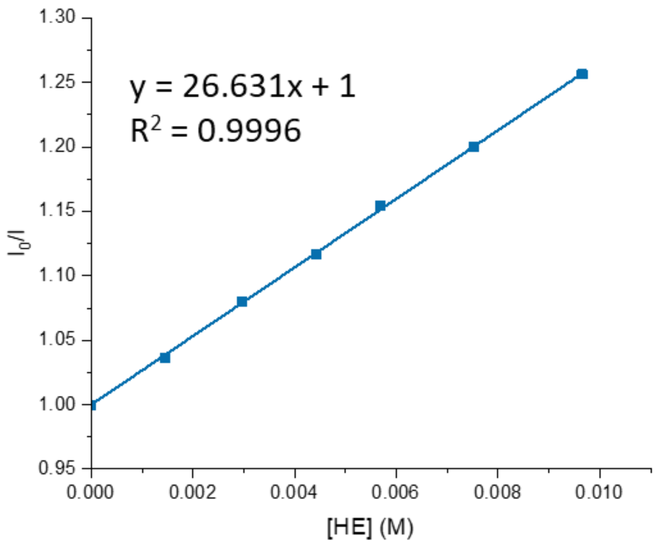

Figure S9*. Stern-Volmer diagram relative to the fluorescence quenching of Figure S8. λ_{em} = 529 nm.*

$\tau^0 k_{SET}$ = 26.63 M^{-1} → $\mathbf{k_{SET} = 1.1 \times 10^{10}\ M^{-1}s^{-1}}$ and with $[\mathbf{HE}]_{sat}$ = 0.02 M → $\mathbf{\eta_{HE} = 28\%}$.

<u>Quenching of **AOH⁺** by **4a**</u>

The concentration of **4a** in the reaction mixture is 0.1 M.

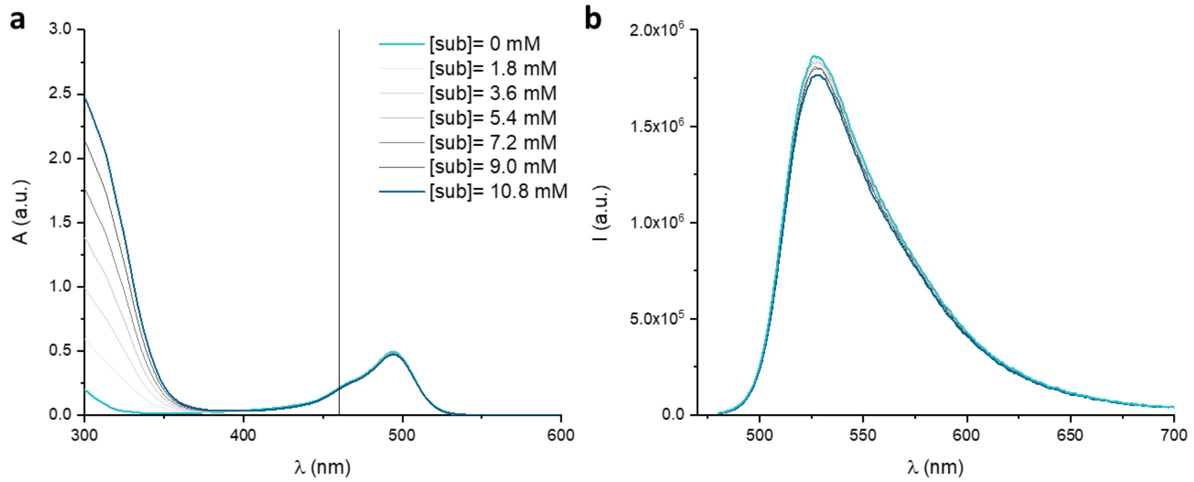

Figure S10*. a) absorption and b) emission spectra of **AOH^+** (10^{-5} M, light blue line) upon increasing amount of **4a** (up to 10.8 mM, blue line). In a) the vertical line indicates λ_{exc} = 460 nm.*

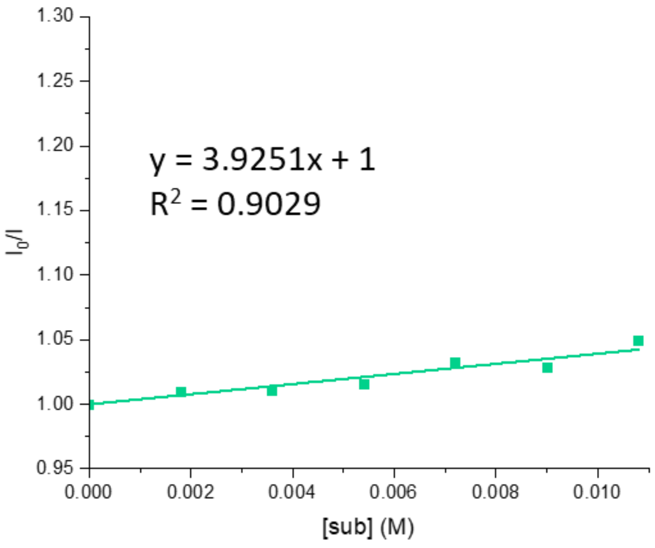

Figure S11. *Stern-Volmer diagram relative to the fluorescence quenching of Figure S10. λ_{em} = 529 nm.*

$\tau^0 k_{SET}$ = 3.92 M^{-1} → **k_{SET} = 1.6 × 10^9 $M^{-1}s^{-1}$** and with [**4a**] = 0.1 M → **η_{4a} = 20%.**

<u>Efficiencies of **AOH^+** photo-induced SET processes in time</u>

Since the **HE** in the reaction mixture is saturated, its concentration will remain constant during the reaction evolution while the substrate is consumed. Taking this into account, it is possible to plot both **η_{HE}** and **η_{4a}** as a function of [**4a**].

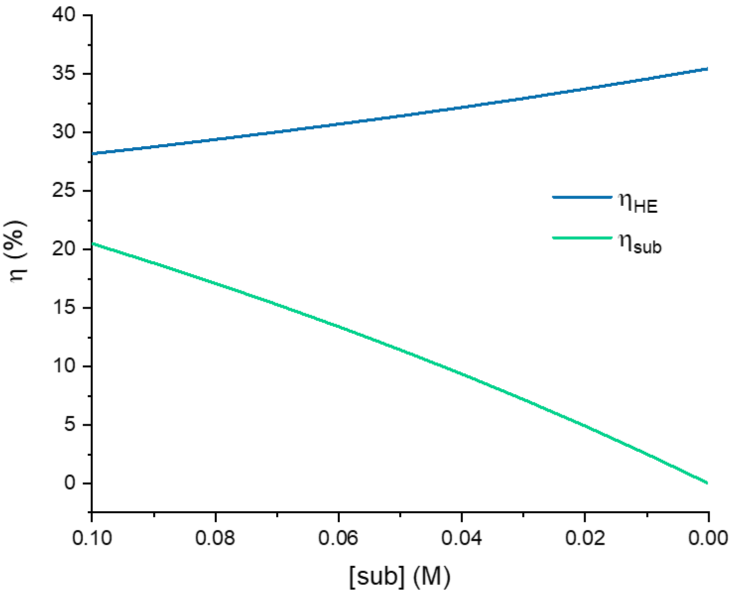

Figure S12. *η_{HE} and η_{4a} as a function of [**4a**].*

The quenching of the **AOH**$^{+*}$ by the substrate becomes less efficient as its concentration decreases during the reaction. At the same time, the quenching efficiency with **HE** increases.

<u>Quenching of **HE** by **4a**</u>

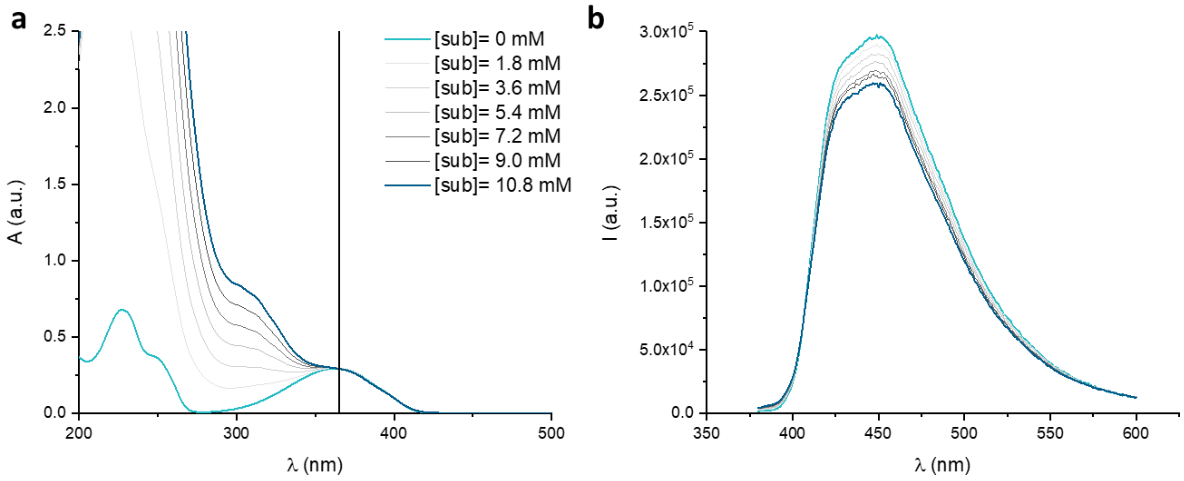

Figure S13. *a) absorption and b) emission spectra of* ***HE*** *(5 × 10*$^{-5}$ *M, light blue line) upon increasing amount of* ***4a*** *(up to 10.8 mM, blue line). In a) the vertical line indicates* λ_{exc} *= 365 nm.*

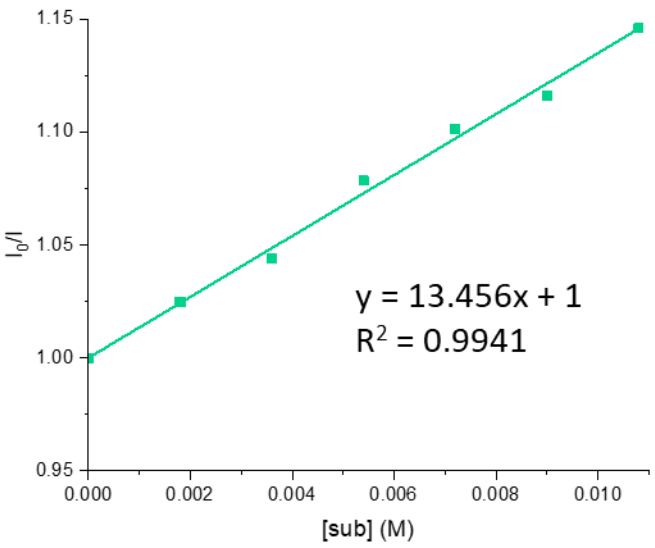

Figure S14. *Stern-Volmer diagram relative to the fluorescence quenching of Figure S13.* λ_{em} *= 455 nm.*

$\tau^0 k_{SET}$ = 13.46 M^{-1} → $\mathbf{k_{SET} = 4.2 \times 10^{10}\ M^{-1}s^{-1}}$ and with [**4a**] = 0.1 M → $\mathbf{\eta_{4a} = 57\%}$.

9. Mechanistic studies

9.1. Path *ii*

As mentioned, acridine orange **AO** reacts with **HE** to generate **AOH⁺**, which is then excited at 455 nm, followed by oxidation by enol ether **4a** to form the **AOH²⁺•** radical. The resulting radical intermediate **A** is formed directly with this path *ii*. To finish the catalytic cycle, SET between **HE** and **AOH²⁺•** occurs to get **AOH⁺** back to its ground state and form **HE⁺•** that will be useful for the final HAT of **C** to **6a.**

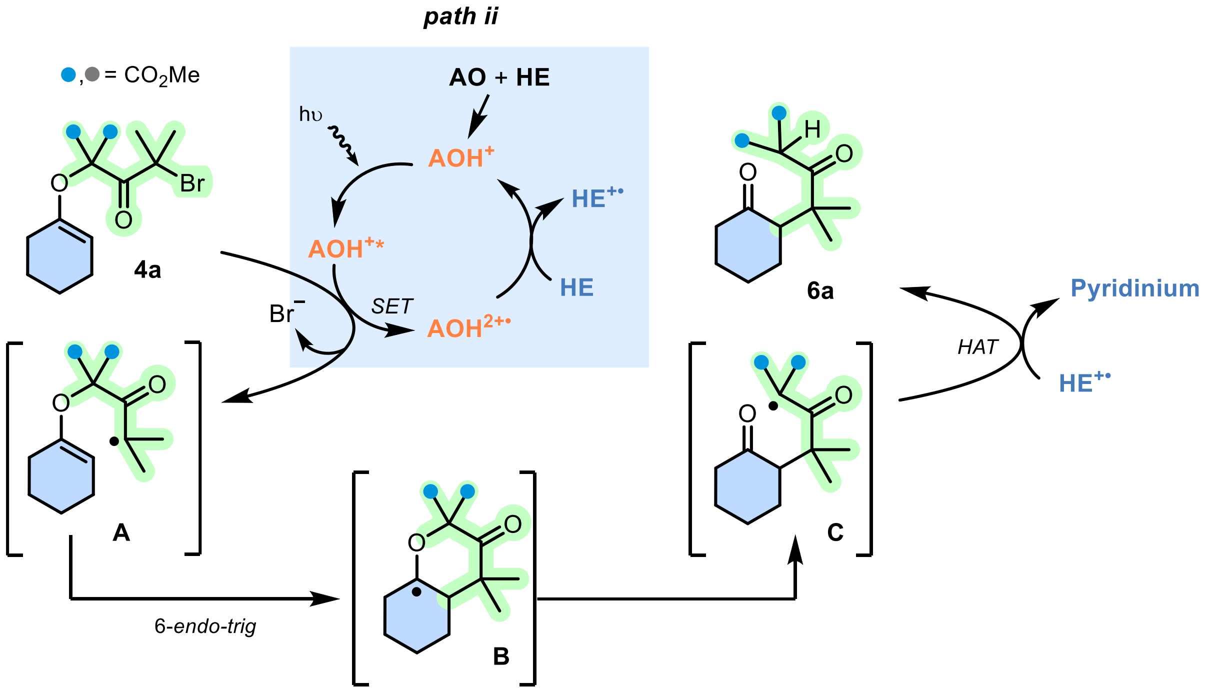

Scheme S2. *6-endo-trig* cyclization followed by intramolecular aldolization.

9.2 Fused vs spiro cycle formation – Comparison **A**, **A1**, **A2** and **A3**

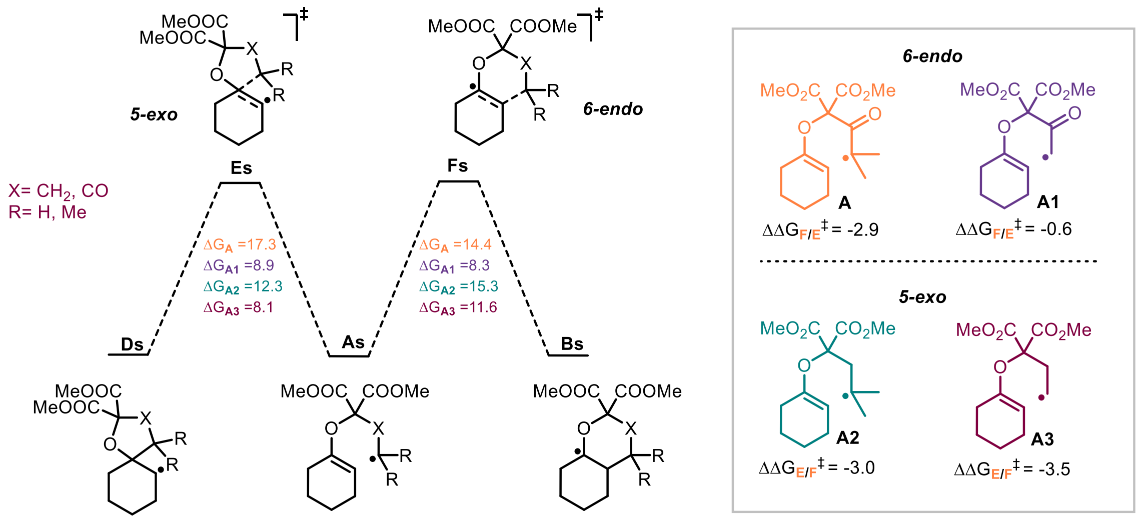

Figure S15. *DFT calculations of* 5-*exo and* 6-*endo cyclizations.* Gibbs energies are given in kcal/mol.

9.3 Temptative concerted formation of **5a**

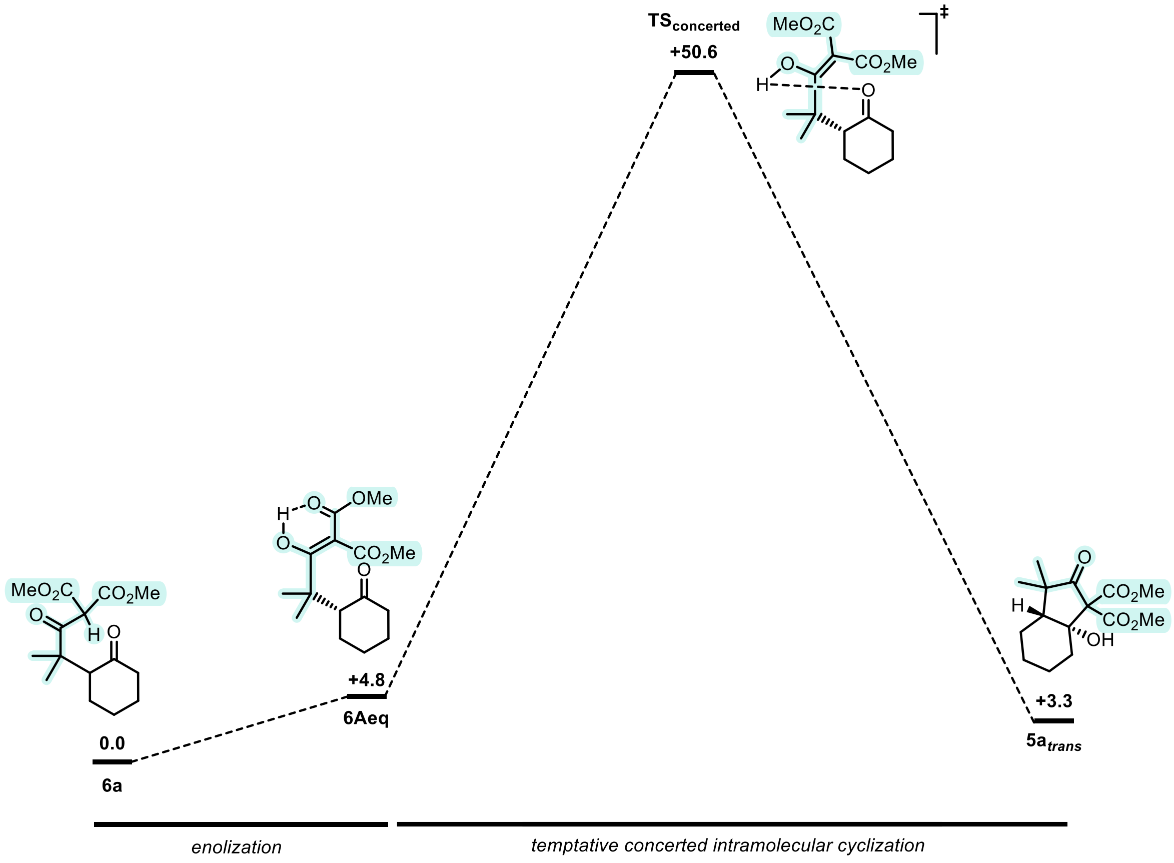

Scheme S3. *DFT calculation of the tentative concerted pathway.* Gibbs energies are given in kcal/mol.

9.4 Computational benchmarking

Single point calculations of all species were computed using wB97XD as method and def2-TZVPD as basis set for all atoms, using the SMD model to represent the dichloromethane solution (ε = 8.93). The energies are detailed in Table S4 and show the same trend as the one detailed in the main manuscript. The reported energies are free energies in solution calculated at 298 K and 1 atm in kcal/mol.

Compound	Energy (kcal/mol)
6a	0.0
6Aeq	+4.1
6Aax	+8.3
TS1trans	+20.1
TS1cis	+36.9
6Beq	+20.3
6Bax	+23.0
TS2trans	+31.4
TS2cis	+42.2
5a	-0.2
5acis	+5.1

Table S4. *Computational benchmark.*

9.5 Diastereoselective formation of **5j** and **5k**

To rationalize the preferential axial disposition of the distal CF_3 and CH_3 substituents in products **5j** and **5k**, the main elements of the proposed mechanism were analyzed from tridimensional and conformational points of view. Substituted radical **A'** presents two conformations, the most stable presenting the distal substituent in equatorial position (**A'eq**). The radical addition on the β-carbon of **A'** would need to be in axial to achieve a chair conformation of **B'**, otherwise an equatorial attack would lead to a less stable a twisted boat conformation. If such attack is considered with **A'ax**, the resulting bicycle **B'cis** should be more unstable due to 1,3-diaxial interaction than analogue **B'*trans*** formed from **A'eq**. In both cases, after ring opening to generate **C'**, the largest substituent, the chain, will assume an equatorial position. Malonyl radical **C'** will then abstract an hydrogen (HAT), and then finally cyclize *via* an equatorial attack to form the *trans*-fused bicycle under aldolization conditions (**5j** or **5k**). Taking all this into consideration, the diasteroselectivity of the reaction is primarily driven by the first 6-*endo-trig* cyclization as **A'eq** reacts faster and it then leads to the fused bicycle having the distal substituent in axial position in respect to the 6-membered ring (**5j-*trans*** and **5k-*trans***).

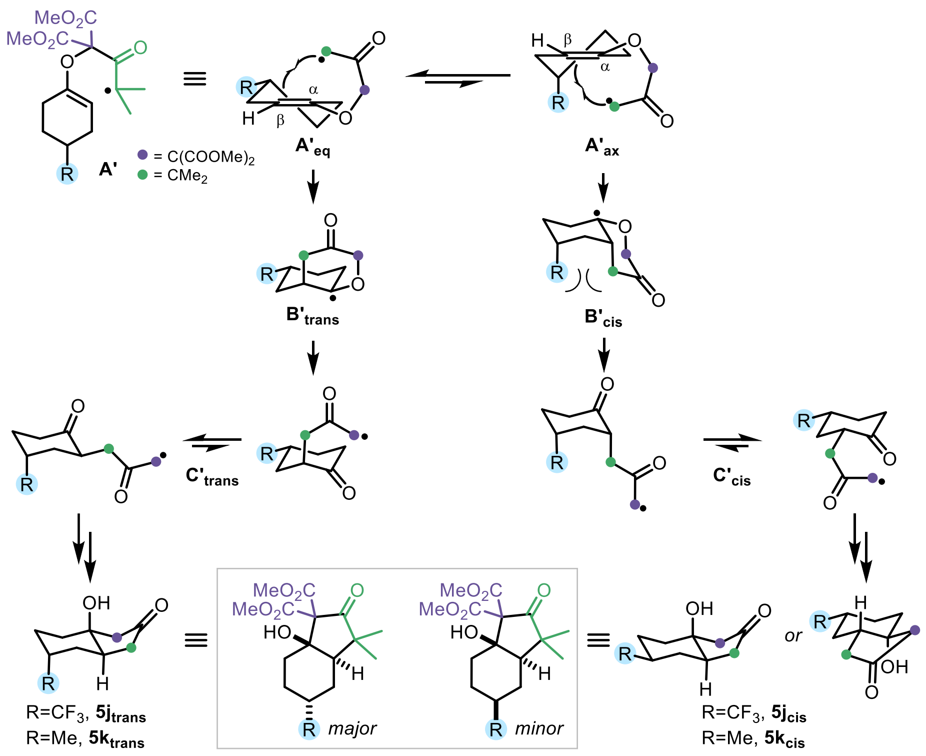

Scheme S4. *Conformational analysis of the proposed mechanism*.

10. Crystallographic data

Suitable crystals were selected and X-ray intensity data were collected on a. Rigaku XtaLAB Synergy, Dualflex, HyPix-Arc 150° diffractometer using Cu Kα radiation (λ = 1.54184 Å).

Using Olex2[13], the structures were solved with the SHELXT[14] structure solution program using dual space methods and refined with the SHELXL[15] refinement package using Least Squares minimization. Summaries of crystal data and structure refinement are given below.

Compound 5a

CCDC Number	2058766	
Empirical formula	C15 H22 O6	
Formula weight	298.32	
Temperature	119.99(17) K	
Wavelength	1.54184 Å	
Crystal system	Monoclinic	
Space group	P 1 21/c 1	
Unit cell dimensions	a = 15.9282(4) Å	α= 90°
	b = 11.6609(3) Å	β= 91.550(2)°
	c = 16.3365(3) Å	γ = 90°
Volume	3033.20(12) $Å^3$	
Z	8	
Density (calculated)	1.307 Mg/m^3	
Absorption coefficient	0.841 mm^{-1}	
F(000)	1280	
Crystal size	0.108 x 0.048 x 0.013 mm^3	
Theta range for data collection	2.775 to 70.230°.	
Index ranges	-19<=h<=18, -13<=k<=14, -19<=l<=19	
Reflections collected	24486	
Independent reflections	5677 [R(int) = 0.0589]	
Completeness to theta = 67.684°	99.9 %	
Absorption correction	Gaussian	
Max. and min. transmission	1.000 and 0.934	
Refinement method	Full-matrix least-squares on F^2	
Data / restraints / parameters	5677 / 0 / 393	
Goodness-of-fit on F^2	1.082	

Final R indices [I>2sigma(I)]	R1 = 0.0492, wR2 = 0.1350
R indices (all data)	R1 = 0.0600, wR2 = 0.1422
Largest diff. peak and hole	0.299 and -0.259 e.Å^{-3}

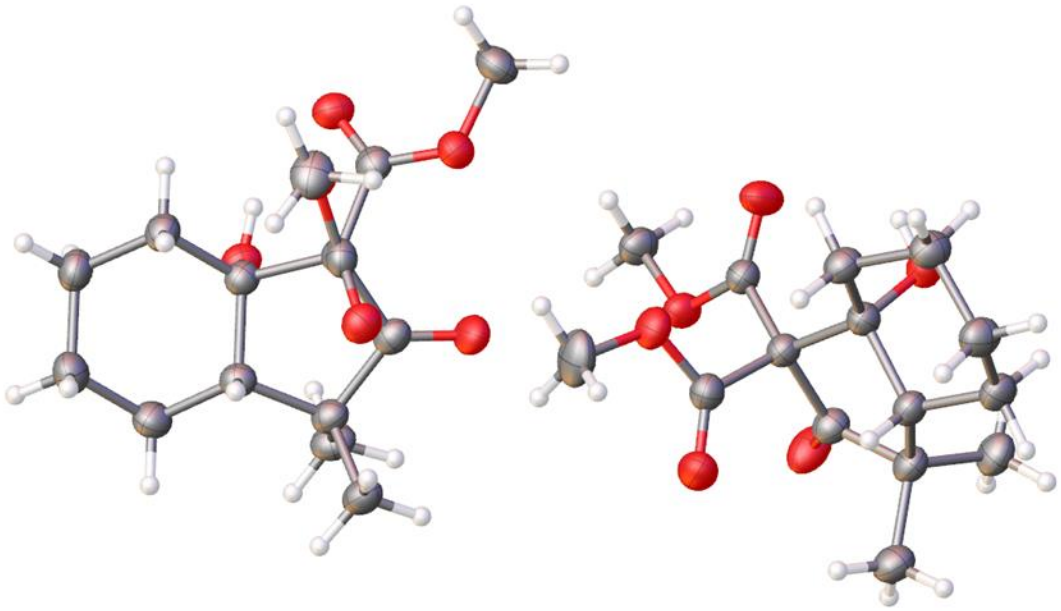

Figure S16. Compound **5a**. View of the asymmetric unit (displacement ellipsoid at 50 percent probability level)

Compound 5c

CCDC Number	2481640	
Empirical formula	C16 H24 O6	
Formula weight	312.35	
Temperature	120.00(10) K	
Wavelength	1.54184 Å	
Crystal system	Orthorhombic	
Space group	Pbca	
Unit cell dimensions	a = 6.91547(5) Å	α= 90°
	b = 14.62954(12) Å	β= 90°
	c = 31.3354(2) Å	γ = 90°
Volume	3170.21(4) $Å^3$	
Z	8	
Density (calculated)	1.309 Mg/m^3	
Absorption coefficient	0.827 mm^{-1}	
F(000)	1344	
Crystal size	0.265 x 0.105 x 0.044 mm^3	
Theta range for data collection	2.820 to 74.453°.	
Index ranges	-8<=h<=8, -16<=k<=18, -38<=l<=39	
Reflections collected	71531	
Independent reflections	3229 [R(int) = 0.0327]	
Completeness to theta = 67.684°	100.0 %	
Absorption correction	Gaussian	
Max. and min. transmission	1.000 and 0.648	
Refinement method	Full-matrix least-squares on F^2	
Data / restraints / parameters	3229 / 0 / 206	
Goodness-of-fit on F^2	1.037	
Final R indices [I>2sigma(I)]	R1 = 0.0363, wR2 = 0.0908	
R indices (all data)	R1 = 0.0382, wR2 = 0.0921	
Largest diff. peak and hole	0.270 and -0.229 e.$Å^{-3}$	

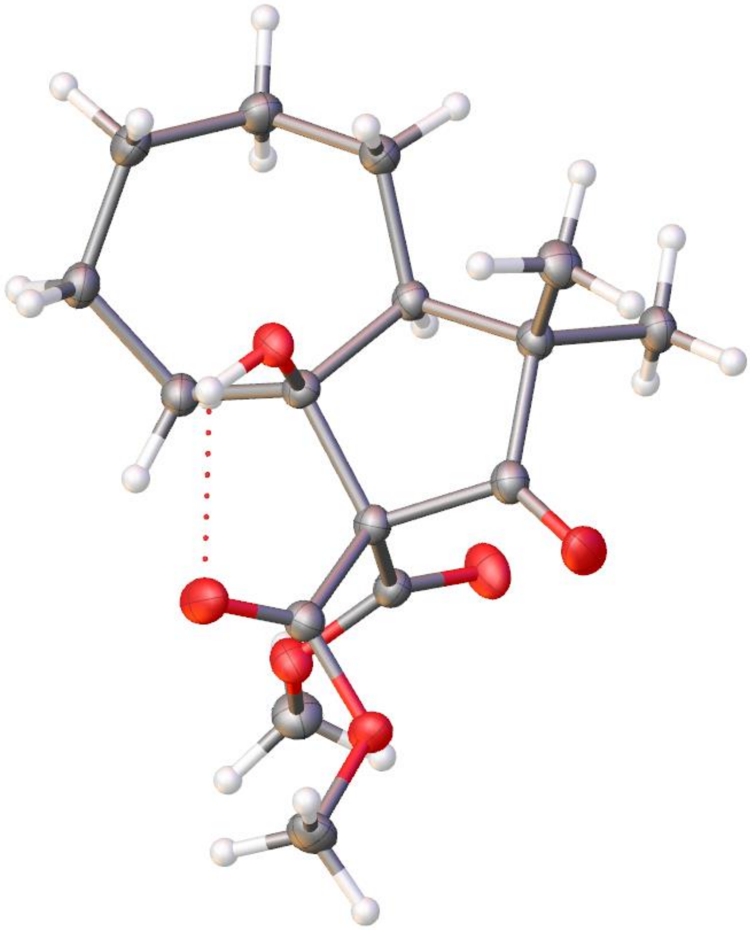

Figure S17. Compound **5c**. View of the asymmetric unit (displacement ellipsoid at 50 percent probability level)

Compound 5k

CCDC Number	2481639	
Empirical formula	C16 H24 O6	
Formula weight	312.35	
Temperature	120.00(10) K	
Wavelength	1.54184 Å	
Crystal system	Monoclinic	
Space group	P 1 21/c 1	
Unit cell dimensions	a = 16.0904(3) Å	α= 90°
	b = 8.41198(10) Å	β= 101.8598(16)°
	c = 24.6068(4) Å	γ = 90°
Volume	3259.49(9) $Å^3$	
Z	8	
Density (calculated)	1.273 Mg/m^3	
Absorption coefficient	0.805 mm^{-1}	
F(000)	1344	
Crystal size	0.446 x 0.143 x 0.016 mm^3	
Theta range for data collection	2.806 to 74.574°.	
Index ranges	-19<=h<=19, -10<=k<=10, -30<=l<=29	
Reflections collected	10387	
Independent reflections	10387	
Completeness to theta = 67.684°	99.9 %	
Absorption correction	Semi-empirical from equivalents	
Max. and min. transmission	1.00000 and 0.70020	
Refinement method	Full-matrix least-squares on F^2	
Data / restraints / parameters	10387 / 0 / 414	
Goodness-of-fit on F^2	1.103	
Final R indices [I>2sigma(I)]	R1 = 0.0438, wR2 = 0.1133	
R indices (all data)	R1 = 0.0474, wR2 = 0.1154	
Largest diff. peak and hole	0.256 and -0.252 e.$Å^{-3}$	

Comments on the structure:

The crystal was twinned. Two components were integrated and an hklf5 file was used to perform the refinement in SHELXL.

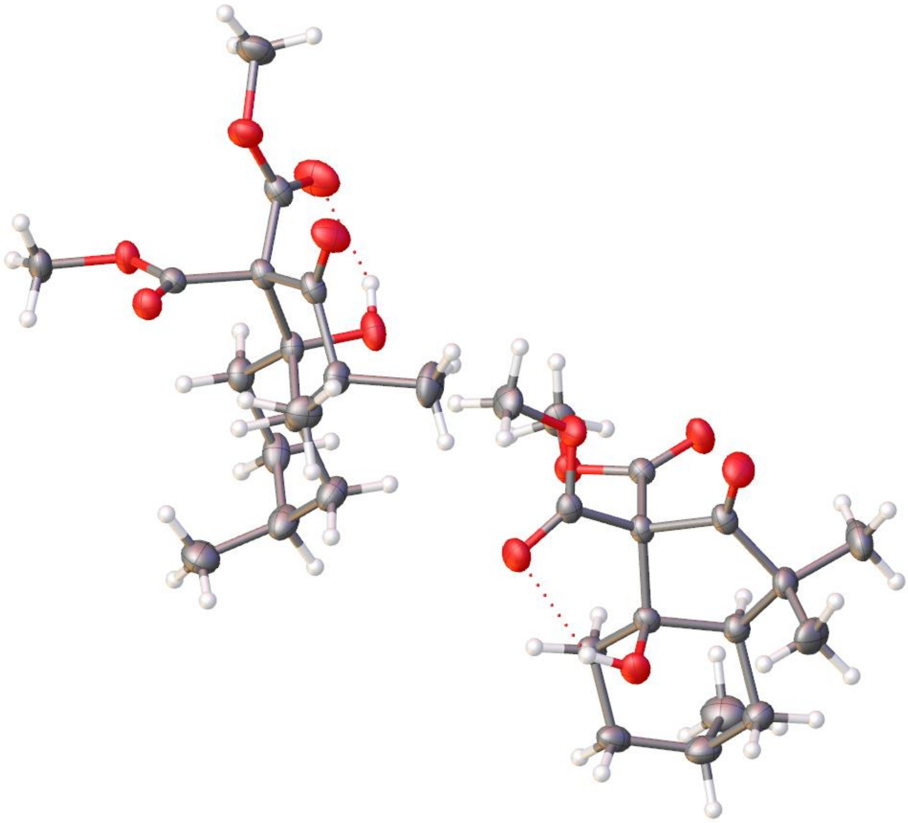

Figure S18. Compound **5k**. View of the asymmetric unit (displacement ellipsoid at 50 percent probability level)

Compound 5j

CCDC Number	2481641	
Empirical formula	C16 H21 F3 O6	
Formula weight	366.33	
Temperature	100.00(11) K	
Wavelength	1.54184 Å	
Crystal system	Monoclinic	
Space group	P 1 21/c 1	
Unit cell dimensions	a = 13.3304(3) Å	α= 90°
	b = 8.40900(10) Å	β= 109.264(2)°
	c = 16.2362(3) Å	γ = 90°
Volume	1718.10(6) $Å^3$	
Z	4	
Density (calculated)	1.416 Mg/m^3	
Absorption coefficient	1.111 mm^{-1}	
F(000)	768	
Crystal size	0.372 x 0.251 x 0.035 mm^3	
Theta range for data collection	3.512 to 71.004°.	
Index ranges	-16<=h<=15, -9<=k<=10, -19<=l<=18	
Reflections collected	20828	
Independent reflections	3256 [R(int) = 0.0217]	
Completeness to theta = 67.684°	99.9 %	
Absorption correction	Gaussian	
Max. and min. transmission	1.000 and 0.377	
Refinement method	Full-matrix least-squares on F^2	
Data / restraints / parameters	3256 / 0 / 233	
Goodness-of-fit on F^2	1.045	
Final R indices [I>2sigma(I)]	R1 = 0.0319, wR2 = 0.0836	
R indices (all data)	R1 = 0.0343, wR2 = 0.0851	
Largest diff. peak and hole	0.277 and -0.254 e.$Å^{-3}$	

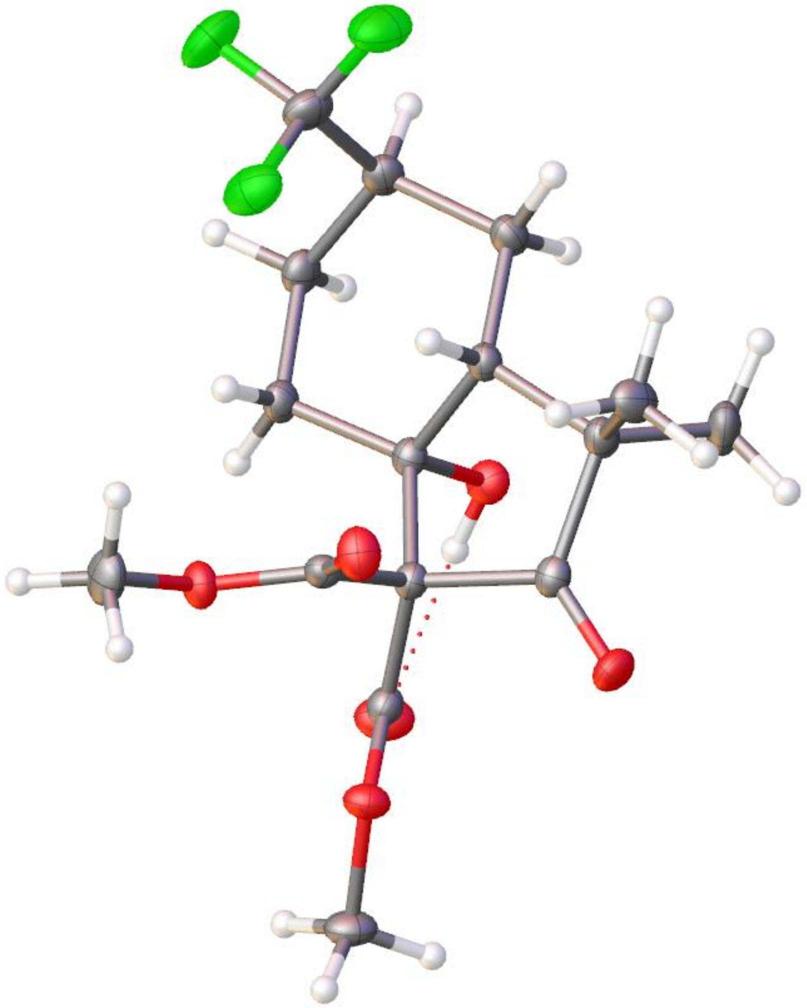

Figure S19. Compound **5j**. View of the asymmetric unit (displacement ellipsoid at 50 percent probability level)

11. References

1 https://www.thorlabs.com/thorproduct.cfm?partnumber=M455L3

2 https://peschl-ultraviolet.com/?lang=en

3 N. G. Connelly and W. E. Geiger, *Chem. Rev.*, 1996, **96**, 877-910.

4 M. J. Frisch, G. W. Trucks, H. B. Schlegel, G. E. Scuseria, M. A. Robb, J. R. Cheeseman, G. Scalmani, V. Barone, G. A. Petersson, H. Nakatsuji, X. Li, M. Caricato, A. V. Marenich, J. Bloino, B. G. Janesko, R. Gomperts, B. Mennucci, H. P. Hratchian, J. V. Ortiz, A. F. Izmaylov, J. L. Sonnenberg, Williams, F. Ding, F. Lipparini, F. Egidi, J. Goings, B. Peng, A. Petrone, T. Henderson, D. Ranasinghe, V. G. Zakrzewski, J. Gao, N. Rega, G. Zheng, W. Liang, M. Hada, M. Ehara, K. Toyota, R. Fukuda, J. Hasegawa, M. Ishida, T. Nakajima, Y. Honda, O. Kitao, H. Nakai, T. Vreven, K. Throssell, J. A. Montgomery Jr., J. E. Peralta, F. Ogliaro, M. J. Bearpark, J. J. Heyd, E. N. Brothers, K. N. Kudin, V. N. Staroverov, T. A. Keith, R. Kobayashi, J. Normand, K. Raghavachari, A. P. Rendell, J. C. Burant, S. S. Iyengar, J. Tomasi, M. Cossi, J. M. Millam, M. Klene, C. Adamo, R. Cammi, J. W. Ochterski, R. L. Martin, K. Morokuma, O. Farkas, J. B. Foresman and D. J. Fox, Wallingford, CT, 2016.

5 (a) A. D. Beck, *J. Chem. Phys*, 1993, **98**, 5648-5646; (b) C. Lee, W. Yang and R. G. Parr, *Phys. Rev. B*, 1988, **37**, 785; (c) P. J. Stephens, F. J. Devlin, C. F. Chabalowski and M. J. Frisch, *J. Phys. Chem.*, 1994, **98**, 11623-11627; (d) S. Grimme, J. Antony, S. Ehrlich and H. Krieg, *J. Chem. Phys.*, 2010, **132**, 154104.

6 (a) M. M. Francl, W. J. Pietro, W. J. Hehre, J. S. Binkley, M. S. Gordon, D. J. DeFrees and J. A. Pople, *J. Chem. Phys.*, 1982, **77**, 3654-3665; (b) P. C. Hariharan and J. A. Pople, *Theor. Chim. Acta*, 1973, **28**, 213-222; (c) W. J. Hehre, R. Ditchfield and J. A. Pople, *J. Chem. Phys.*, 1972, **56**, 2257-2261.

7 A. V. Marenich, C. J. Cramer and D. G. Truhlar, *J. Phys. Chem. B*, 2009, **113**, 6378-6396.

8 J. Viñas-Lóbez, G. Levitre, A. de Aguirre, C. Besnard, A. I. Poblador-Bahamonde and J. Lacour, *ACS Org. & Inorg. Au*, 2021, **1**, 11-17.

9 (a) S. Azizi, G. Ulrich, M. Guglielmino, S. le Calvé, J. P. Hagon, A. Harriman and R. Ziessel, *J. Phys. Chem. A*, 2015, **119**, 39-49; (b) J. Jung, J. Kim, G. Park, Y. You and E. J. Cho, *Adv. Synth. Catal.*, 2016, **358**, 74-80.

10 T. T. Eisenhart and J. L. Dempsey, *J. Am. Chem. Soc.*, 2014, **136**, 12221-12224.

11 F. Calogero, S. Potenti, E. Bassan, A. Fermi, A. Gualandi, J. Monaldi, B. Dereli, B. Maity, L. Cavallo, P. Ceroni and P. G. Cozzi, *Angew. Chem. Int. Ed.*, 2022, **61**, e202114981.

12 V. Balzani, P. Ceroni and A. Juris, *Photochemistry and photophysics : concepts, research, applications*, Wiley-VCH, Weinheim, 2014.

13 O. V. Dolomanov, L. J. Bourhis, R. J. Gildea, J. A. K. Howard and H. Puschmann, *J. Appl. Crystallogr.*, 2009, **42**, 339-341.

14 G. Sheldrick, *Acta Crystallogr., Sect. A*, 2015, **71**, 3-8.

15 G. Sheldrick, *Acta Crystallogr., Sect. C*, 2015, **71**, 3-8.
